# Respiratory Syncytial Virus Infection Changes Cargo Composition of Exosome Released from Airway Epithelial Cells

**DOI:** 10.1038/s41598-017-18672-5

**Published:** 2018-01-10

**Authors:** Harendra Singh Chahar, Tiziana Corsello, Andrzej S. Kudlicki, Narayana Komaravelli, Antonella Casola

**Affiliations:** 1 University of Texas Medical Branch at Galveston, Department of Pediatrics, Galveston, 77555 USA; 2 University of Texas Medical Branch at Galveston, Department of Biochemistry and Molecular Biology, Galveston, 77555 USA; 3 University of Texas Medical Branch at Galveston, Sealy Center for Vaccine Development, Galveston, 77555 USA; 4 University of Texas Medical Branch at Galveston, Sealy Center for Molecular Medicine, Galveston, 77555 USA

## Abstract

Exosomes are microvesicles known to carry biologically active molecules, including RNA, DNA and proteins. Viral infections can induce profound changes in exosome composition, and exosomes have been implicated in viral transmission and pathogenesis. No information is current available regarding exosome composition and function during infection with Respiratory Syncytial Virus (RSV), the most important cause of lower respiratory tract infections in children. In this study, we characterized exosomes released from RSV-infected lung carcinoma-derived A549 cells. RNA deep sequencing revealed that RSV exosomes contain a diverse range of RNA species like messenger and ribosomal RNA fragments, as well as small noncoding RNAs, in a proportion different from exosomes isolated from mock-infected cells. We observed that both RNA and protein signatures of RSV were present in exosomes, however, they were not able to establish productive infection in uninfected cells. Exosomes isolated from RSV-infected cells were able to activate innate immune response by inducing cytokine and chemokine release from human monocytes and airway epithelial cells. These data suggest that exosomes may play an important role in pathogenesis or protection against disease, therefore understating their role in RSV infection may open new avenues for target identification and development of novel therapeutics.

## Introduction

Exosomes are a nano-sized (30–100 nm) subclass of extracellular vesicles with exposed external domains of transmembrane proteins^1–3^. Exosomes are formed during the maturation of endosomes upon invagination and budding of the limiting membrane of late endosomes as intraluminal vesicles (ILVs) of multivesicular bodies (MVBs). These small vesicles are termed ILVs while contained in MVBs and ‘exosomes’ when released in to the extracellular environment. They are secreted by virtually all cell types and their presence has been confirmed in all bodily fluids such as blood, urine, saliva, breast milk, bronchial lavage, cerebral spinal fluid and amniotic fluid^4–17^. Exosomes have been shown to contain different types of biomolecules including proteins, carbohydrates, lipids and nucleic acid signature of source of origin^18–22^. Because of that, they have emerged as an important tool for intercellular communication, giving birth to a new concept that cellular communication in mammals can be mediated by the exchange of information through the horizontal transfer of biologically active proteins, lipids and regulatory RNAs^23^. RNA and protein composition of exosomes varies in both quantity and quality depending on the type and physiological/pathological state of the cells they originate from; suggesting that recruitment of RNA and protein in to exosome is a regulated process^19,24^.

Because of the ability to carry complex biological information (proteins, lipids and RNAs), exosomes have been implicated in a variety of normal as well as pathophysiological conditions, such as lactation, immune response, neuronal function, development and progression of liver disease, neurodegenerative diseases, cancer and viral infections^14,23,25–29^. Exosome-mediated extracellular delivery of nucleic acids and proteins between virus-infected and uninfected bystander cells has been shown to play a role in viral transmission and modulation of immune responses, as some viruses exploit the exosomal pathway for their assembly/budding/release, and either suppression or activation of the immune system^30–33^.

Respiratory syncytial virus (RSV) is an important cause of acute respiratory tract infections in children, elderly and immunocompromised individuals with a huge global health impact. Each year, over 33 million children under the age of 5 years are affected by this disease, leading to over 3 million hospitalizations and almost 200,000 deaths^34^. In the United States only, over 2 million children younger than 5 years of age require medical attention because of RSV each year^35^. RSV is the most common cause of bronchiolitis and pneumonia in children younger than 1 year. Infants born preterm and children with congenital heart disease, chronic lung disease, and other high-risk medical conditions have greater risk of developing more severe RSV infection^36^. While considered a high priority, development of a safe and effective vaccine and specific treatment still remain elusive for RSV, therefore increasing our knowledge of how the virus interacts with the host is important to better understand disease pathogenesis and to develop new therapeutic approaches. In this study, we characterized exosomes released from RSV-infected airway-derived A549 cells and found that RSV induces significant changes in exosome RNA cargo composition, investigated using next generation sequencing. We observed that both RNA and protein signatures of RSV were present in exosomes, however, exosomes were not associated with infectious viral particles and were not able to establish productive infection in uninfected cells. Exosomes isolated from RSV-infected cells were able to induce chemokine release from human monocytes and A549 cells, suggesting that exosomes released during infection could alter cellular responses, leading to either suppression or activation of the innate immune system. These findings reveal a new dimension in host-pathogen interactions highlighting the possible role of extracellular vesicles in RSV infection.

## Results

### Purification and characterization of exosomes from virally-infected cells

**Figure 1:**
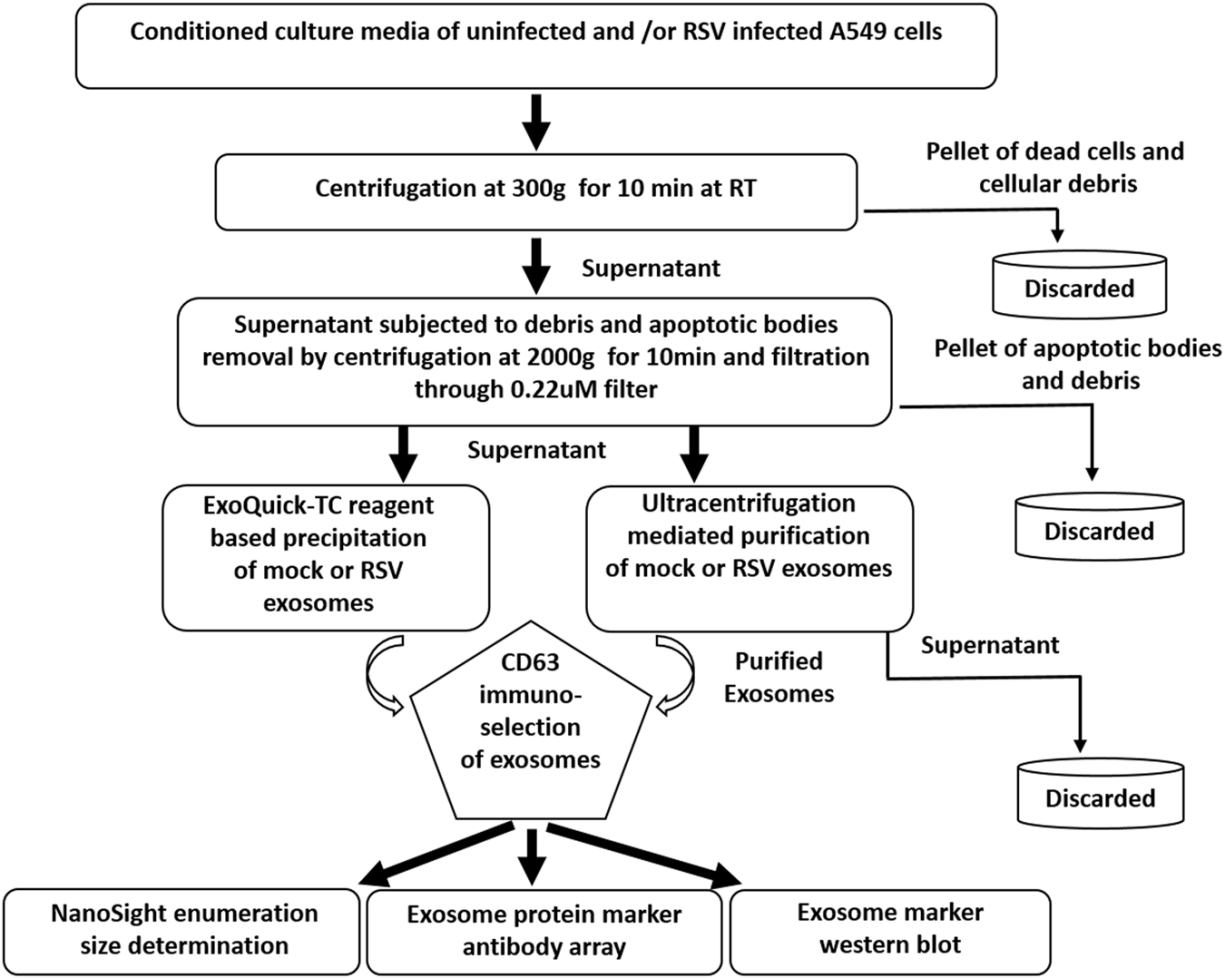
Diagram of exosome isolation and characterization. Exosomes were isolated using ExoQuick reagent from conditioned cell culture supernatants and subjected to CD63 immuno-magnetic selection for recovery of highly pure exosomes. Immuno-purified exosomes were then characterized by western blot, using an exosome marker antibody array, and by nanoparticle tracking analysis.

To investigate changes in exosome cargo composition, transmission of RNA/protein/virus to uninfected cells or in intercellular communication, a population of exosomes devoid of cellular and viral contaminants is necessary. Since RSV particles and exosome size range overlaps, traditional differential centrifugation/ultracentrifugation methods alone would not yield a pure population of exosomes^37^. For our study, we opted for a two-step exosome purification method that included precipitation reagent-based exosome enrichment, followed by CD63 antibody based immuno-magnetic isolation, as there is no evidence from the literature that RSV virions contain this particular molecule^38,39^, and because RSV assembles and egress cells through a pathway similar to influenza, which has been shown to be devoid of CD63 and other tetraspannins^40^. An illustration of exosome enrichment and purification is shown in Fig. 1.

**Figure 2:**
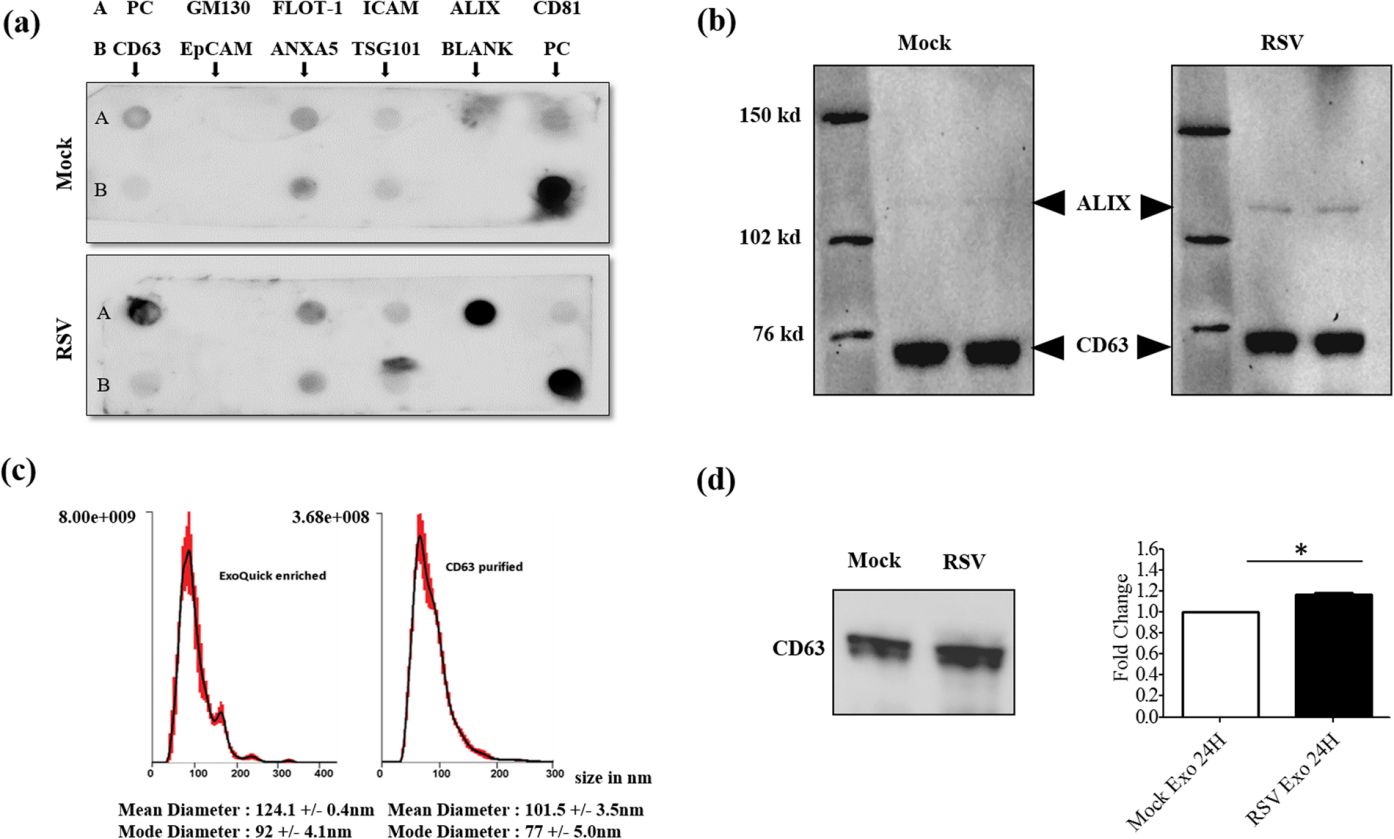
Characterization of purified exosomes. (**a**) Characterization of equal amounts of purified exosomes by protein marker antibody array. The exosome marker array detects 8 known exosome markers namely – CD63, Immunoglobulin superfamily, member 8 (CD81), Programmed cell death 6 interacting protein (ALIX), Flotillin 1 (FLOT1), Intercellular adhesion molecule 1 (ICAM1), Epithelial cell adhesion molecule (EpCam), Annexin A5 (ANXA5) and Tumor susceptibility gene 101 (TSG101). Cis-Golgi matrix protein marker GM130 serves as control to monitor cellular contamination in exosome preparations. PC stands for positive control. (**b**) Western blot of equal amounts of CD63-purified exosomes using the exosome markers ALIX and CD63. (**c**) Absolute size determination and quantification of RSV exosomes either reagent enriched or CD63-purified by NanoSight LM10 analysis. The particles were tracked and sized based on Brownian motion and the diffusion coefficient. The mean and mode diameter of exosomes particles are shown. The absolute count of exosome particles was determined and expressed as particles/ml. (**d**) Exosomes were enriched from 24 h cell supernatants and equal volumes were analyzed for CD63 expression by Western blot assay. Right panel represents densitometric analysis of three independent experiments. ‘*’ Indicates a statistically significant difference (P value < 0.05) comparing RSV exosomes versus mock.

Following purification, exosomes isolated from mock- (mock-infected) and RSV-infected cells were subjected to characterization using three different methods. First, we confirmed presence of common exosome markers using an antibody array. We found that, seven out of eight exosome marker proteins detected by the array, namely CD63, CD81, ALIX, FLOT1, ICAM1, ANXA5 and TSG101, were present in our exosome preparations. EpCAM was not detected in our preparations. The cis-Golgi marker GM130, included to monitor cellular contamination from other membrane compartments, was also absent as expected (Fig. 2a). Interestingly, ALIX was strongly upregulated in exosomes isolated from virus-infected cells, compared to mock-infected. We then confirmed the presence of the exosome markers CD63 and ALIX by western blot (Fig. 2b), which also showed an enrichment of ALIX in exosomes derived from infected cells. The exosomes were further analyzed by nanoparticle tracking analysis (NTA) and compared to exosomes enriched by Exoquick precipitation but not immunopurified. The size range of Exoquick-enriched exosomes varied from 30 to 200 nm, with a median diameter of 124.1 +/− 0.4 nm and a mode diameter of 92 +/− 4.1 nm. On the other hand, the exosome population obtained after CD63 purification was more uniform in terms of size and most exosomes were in the size range of 50–150 nm with a median diameter of 101.5 +/− 3.5 nm and mode diameter of 77 +/− 5.0 nm (Fig. 2c). Finally, equal volumes of exosomes enriched by Exoquick were analyzed for CD63 expression by western blot (Fig. 2d). There was a slight increase in CD63 levels in RSV vs mock exosomes, which was however statistically significant, suggesting that infection could enhance exosome release.

### Exosomes derived from virus-infected cells contain RSV components but do not transmit RSV infection

**Figure 3:**
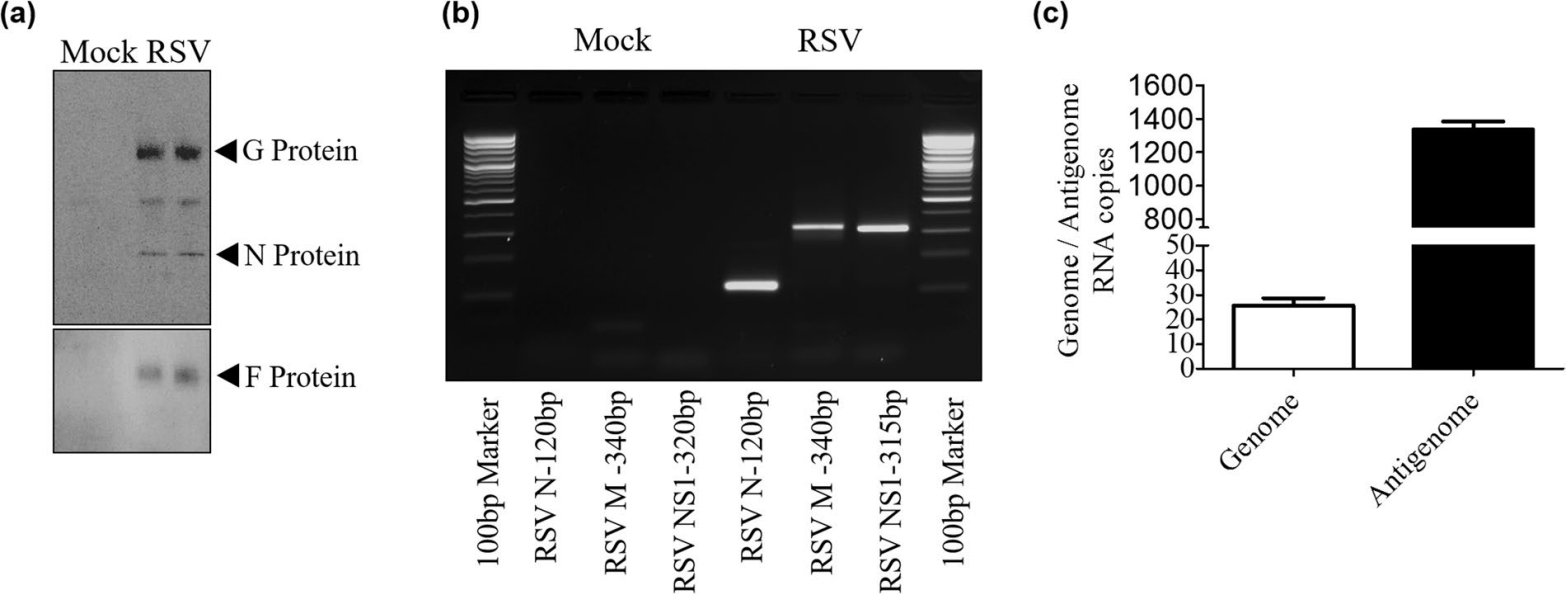
RSV viral RNA and protein content in exosomes. (**a**) Western blot of protein lysates of exosomes using a polyclonal antibody against RSV. (**b**) RSV N, M and NS1 gene amplification by RT-PCR from CD63-purified exosome RNA. (**c**) qRT-PCR amplification of genomic and antigenomic RNA present in RSV exosomes.

Exosomes have been shown to carry pathogen signatures of infected cells, along with host cellular components, as reported for certain viral, bacterial and parasitic infections^41–46^. Both viral proteins and RNAs have been identified in exosomes obtained from infected cells^31,44,46^. To investigate if exosomes carry RSV protein, lysates of CD63 immuno-purified exosomes from virus- and mock-infected A549 cells were analyzed by western blot. We found that exosomes isolated from virus-infected cells contained RSV nucleocapsid protein N, attachment protein G and fusion protein F (Fig. 3a). To determine whether exosomes derived from infected A549 cells would carry viral RNA, RT-PCR for RSV N, M and NS1 gene detection was performed on RNA isolated from CD63-purified exosomes. All three genes were amplified by RT-PCR in RSV exosomes but not in exosomes of mock-infected cells (Fig. 3b). We also tested the presence of RSV genome and antigenome using specific primers for amplification of intergenic regions and found that both of them were detectable in RSV exosomes, although the majority was antigenomic in nature (Fig. 3c).

In the case of Hepatitis C, exosomes containing full-length viral RNA, along with core and envelope proteins, were shown to be infectious and a major route of transmission^45^. As we found presence of viral RNAs and proteins in exosomes isolated from infected cells, we investigated whether these exosomes could establish a productive infection in naive cells. Purified exosomes from RSV-infected cells were inoculated on to highly susceptible Hep-2 cells, a cell line routinely used for RSV propagation, which were observed for the presence of typical cytopathic effect and harvested at different day p.i., up to day 14, to assess viral replication. We were not able to detect significant virus replication associated with the exosome preparations of RSV-infected cells.

### Exosome RNA cargo comprises short RNA sequences and is protected from degradation

**Figure 4:**
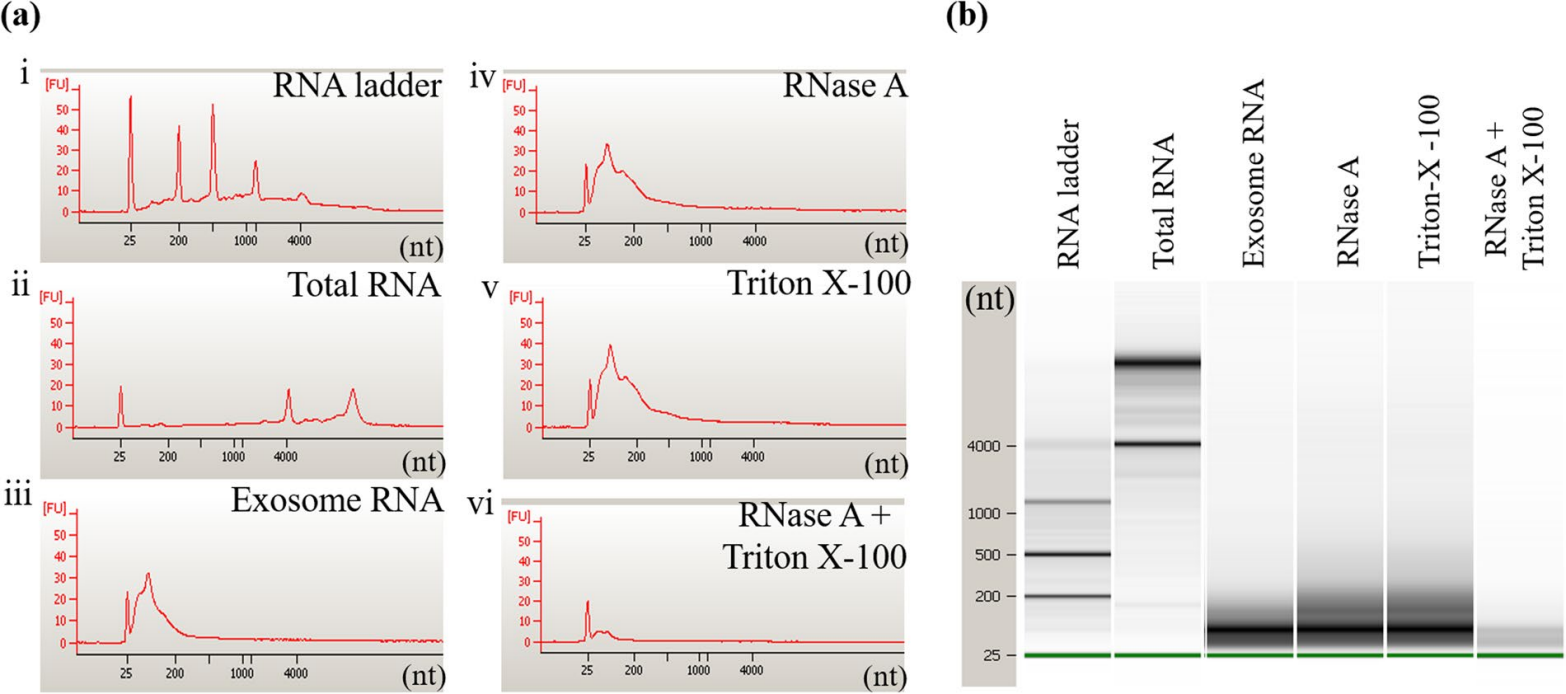
Exosome RNA cargo length profile. (**a**) Electropherograms and (**b**) gel images of RNA extracted from exosomes untreated or treated with RNase A in the presence or absence of Triton X-100 and run on the Agilent Bioanalyzer to determine size of RNA fragments.

Exosomes carry biologically active RNA signals to recipient cells^47–49^, which need to be protected from ubiquitously present RNA exonucleases. Using the Agilent Bioanalyzer, we investigated RNA length profile and RNase protection of exosomes isolated from mock and infected cells. We observed that the length profiles of exosomal RNA was distinct from that of whole cell RNA isolated from A549 cells. As expected, the exosomal RNA cargo comprised of short RNA fragments ranging from 20–250 nucleotide in length, whereas whole cell RNA sequences were considerably long, 25 to 4000 nucleotide (nt) in size (Fig. 4a ii–iii). To serve as effective signal carrier in cell to cell communication, exosome RNA is protected from RNA exonuclease that might be present in extracellular spaces. To determine whether RNA obtained from our exosome preparations was indeed from exosomes, not just associated or co-purified during isolation, we analyzed RNA length profile of CD63-purified exosomes treated with enzyme RNase A in the presence or absence of a membrane-permeabilizing detergent (Triton X-100) using Agilent Bioanalyzer. The results showed that exosomal RNA remained intact following treatment with RNase in the absence of the detergent (Fig. 4a iv), but was degraded in the presence of Triton X-100 (Fig. 4a vi). Triton X-100 alone had no effect on exosome RNA cargo integrity (Fig. 4a v). These findings indicate that the RNA was confined within the exosomal membrane and thereby protected from degradation.

### Small RNA profiling in exosomes derived from mock- and virus-infected cells

**Figure 5:**
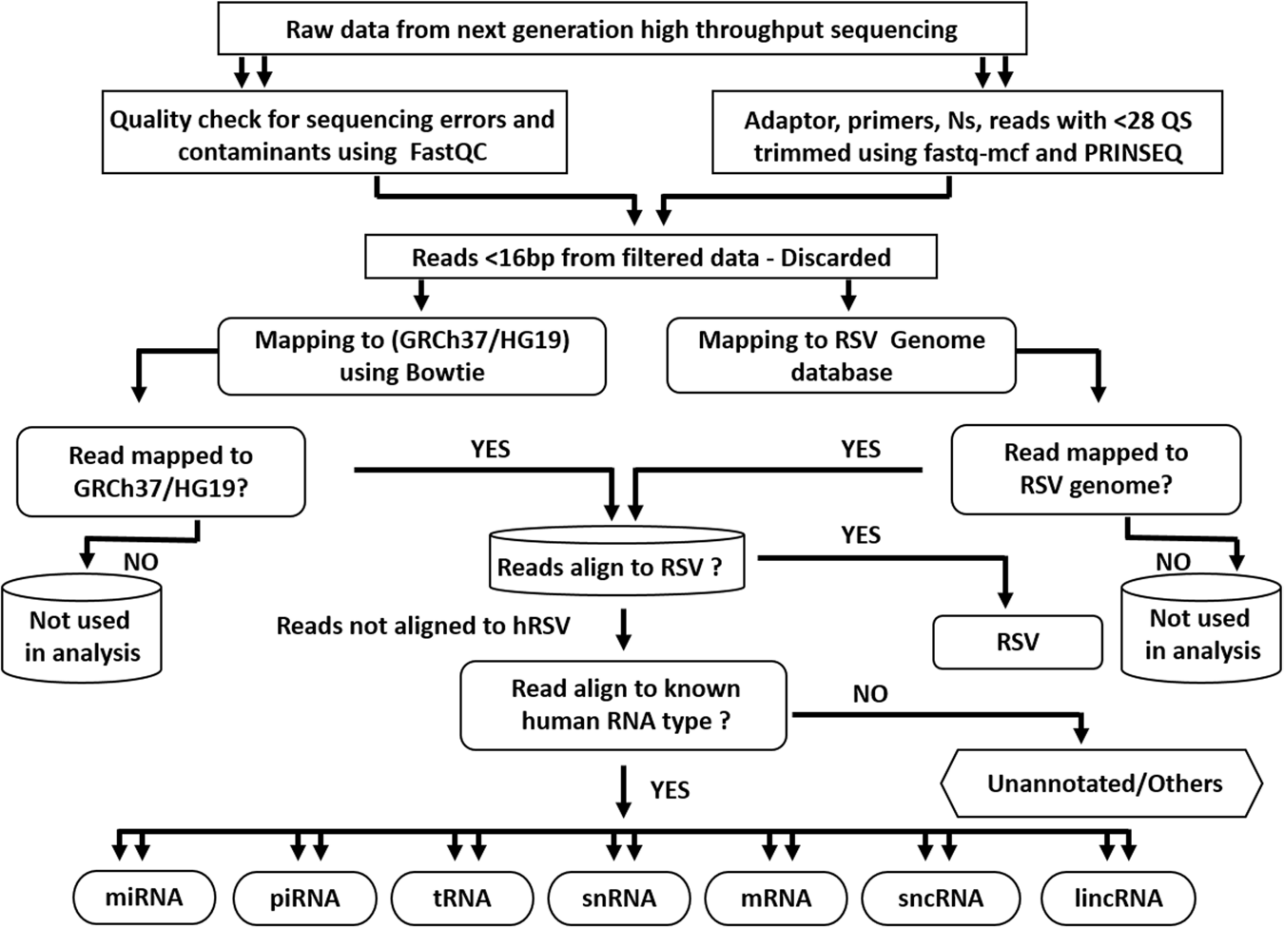
Next generation high-throughput RNA sequencing analysis pipeline flowchart. The flowchart demonstrates how the next generation sequencing data was analyzed. In brief, raw sequencing reads were quality checked for sequencing errors and contaminants using FastQC. Adapter sequences, primers, Ns, and reads with quality score below 28 were trimmed using fastq-mcf of ea-utils and PRINSEQ. Reads < 16 bp after trimming were discarded. Pseudo single-end reads were mapped to the human genome using bowtie. Raw read counts were calculated for known gene categories including ncRNAs, antisense transcripts, coding and intronic regions of mRNAs, and repeats. Annotations of known genes were retrieved from miRBase release 20, NCBI RefSeq, Human lincRNA Catalog, and UCSC Genome Browser. hRSV- Human Respiratory Syncytial Virus, miRNA- microRNA, piRNA- piwi interacting RNA, tRNA- transfer RNA, snRNA- small nucleolar RNA, mRNA-messenger RNA, sncRNA-small noncoding RNA, lincRNA- long intergenic noncoding RNA.

Next generation high throughput sequencing (NGS) technologies have been utilized extensively to gain insights into small noncoding RNAs/regulatory RNAs in several species^50–54^. Virus infections are known to induce significant changes in host cells to facilitate their replication and transmission and it has been shown that virus infections induce changes in composition of exosomal RNA^31,44,46^. To compare RNA cargo of exosomes released from mock-infected and RSV-infected A549 cells, total exosomal RNA was isolated and small RNAs were sequenced and analyzed as described in methods. NGS analysis pipeline flowchart is shown in Fig. 5. RNA cargo of exosomes isolated from mock- and RSV-infected cells was composed of a diverse range of RNA types, which were present in both sets of exosomes although their quantities were different in the two sets (Table 1). In exosomes isolated from mock-infected A549 cells, antisense sequences to repeat elements (23.126%), antisense to introns (18.575%), RefSeq introns (16.473%), short interspersed nuclear elements (SINE) (10.080%) formed the largest part of sequences present (68.254%). Sequences that did not map to any known region of human genome were represented as unannotated and formed 13.790% of total RNA reads. The remaining reads (17.956%) were from antisense to exons, antisense to non-coding RNAs (ncRNA), CDBox Small nucleolar RNA, HAcaBox Small nucleolar RNA, long intergenic non-coding RNA (lincRNA), large interspersed nuclear elements (LINE), long terminal repeats (LTR), microRNA (miRNA), Other Rfam ncRNA, piwi-interacting RNA (piRNA), RefSeq exons, RefSeq lncRNA, ribosomal RNA (rRNA), small Cajal body-specific RNAs (scaRNA), Tandem repeat, transfer RNA (tRNA), tRNA-like. RSV infection resulted in considerable changes in the proportion of RNA types recruited to the exosomes, with antisense sequences to repeat elements, exons and introns, RefSeq intron and unannotated sequences present at a significantly lower level compared to mock exosomes. On the other hand, SINE and rRNA sequences increased considerably in exosomes from virus-infected cells. To graphically demonstrate the changes in RNA type packaging to exosomes, we grouped the RNAs into five broad categories namely genome, small non-coding (nc) RNA, repeat elements, ribosomal RNA and unannotated RNA sequences. The complete list with read counts of the identified RNA types is provided in Supplementary Data 1 excel file (SD1).

**Table 1:** Percentage of RNA types in exosomes derived from mock- and RSV-infected A549 cells derived by dividing the RNA type specific reads by total number of mapped reads.

RNA type	Percentage in Mock Exosomes	Percentage in RSV Exosomes
Antisense to exons	1.7	0.3
Antisense to introns	18.6	10.8
Antisense to ncRNAs	3.4	2.1
Antisense to repeat elements	23.1	10.8
CDBox	0.003	0.05
HAcaBox	0	0.006
lincRNA	0.155	0.043
LINE	4.2	0.4
LTR	2.4	0.1
miRNA	0.04	0.3
Other Rfam ncRNA	0.4	5.7
piRNA	0.1	0.8
RefSeq exons	1.8	0.2
RefSeq introns	16.5	9.7
RefSeq lncRNA	0.5	14.2
rRNA	0.9	24.1
scaRNA	0	0.002
SINE	10.1	18.4
Tandem repeat	0	0
tRNA	1.3	0.7
tRNA_like	1.1	0.4
Unannotated	13.8	1.0

### Small ncRNAs in exosomes isolated from RSV infected A549 cells

**Figure 6:**
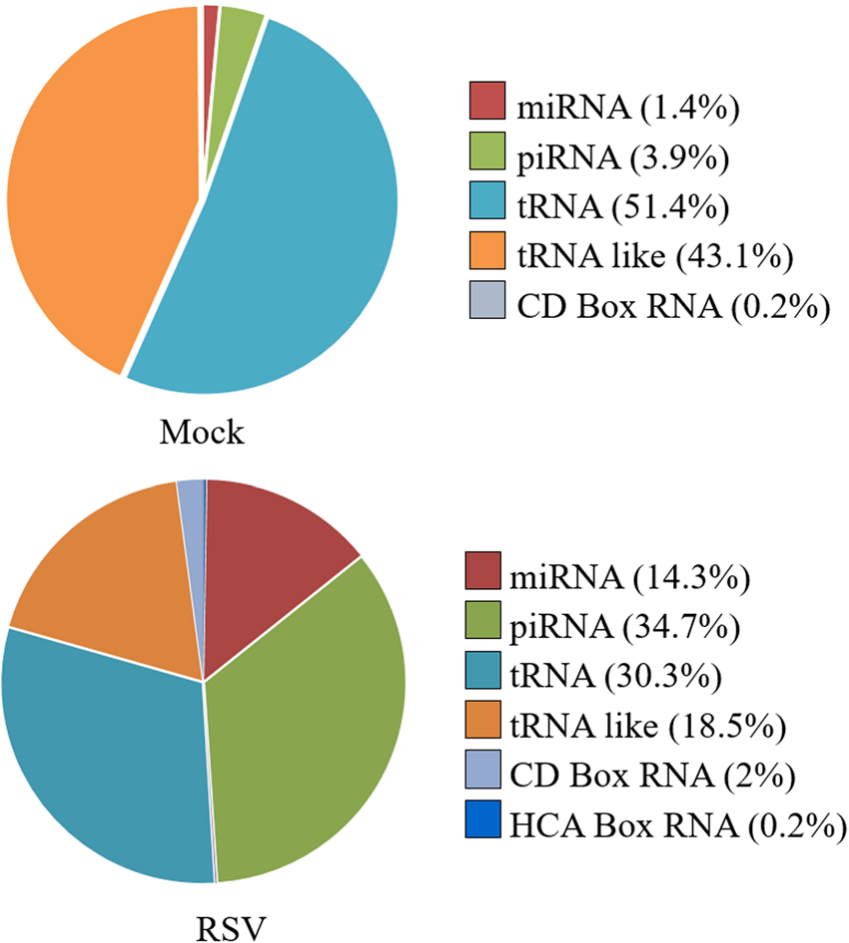
Graphic representation of the relative abundance of small ncRNAs from exosomes.

Although the small ncRNA sequences represented a smaller fraction of the total RNA reads in both mock and RSV exosome populations, they contain important regulatory RNAs such as miRNA, piwi RNA and tRNA, therefore we analyze these RNA species separately from the others (Fig. 6). tRNA and tRNA-like sequences formed most of the RNA sequences in mock exosomes representing more than 90% of RNA reads in the small RNA category. MiRNA and piRNA formed ~1.5% and ~3.9% of total small RNA reads in mock exosomes. There was a significant increase in miRNA and piRNA levels in exosome isolated from infected cells, indicating that RSV infection was associated with profound changes in exosome miRNA and piRNA content (Fig. 6). Looking at the expression profiles of miRNAs with >10 reads, a total of 100 miRNAs were detected between mock and RSV exosomes. Out of these 100, 66 miRNAs were present in both mock and RSV exosomes, with 56 significantly upregulated and 10 downregulated in exosomes isolated from virus-infected cells, compared to mock. The top miRNAs upregulated (≥4 fold) and downregulated (≥2 fold) are shown in Table 2. Of the remaining 34, 25 miRNA were only detected in RSV exosomes, and 9 miRNA in mock exosomes (Table 3). The complete list of miRNAs whose expression in exosomes was changed following infection is shown in Supplementary Table 1 (ST1).

**Table 2:** Top 20 miRNAs upregulated (≥4 fold) and 10 miRNAs downregulated (≥2 fold) in RSV exosomes.

S. No.	miRNA	Read Count (Avg)		Log2 Fold
		Mock Exo	RSV Exo	Change
1	hsa-mir-6087	45.59	39800.59	9.769
2	hsa-let-7e	0.62	468.17	9.565
3	hsa-miR-182-5p	0.25	43.34	7.433
4	hsa-miR-181b-5p	0.62	77.21	6.965
5	hsa-mir-128-1	0.25	14.68	5.872
6	hsa-miR-19b-3p	0.25	13.24	5.722
7	hsa-mir-23b	1.14	54.52	5.585
8	hsa-let-7i	12.76	610.07	5.578
9	hsa-mir-22	2	79.13	5.303
10	hsa-miR-24-3p	5.98	229.59	5.263
11	hsa-mir-182	1.39	45.8	5.046
12	hsa-mir-378a	0.87	28.12	5.017
13	hsa-mir-99b	1.14	35.93	4.983
14	hsa-miR-320b	2.62	74.63	4.831
15	hsa-let-7f-1	22.5	590.86	4.714
16	hsa-miR-4516	2.97	77.27	4.700
17	hsa-mir-21	121.4	3155.14	4.699
18	hsa-mir-30c-2	2.1	42.72	4.343
19	hsa-miR-423-5p	2.24	44.06	4.298
20	hsa-miR-320c	1.75	34.03	4.278
21	hsa-mir-223	21	0.17	−6.911
22	hsa-mir-2964a	62.14	0.57	−6.767
23	hsa-mir-205	18.17	0.29	−5.993
24	hsa-mir-143	65.39	1.41	−5.532
25	hsa-miR-3168	222.71	4.82	−5.530
26	hsa-miR-146a-5p	15.8	0.46	−5.102
27	hsa-miR-10b-5p	56.07	4	−3.809
28	hsa-mir-328	21.77	1.83	−3.575
29	hsa-miR-1246	237.74	23.03	−3.367
30	hsa-miR-185-5p	17.3	6.91	−1.324

**Table 3:** miRNAs detected either in RSV-exosomes or mock-exosomes.

S. No.	miRNA	Read Count (Avg)	
		Mock Exo	RSV Exo
1	hsa-mir-31	0	529.15
2	hsa-mir-3687	0	114.57
3	hsa-mir-222	0	80.93
4	hsa-mir-151a	0	66.10
5	hsa-miR-4449	0	65.29
6	hsa-mir-663a	0	56.03
7	hsa-mir-196a-2	0	53.41
8	hsa-mir-4508	0	37.12
9	hsa-mir-1248	0	34.64
10	hsa-miR-4497	0	28.19
11	hsa-mir-1273f	0	28.08
12	hsa-mir-28	0	26.67
13	hsa-mir-191	0	20.44
14	hsa-miR-3648	0	17.54
15	hsa-miR-194-5p	0	17.14
16	hsa-miR-128	0	14.30
17	hsa-mir-1307	0	13.91
18	hsa-mir-744	0	13.07
19	hsa-miR-148b-3p	0	12.31
20	hsa-miR-1307-3p	0	12.02
21	hsa-mir-663b	0	11.33
22	hsa-miR-152	0	11.19
23	hsa-mir-421	0	10.99
24	hsa-mir-7-1	0	10.43
25	hsa-mir-486	0	10.26
26	hsa-miR-1321	50.50	0
27	hsa-mir-204	34.07	0
28	hsa-mir-637	22.18	0
29	hsa-mir-4689	19.39	0
30	hsa-mir-144	15.38	0
31	hsa-miR-4269	11.12	0
32	hsa-mir-885	10.50	0
33	hsa-mir-4795	10.50	0
34	hsa-mir-718	10.29	0

To characterize potential targets of miRNAs identified in exosomes, we integrated the lists of microRNAs with target predictions available from the TargetScan version 7.1, containing 1,387,426 human miRNA-target interactions. We identified 4,816 potential targets of miRNAs upregulated in the exosomes following RSV infection, 925 potential targets of miRNAs downregulated in exosomes and 2,122 targets of miRNAs highly abundant in exosomes (see ST2, ST3 and ST4). We then characterized the most prevalent functions of the predicted target genes of the identified miRNAs using the Gene Ontology functional classification, focusing on molecular function, biological processes and pathways. We found that the most significant groups of targets of regulated miRNAs are related to DNA-binding, transcriptional and post-transcriptional regulation, alternative splicing, chromatin modification, and embryonic development. A summary of the top twenty function/processes are presented in Tables 4 and 5, while the complete analysis is provided as nine supplementary excel files (SD2 to SD10).

**Table 4:** Top biological processes from potential miRNA targets of Tables 2 and 3.

Table 2	Table 3
GO:0045944~positive regulation of transcription from RNA polymerase II promoter	GO:0045893~positive regulation of transcription
GO:0000122~negative regulation of transcription from RNA polymerase II promoter	GO:0045944~positive regulation of transcription from RNA polymerase II promoter
GO:0006351~transcription	GO:0000122~negative regulation of transcription from RNA polymerase II promoter
GO:0045893~positive regulation of transcription	GO:0006366~transcription from RNA polymerase II promoter
GO:0045892~negative regulation of transcription	GO:0006351~transcription
GO:0006468~protein phosphorylation	GO:0045892~negative regulation of transcription
GO:0006366~transcription from RNA polymerase II promoter	GO:0006468~protein phosphorylation
GO:0023014~signal transduction by protein phosphorylation	GO:0042472~inner ear morphogenesis
GO:0006355~regulation of transcription	GO:0007507~heart development
GO:0016569~covalent chromatin modification	GO:0048015~phosphatidylinositol-mediated signaling
GO:1900740~positive regulation of protein insertion into mitochondrial membrane involved in apoptotic signaling pathway	GO:0007173~epidermal growth factor receptor signaling pathway
GO:0048015~phosphatidylinositol-mediated signaling	GO:0035335~peptidyl-tyrosine dephosphorylation
GO:0006915~apoptotic process	GO:0030097~hemopoiesis
GO:0009952~anterior/posterior pattern specification	GO:0001525~angiogenesis
GO:0043525~positive regulation of neuron apoptotic process	GO:0031175~neuron projection development
GO:0060021~palate development	GO:0018105~peptidyl-serine phosphorylation
GO:0016055~Wnt signaling pathway	GO:0006607~NLS-bearing protein import into nucleus
GO:0010468~regulation of gene expression	GO:0009952~anterior/posterior pattern specification
GO:0035278~miRNA mediated inhibition of translation	GO:0035194~posttranscriptional gene silencing by RNA
GO:0035194~posttranscriptional gene silencing by RNA	GO:0007165~signal transduction

**Table 5:** Top molecular functions from potential miRNA targets of Tables 2 and 3.

Table 2	Table 3
GO:0005515~protein binding	GO:0005515~protein binding
GO:0003700~transcription factor activity	GO:0003700~transcription factor activity
GO:0003677~DNA binding	GO:0043565~sequence-specific DNA binding
GO:0008134~transcription factor binding	GO:0001077~transcriptional activator activity
GO:0001077~transcriptional activator activity	GO:0004672~protein kinase activity
GO:0004674~protein serine/threonine kinase activity	GO:0004674~protein serine/threonine kinase activity
GO:0046332~SMAD binding	GO:0044212~transcription regulatory region DNA binding
GO:0046872~metal ion binding	GO:0008270~zinc ion binding
GO:0008270~zinc ion binding	GO:0008134~transcription factor binding
GO:0043565~sequence-specific DNA binding	GO:0004702~receptor signaling protein serine/threonine kinase activity
GO:0003730~mRNA 3′-UTR binding	GO:0000978~RNA polymerase II core promoter proximal region sequence-specific DNA binding
GO:0000978~RNA polymerase II core promoter proximal region sequence-specific DNA binding	GO:0003714~transcription corepressor activity
GO:0004702~receptor signaling protein serine/threonine kinase activity	GO:0004725~protein tyrosine phosphatase activity
GO:0003682~chromatin binding	GO:0042826~histone deacetylase binding
GO:0004842~ubiquitin-protein transferase activity	GO:0008013~beta-catenin binding
GO:0044325~ion channel binding	GO:0019838~growth factor binding
GO:0003714~transcription corepressor activity	GO:0003682~chromatin binding
GO:0005524~ATP binding	GO:0003677~DNA binding
GO:0044212~transcription regulatory region DNA binding	GO:0005524~ATP binding
GO:0004843~thiol-dependent ubiquitin-specific protease activity	GO:0000979~RNA polymerase II core promoter sequence-specific DNA binding

**Figure 7:**
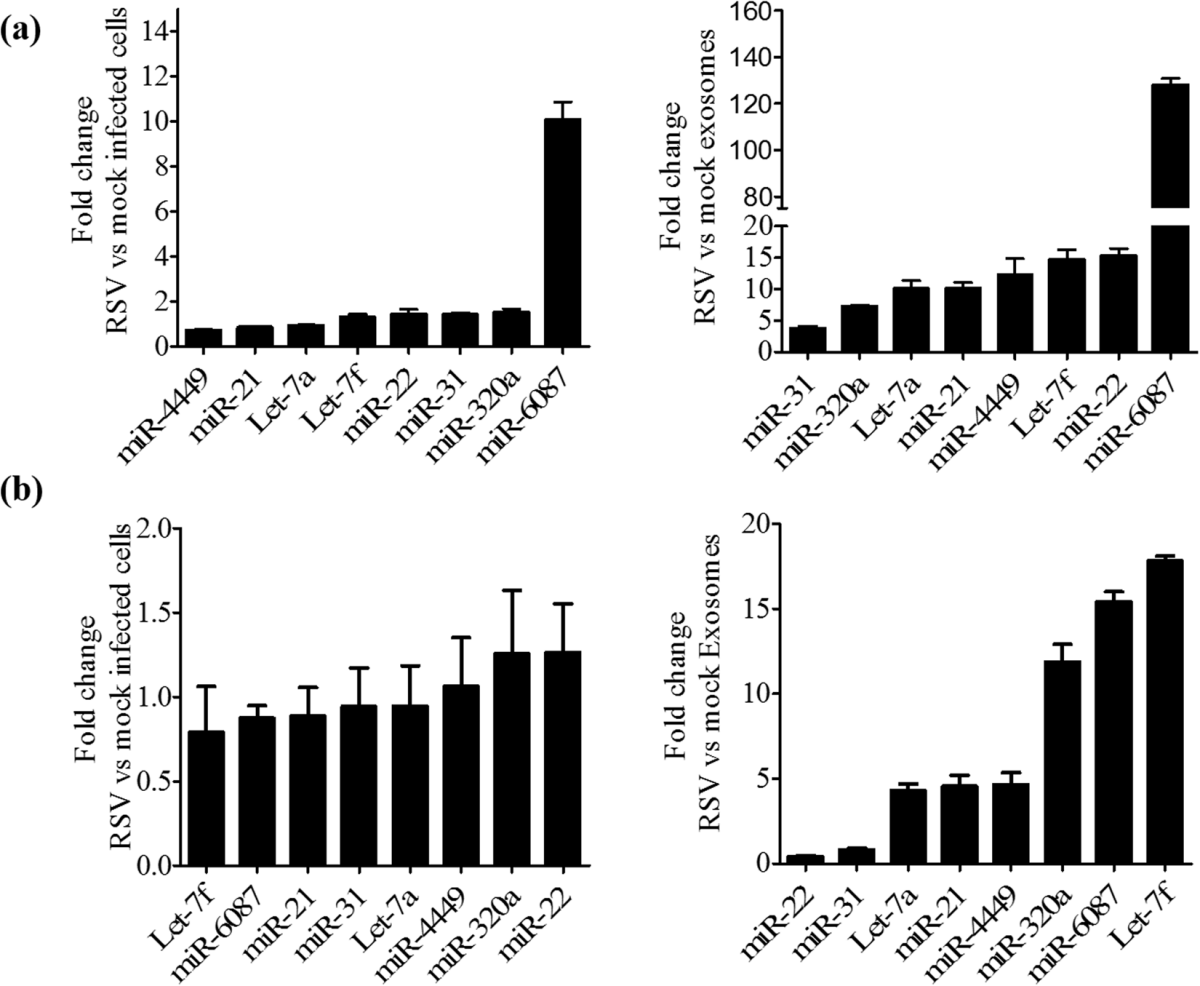
Validation of miRNA expression in cells and exosomes. RNA extracted from A549 cells (**a**) or SAE cells (**b**) mock- and RSV-infected for 24 h **(**left panels) and from mock and RSV exosomes (right panels) was subjected to miRNA analysis by RT-PCR. Fold changes in miRNA expression were determined by the 2-ΔΔCt method and represent mean ± SD normalized to small-nucleolar RNA U6 (RNU6).

To validate findings obtained by NGS, out of the upregulated miRNAs in RSV exosomes, we selected 4 miRNAs from the top upregulated (miR-6087, miR-22, Let-7f, miR-21) and 4 miRNAs with >200 reads in RSV exosomes (Let-7a, miR-31, miR-320a and miR-4449), and examined their expression by RT-PCR in mock and RSV exosomes, as well as in mock- and RSV-infected cells. We found that miR-6087, Let-7a, Let-7f, miR-320a, miR-21, miR-4449, and miR-22 were all differentially expressed in RSV-infected A549 cells, compared to mock (Fig. 7a, left panel), and that their level in RSV exosomes was significantly higher than in virally infected cells (Fig. 7a, right panel). To determine if similar viral-induced changes in exosome miRNA composition were present in normal cells, RNA was extracted from SAECs infected with RSV and harvested at 24 h p.i. and from exosomes enriched from supernatants of mock and infected SAECs. Same miRNAs investigated in A549 cells were analyzed by RT-PCR. While there was not a significant induction of the selected miRNAs in RSV-infected cells vs mock (Fig. 7b, left panel), exosomes derived from infected cells showed a high enrichment of the same targets (Fig. 7b, right panel), similar to the either in RSV-exosomes or mock-exosomes is indeed associated with changes in exosome small RNA levels and composition that are not a simple reflection of changes occurring in cells upon RSV infection.

The piRNAs have emerged as an important population of small RNAs involved in regulation of gene expression^55^. We observed that 52 piRNAs (>10 reads) were present in both mock and RSV infected exosomes, with 28 upregulated and 24 downregulated in RSV exosomes. Top up and downregulated piRNAs (≥4 fold) are listed in Table 6. We also found that 19 piRNAs were only present in RSV exosomes and 3 piRNAs exclusively detected in mock exosomes (Table 7). The complete list of piRNAs whose expression in exosomes was changed following infection is shown in Supplementary Table 5 (ST5).

**Table 6:** Top 15 highly up and downregulated piRNAs (≥4 fold) in RSV exosomes.

S. No.	piRNA	Read Count (Avg)		Log2 Fold
		Mock Exo	RSV Exo	Change
1	piR-32678	127.41	27150.88	7.735
2	piR-31623	0.62	48.19	6.285
3	piR-34736	1.74	128.93	6.21
4	piR-36169	6.52	231.03	5.147
5	piR-36170	6.52	231.03	5.147
6	piR-49143	22.5	590.86	4.714
7	piR-33748	7.13	108.2	3.922
8	piR-45371	7.13	108.2	3.922
9	piR-49144	126.89	1387.4	3.450
10	piR-33043	33.7	291.08	3.110
11	piR-33044	33.7	291.08	3.110
12	piR-34531	5.88	24.21	2.041
13	piR-34532	5.88	24.21	2.041
14	piR-34533	5.88	24.21	2.041
15	piR-34534	5.88	24.21	2.041
16	piR-59169	1364.92	6.02	−7.824
17	piR-50653	22.7	0.13	−7.473
18	piR-50654	22.7	0.13	−7.473
19	piR-50655	22.7	0.13	−7.473
20	piR-55865	22.7	0.13	−7.473
21	piR-56226	22.7	0.13	−7.473
22	piR-34756	10411.18	82.62	−6.977
23	piR-34370	28.39	0.29	−6.637
24	piR-45065	21.57	0.29	−6.241
25	piR-40249	50.07	0.74	−6.070
26	piR-40514	59.65	1.03	−5.855
27	piR-36037	85.04	1.78	−5.578
28	piR-34916	29.19	0.63	−5.524
29	piR-56647	40.04	1.03	−5.280
30	piR-59089	10.75	0.29	−5.236

**Table 7:** piRNAs detected either in RSV-exosomes or mock-exosomes.

S. No.	piRNA	Read Count (Avg)	
		Mock Exo	RSV Exo
1	piR-31703	0	254.19
2	piR-33650	0	209.91
3	piR-40982	0	209.39
4	piR-32079	0	209.21
5	piR-31961	0	208.91
6	piR-33527	0	153.92
7	piR-33526	0	153.02
8	piR-36716	0	127.96
9	piR-36715	0	127.67
10	piR-36714	0	127.39
11	piR-36494	0	127.01
12	piR-36713	0	125.77
13	piR-35059	0	124.97
14	piR-34608	0	118.37
15	piR-48517	0	118.37
16	piR-60668	0	58.07
17	piR-61919	0	46.01
18	piR-31701	0	36.7
19	piR-34221	0	21.87
20	piR-61950	19.15	0
21	piR-59554	13.59	0
22	piR-61974	11.12	0

### Exosomes isolated from virally-infected cells induce proinflammatory mediator secretion in by-stander cells

**Figure 8:**
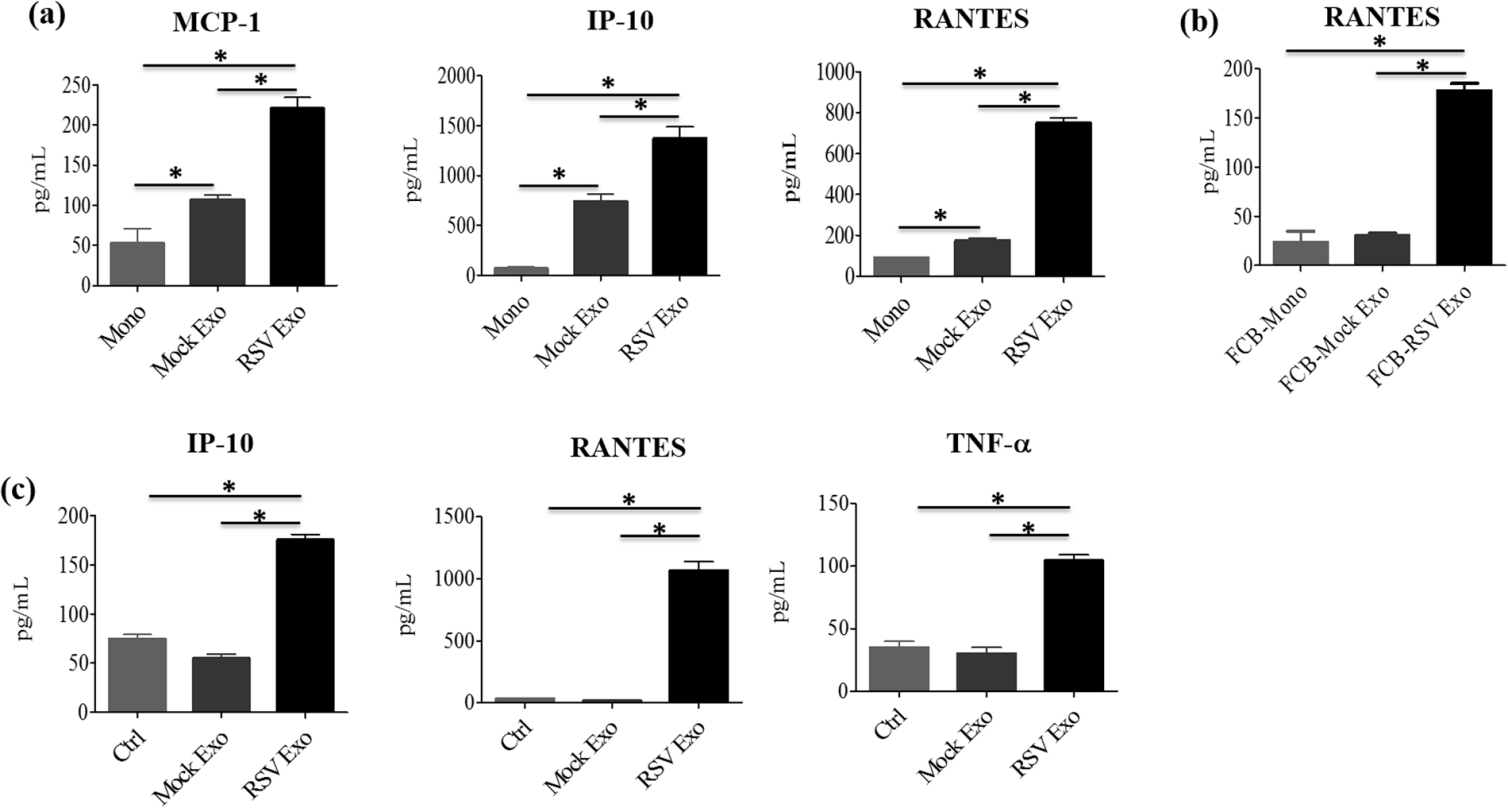
Proinflammatory mediator secretion by monocytes and A549 cells in response to exosome stimulation. Human monocytes were stimulated with mock or RSV exosomes and harvested 24 h later to collect cell supernatants to measure cytokine and chemokine secretion by cytokine multiplex assay (**a**) or ELISA (**b**). Similar experiment was performed in A549 cells **(c)**. Data are expressed as mean ± SEM, and ‘*’ indicates a statistically significant difference (*P* value < 0.05).

Exosomes have been shown to modulate the state of naïve cells by transferring viral or host components from neighboring infected cells to uninfected cells, leading to activation or inhibition of immune responses^56,57^. To determine whether exosomes isolated from RSV-infected A549 cells would exert a similar effect on innate cell responses, human monocytes were isolated from PBMCs and stimulated with purified exosomes to measure levels of eight cytokine and chemokine production in cell culture media. We found that exosomes isolated from virus-infected cells induced significantly higher secretion of MCP-1, IP-10, and RANTES in monocytes (Fig. 8a), compared to mock exosomes. To determine whether cytokine/chemokine release from exosome treated monocytes was due to Fc receptor mediated stimulation, monocytes were treated with Fc block reagent prior stimulation with exososomes and RANTES levels was measured in cell supernatant by ELISA. We found that RSV exosomes still induced a significant amount of RANTES secretion, compared to mock exosome-stimulated monocytes, indicating that chemokine release was not Fc receptor-dependent (Fig. 8b). We also tested A549 cells response to mock and RSV exosomes. Similar to monocytes, addition of RSV exosomes to A549 cells induced significantly higher levels of RANTES, IP-10 and TNF-α secretion, compared to mock exosomes (Fig. 8c), indicating that exosomes released from virus-infected cells carry biological signals that can alter innate immune responses during RSV infection.

## Discussion

The present study is the first to characterize exosomes derived from RSV-infected A549 cells, which is widely used as an *in vitro* model of paramyxovirus infection of airway epithelial cell line. We found that RSV induces significant changes in exosome RNA cargo composition, as exosomes isolated from RSV-infected cells carry both viral proteins and viral RNA along with host mRNA and rRNA fragments and small non-coding RNAs, miRNAs, piRNAs and tRNAs, in a proportion different from exosomes isolated from mock-infected cells. We also found that exosomes isolated form infected cells could activate innate immune responses by inducing the release of proinflammatory mediators, however, they were not able to establish new infection in bystander cells.

Characterization of exosome cargo composition and exosome functions requires a fairly pure population of exosomes. Although density gradient centrifugation is the most common procedure for exosome purification^37^, similar methods are used for RSV purification as well^58^ and density of RSV virions is quite close to the density of exosomes^18,58^. The two-step purification approach we used is similar to the one used in a recent study to prepare exosomes from HCV infected cells^46^, and yielded a population of vesicles of appropriate size, carrying multiple known exosome markers, devoid of replicating virus. Interestingly, exosomes isolated from RSV-infected cells showed higher levels of ALIX expression. The formation of the ILVs within the MVBs and the budding of enveloped virions share many features. Although RSV does not utilize for budding the endosomal sorting complexes required for transport (ESCRT) pathway, which is necessary for MVB formation and ILV release, differently from other viruses such as herpes, hepatitis B and C and Ebola^59–61^, our finding suggests that these pathways do interact with each other.

Similar to what reported in other studies, RNA cargo of exosomes isolated from A549 cells was stable and resistant to degradation by ribonucleases^62,63^. Although it has been reported that exosomes may carry full length mRNA sequences along with small regulatory RNAs^18,64^, we found that most of the RNA content was represented by short RNA sequences of 25–250 nucleotides. An increasing number of studies on exosomes have shown that viruses can induce selective packaging of viral RNAs and proteins to exosomes^29,31,44,61^. Exosomes from HIV-1 infected cells carry viral transactivating response (TAR) element and viral microRNAs^44,65^, while exosomes released from HCV-infected cells contain HCV RNA, as well as HCV core proteins, and they are able to establish a productive infection in uninfected cells^56^. We found that exosomes released from virus-infected cells indeed carried both RSV RNAs and proteins, however they were not able to transmit infection to recipient cells, most likely due to inability to carry a competent full length genome.

When we sequenced RNA from mock and RSV exosomes, we found in both populations a variety of small noncoding RNAs, along with other RNA type fragments such as rRNA. miRNAs are an important class of small regulatory RNAs associated with either degradation or translational repression of target mRNAs^66,67^. Multiple studies suggest that miRNAs do not always remain cellular, but are also released in to extracellular spaces via exosomes and can be readily detected in exosomes isolated from various bodily fluids^68,69^. In a recent study, exosomes released by Epstein Bar Virus (EBV)-infected cells were shown to deliver functional miRNAs to uninfected monocyte-derived dendritic cells and could mediate inhibition of expression of specific target genes^31^. RSV infection led to upregulation and downregulation, of a number of miRNAs in exosomes, many of them not known to be affected by the infection at the cellular level. Let-7f, -7i, miR-24, -31 and -221 have been shown to be upregulated in infected epithelial cells or nasal mucosa of infected infants^70–72^. miR-221 and Let-7f have been recently shown to modulate RSV replication in epithelial cells^70,71^, while miR-24 expression facilitate porcine reproductive and respiratory syndrome virus (PRRSV) infection^73^. Although most of the data were obtained in exosomes derived from a carcinoma-derived cell line, we found that the profile of enriched miRNAs was quite similar in exosomes isolated from infected normal SAECs, which we have extensively shown to behave very similar to A549 cells^74–77^.

Target prediction analysis using TargetScan^78^ suggested that miR-6087 could possibly target multivesicular body subunit 12B (MVB12B), which is a component of the ESCRT-I complex and is involved in sorting of cargos into multivesicular bodies, and coiled-coil domain containing 36 (CCDC36) gene involved in positive regulation of host antiviral defense response. Similarly, miR-182-5p target include Flotillin 1 (FLOT-1) which is a component of exosomes, miR-24-3p may target apoptosis facilitator BCL2-like11, and interferon gamma (IFNG) genes. Argonaute RISC catalytic subunit 2 (Ago2) RNA was found to have a conserved sites for miR-99-5p in its 3′UTR. The Gene Ontology functional classification found that the most significant groups of targets of regulated miRNAs are related to DNA-binding, transcriptional and post-transcriptional regulation, alternative splicing, chromatin modification, and embryonic development. Extensive experimental studies are needed to determine the exact role of these miRNAs in general and specifically in RSV infection.

Expression/recruitment to exosomes of other classes of regulatory RNA, namely piRNA and tRNA, were also affected by RSV infection. We found that several piRNAs were found to be up and downregulated in RSV exosomes. PiRNAs have been implicated in transcriptional gene regulation and transgenerational epigenetic inheritance^55^, however there is recent evidence in the literature that piRNA can regulate immune mediator expression, as in the case of piRNA-30840 which binds to IL-4 pre-mRNA via sequence complementarity and mediates its decay in nuclear exosomes^79^. The role of host cell piRNA generated during the course of virus infection, whether part of the exosome cargo or not, is currently unknown. RSV infection has been recently shown to induce tRNA fragment formation and one of them, corresponding to the 5′-half of mature tRNA derived from tRNA-Glu-CTC, displays target gene repression capabilities and promotes RSV replication^80^, suggesting an additional mechanism of small non-coding RNAs to modulate viral infection.

Our study reveals a potential role for secreted exosomes in host-virus interactions during RSV infection. We found that exosomes released from RSV-infected cells induced enhanced release of the chemokines MCP-1, IP-10, and RANTES. Exosome-mediated export of host/viral components may have variety of outcomes and these observations suggest that sequestration and export of viral components/regulatory RNAs via exosomes possibly serve both as a viral strategy to evade pathogen sensing in infected cells and host strategy to induce innate responses in neighboring uninfected cells. For example, the activation of innate immune response may have antiviral effects via release of proinflammatory cytokines and apoptosis in recipient cells. On the other hand, it could also have proviral effects by having inhibitory effects on the release of proinflammatory cytokine/chemokines or by vesicular sequestration of otherwise immunostimulatory viral RNA/protein components from cellular sensing machinery of the recipient cell [reviewed in^81–83^]. The use of exosomes by RSV reveals a previously unappreciated and less characterized method by which the virus may deliver proteins and/or RNA to the host cell to manipulate host cell responses in uninfected bystander cells. A better understanding of how exosomes may influence RSV entry/replication and whether they facilitate or inhibit immune responses in the course of infection will provide valuable insights into host-pathogen interactions and possibly identify novel targets for therapy.

## Material and Methods

### Cell culture

Human airway epithelial cell line A549 (human alveolar type II cell line -American Type Culture Collection, USA) were cultured and maintained in F12K culture media, supplemented with 10% FBS (HyClone; GE Healthcare USA), 100 U/mL penicillin G, 100 μg/mL streptomycin and 2 mM glutamine. Small alveolar epithelial cells (SAECs) (Lonza Inc., San Diego, CA), derived from terminal bronchioli of cadaveric donors, were grown in small airway epithelial cell (SAEC) growth medium, containing 7.5 mg/mL bovine pituitary extract (BPE), 0.5 mg/mL hydrocortisone, 0.5 µg/mL hEGF, 0.5 mg/mL epinephrine, 10 mg/mL transferrin, 5 mg/mL insulin, 0.1 µg/mL retinoic acid, 0.5 µg/mL triiodothyronine, 50 mg/mL gentamicin and 50 mg/mL bovine serum albumin. When SAE were used for RSV infection, they were changed to basal medium, not supplemented with growth factors, 6 h prior to and throughout the length of the experiment.

### Virus stock preparation

For preparation of RSV stocks, RSV Long strain was grown in Hep-2 cells and purified by centrifugation on discontinuous sucrose gradients as described previously^84^. The virus titer of the purified RSV pools was 8–9 log10 plaque forming units (PFU)/mL using a methylcellulose plaque assay. No contaminating cytokines were found in these sucrose-purified viral preparations^85^. LPS, assayed using the limulus hemocyanin agglutination assay, was not detected. Virus pools were aliquoted, quick-frozen on dry ice/alcohol, and stored at −80 °C until used.

### Infection of epithelial cells with RSV

Before infection, cells were maintained in exosome free culture media. At 80% to 90% confluence, cell monolayers were infected with RSV at a multiplicity of infection (MOI) of 1. An equivalent amount of 30% sucrose solution was added to uninfected cells, as a control. After RSV infection, cells were maintained in exosome free medium.

### Exosome purification

Culture media collected from 2 × 10^7^ mock-infected or infected cells for 24 h was subjected to debris removal by centrifugation at 3,000 g for 15 min at 4 °C. The clear media was subjected to further cleaning by filtration through 0.22 μm sterile filter to remove any remaining debris. Exoquick-TC (System Biosciences, USA) reagent was added to the filtered media, mixed thoroughly and incubated overnight at 4 °C to precipitate the exosomes. Next morning the mixture was subjected to centrifugation at 1,500 g for 30 min, the exosome pellet was washed and resuspended in filtered PBS. To remove contaminating viral particles, exosomes were subjected to CD63 immuno-purification using CD63 exosome isolation reagents (System Biosciences, USA), following manufacturer’s instructions. The purified exosomes were eluted from the bound CD63 beads in an average of 300 μl and used for experimental procedures. Protein concentration was determined using a protein assay kit from Bio-Rad, USA. Purified exosomes were screened for presence of replicating virus, to avoid using contaminated preparations. This screening was done by plaque assay, inoculating a fraction of the exosome pool onto Hep2 cells. In the experiments performed to determine whether purified exosomes were able to establish a productive infection, we used pools devoid of replicating virus.

### Nanosight particle tracking analysis

The size and concentration of CD63 purified exosomes were analyzed using the NanoSight™ LM10-HS10 system (Malvern Instruments, UK). For analysis, a monochromatic laser beam (405 nm) was applied to the diluted exosome solution (1:100 in 0.02 μm filtered PBS) that was injected into a LM12 viewing unit using a computer controlled syringe pump. NanoSight™ tracking analysis (NTA) software version 2.3 was used to produce the mean and median vesicle size together with the vesicle concentration (in millions). Samples were measured 3 times to ensure reproducibility. The average yield of enriched exosomes collected from 2 × 10^7^.

### Western blot

Exosome samples were lysed in buffer (0.5% Triton; 300 mM NaCl; 50 mM TrisNaCl) supplemented with protease inhibitor cocktail (Roche). Equal amount of proteins (about 10 μg) were separated by SDS-PAGE and transferred onto polyvinylidene difluoride membrane. Nonspecific binding was blocked by immersing the membrane in Tris-buffered saline-Tween (TBST) blocking solution containing 5% skim milk powder. After a short wash in TBST, the membranes were incubated with the primary antibody overnight at 4 °C, followed by the appropriate secondary antibody diluted in TBST for 1 h at room temperature. Proteins were detected using enhanced-chemiluminescence assay. The primary antibody used for western blot were rabbit anti human CD63, mouse anti human ALIX (Abcam USA), and goat anti RSV (US Biologicals, USA).

### Exosome antibody array

Characterization of isolated exosomes was done by Exo-Check antibody array (System Biosciences, USA) following manufacturer’s instructions. In brief, similar quantities of exosome preparations were lysed and incubated with the array for the pre-printed antibodies to capture their respective exosome proteins. Later the array membranes were incubated with appropriate HRP conjugated secondary antibody diluted in TBST for 1 h at room temperature. Proteins were detected using enhanced-chemiluminescence assay. The array detects 8 known exosome markers namely CD63, CD81, ALIX, FLOT1, ICAM1, EpCam, ANXA5 and TSG101 and a GM130 cis-Golgi marker to monitor for other cellular compartment contamination.

### Extraction and biochemical characterization of RNA

RNA was purified from mock or RSV exosomes by phenol/chloroform extraction using all RNA-grade reagents. For this purpose, 150 uL of LETS buffer (0.1 M LiCl, 0.01 MNa2EDTA, 0.01 M Tris-Cl pH = 7.4, 0.2% SDS, Sigma-Aldrich) was added to 50 μL of exosomes resuspended in PBS followed by addition of 200 μL Ultra-Pure buffer saturated phenol pH = 7.4 (Life Technologies, USA). The mixture was vortexed vigorously and centrifuged for 2 min at 13,000 × g in a microcentrifuge at room temperature. The upper aqueous phase was collected. RNA was precipitated by addition of 0.3 M NaCl, 2 μg/mL glycogen (Ambion, Life Technologies, USA) and 75% EtOH, and overnight incubation at −20 °C. RNA was pelleted by centrifugation at 13,000 × g for 30 min at 4 °C. RNA pellets were washed with ice-cold 75% ethanol and resuspended in 10–20 μL ddH2O. RNA concentration was determined by measuring the OD260 with the nanodrop (Thermo Scientific, USA) and used for various assays.

### RNA Length Profiles

To look at length profiles of exosome-derived RNA, 2 μg of purified A549 total RNA and 1 μg of exosome RNA were first treated with 5 units DNase I (Thermo Scientific, USA) for 30 min at 37 °C.DNase was inactivated by 2.5 mM EDTA and incubation at 65 °C for 10 min followed by phenol-chloroform extraction and ethanol precipitation. RNA was resuspended in 4 μL ddH2O and RNA length profiles were obtained with the Agilent Bioanalyzer using the RNA 6000 Pico kit (Agilent Technologies, USA). To assess whether the exosomal membrane was protecting the vesicular RNA from degradation by exogenous RNases, intact exosomes, resuspended in PBS were treated with 0.4 mg/mL RNAse A for 30 mins at 37 °C in the presence or absence of 0.1% Triton X-100 (Sigma-Aldrich, USA). After incubation, samples were extracted with phenol/chloroform and RNA was ethanol precipitated. Samples were then treated with DNase, again phenol/chloroform extracted and ethanol precipitated, resuspended in 4 μL ddH2O and run on the Agilent Bioanalyzer to determine whether or not RNA had undergone degradation. RNA pico ladder (Agilent Technologies, USA) served as untreated RNA control along with A549 cell RNA.

### Reverse Transcription (RT)-PCR

For reverse transcription, 500 ng of isolated RNA was subjected to cDNA synthesis using SuperScript® III First-Strand Synthesis system (Thermo Scientific, USA) following manufactures instructions. 1ul cDNA was used as template for gene amplification. For PCR amplification of viral genes, REDTaq^®^ ReadyMix^™^ PCR Reaction Mix (Sigma Aldrich, USA) was used. RSV N, M and NS1 genes were amplified using specific primers (sequence available upon request) that amplify both viral and corresponding antigenome sequences, under following thermal conditions: denaturation at 94 °C for 1 min, annealing at 55 °C for 1 min and extension at 72 °C for 1 min for 35 cycles and a final extension step at 72 °C for 7 min. The PCR product were subjected to agarose gel (1.5%) electrophoresis and analyzed under UV trans-illuminator. To determine whether exosomes would carry RSV specific genome and/or antigenome sequences, we used primers targeting intergenic regions of RSV genome as previously described^86^.

To validate the upregulation of miRNA expression in RSV exosomes, miRNA expression was examined using miScript II cDNA synthesis reagents, miRNA primers and miScript SYBR Green PCR system (all from Qiagen Inc.), following manufactures instructions.

### Next Generation small RNA sequencing

Next generation sequencing libraries were generated with the TailorMix Micro RNA Sample Preparation kit version 2 following manufacturer’s instructions (SeqMatic LLC, USA). Briefly, after RNA isolation, 3′-adapter was ligated to the RNA sample and excess 3′-adapters were removed subsequently. 5′-adapter was then ligated to the 3′-adapter ligated samples, followed by first strand cDNA synthesis. cDNA library was amplified and barcoded via enrichment PCR. Final RNA library was size-selected on an 8% TBE polyacrylamide gel and purified, quality checked with Bio-analyzer. Sequencing was performed on the Illumina HiSeq. 2500 platform at SR50 (Illumina Inc., USA).

### Sequencing data analysis

Analysis of sequencing data was done using Exosome small RNA-seq Analysis Kit 1.0 pipeline (Maverix Biomics, USA). Briefly, the raw sequencing reads were subjected to FastQC analysis to determine the quality of the data and make sure there were no sequencing issues and contaminants. After mapping the spike-in DNA with Bowtie2, the adapter sequences, primers and reads with quality score below 28 were trimmed using fastq-mcf of ea-utils^87^ and PRINSEQ^88^. Reads with a remaining length of less than 16 bp after trimming were discarded. Once the data preprocessing step removed N’s, trimmed sequencing adaptors, filtered reads for quality and length, FastQC was re-run to analyze trimmed reads to allow a before and after comparison. The improved pseudo single-end reads formed by merging the post-trimmed paired-end reads using SeqPrep^89^ were mapped to the human genome (GRCh37/hg19) using Bowtie^90^. Read coverage on forward and reverse strands for genome browser^91^ visualization was computed using SAMtools^92^ BEDtools^93^, and UCSC Genome Browser utilities^91^. Raw read counts were calculated for known gene categories including ncRNAs, antisense transcripts, coding and intronic regions of mRNAs, and repeats. Annotations of known genes were retrieved from miRBase release 20^94^, NCBI RefSeq^95^, Human lincRNA Catalog^96^, and UCSC Genome Browser^91^. The final steps of the analysis were abundance determination and differential expression analysis. Raw read counts were normalized across all samples and then used for pairwise differential expression analysis using DEseq^97^. Significant differentially expressed genes were determined by adjusted P-value with a threshold of 0.05. Log_2_ (fold change) between samples were hierarchically clustered using Pearson correlation. The original raw data (read counts) for all identified categories are presented in a supplementary file.

### Predicted targets of miRNA and functional analysis

To characterize potential targets of miRNAs identified in exosomes, we integrated the lists of microRNAs with target predictions available from the TargetScan database. We used version 7.1 of Targetscan, containing 1,387,426 human miRNA-target interactions. Candidate target genes of identified miRNAs were called whenever the gene was paired with the miRNA (or its variant) in the “default predictions” table of Targetscan. We have thus identified 4,816 potential targets of miRNAs upregulated in the exosomes following RSV infection, 925 potential targets of miRNAs downregulated in exosomes (see Table 2), and 2,122 targets of mRNAs highly abundant in exosomes (see Table 3).

We characterized the most prevalent functions of the predicted target genes of the identified miRNAs using the Gene Ontology (GO) functional classification. The GO analysis has been performed using the DAVID 6.8 database. For each group of predicted target genes 1: Upregulated in exosomes following RSV infection, 2: Downregulated in Exosomes following RSV infection, 3: Targets of highly abundant miRNAs listed in Table 3. Because of the high number of genes identified as potential targets of upregulated miRNAs, we limited the set of genes to targets of at least two such miRNAs (2752 genes). The relative frequencies and statistical significance of enrichments were calculated against the entire human genome used as background. The results are presented in supplementary files, with a p-value threshold of detection set to 0.01.

### Monocyte and A549 cell stimulation

PBMC were isolated using Ficoll Hypaque (GE Healthcare, USA). Monocyte isolations were done using EasySep human monocyte isolation kit without CD16 depletion as per the manufacturer’s instructions (Stemcell Technologies, Canada). The viability of cells after isolation was ≥96%, and the purity was >92%. Monocytes or A549 cells were left untreated or treated with 10 μg of exosomes for 24 h and supernatant was subjected to cytokine and chemokine measurements using a custom 8-plex Bioplex assay (BioRad, USA) containing eight targets, namely interleukin-6 (IL-6), interleukin-8 (IL-8), interleukin- 9 (IL-9), Monocyte Chemoattractant Protein-1 (MCP-1), Macrophage Inflammatory Protein-1 alpha (MIP-1α), Macrophage Inflammatory Protein-1 beta (MIP-1β), IFN-gamma-inducible protein-10 (IP-10), and the chemokine CCL5/regulated on activation, normal T cell expressed and secreted (RANTES), as per manufacturer’s instructions.

### Statistical analysis

The data presented are representative of experiments that were performed a minimum of three times. The NGS sequencing data shown are average of three independent experiments. The raw read counts were normalized across all samples and then used for pairwise differential expression analysis using DEseq. Significant differentially expressed genes were determined by adjusted P-value with a threshold of 0.05. Log_2_ (fold change) between samples were hierarchically clustered using Pearson correlation. In miRNA validation RT-PCR experiments fold changes in miRNA expression were determined by the 2-ΔΔCt method and represent mean ± SD of 3-4 independent experiments. Data from the cytokine/chemokine release experiment in monocytes and A549 cells were expressed as mean ± SEM of experiments done in triplicate and the significance between samples was determined by statistical test one-way analysis of variance (ANOVA) using GraphPad Prism v4 (GraphPad Software). A P value <0.05 was considered to indicate a statistically significant difference.

### Data availability statement

The datasets generated during and/or analyzed during the current study, and not submitted as supplementary material, are available from the corresponding author on reasonable request.

## Electronic supplementary material

Supplementary Information

Supplementary Dataset 1

Supplementary Dataset 2

Supplementary Dataset 3

Supplementary Dataset 4

Supplementary Dataset 5

Supplementary Dataset 6

Supplementary Dataset 7

Supplementary Dataset 8

Supplementary Dataset 9

Supplementary Dataset 10
