# Supplementary material for: Respiratory Syncytial Virus Infection Changes Cargo Composition of Exosome Released from Airway Epithelial Cells
Source: Sci Rep. 2018 Jan 10;8:387. doi: 10.1038/s41598-017-18672-5 (PMC5762922; doi:10.1038/s41598-017-18672-5)

SUPPLEMENTARY INFORMATION

Respiratory Syncytial Virus Infection Changes Cargo Composition of Exosome Released from Airway Epithelial Cells

Harendra Singh Chahar[1], Tiziana Corsello[1], Andrzej S. Kudlicki[2], Narayana Komaravelli[1], Antonella Casola[1, 3,4*]

[1]University of Texas Medical Branch at Galveston, Department of Pediatrics, Galveston, 77555, USA

[2]University of Texas Medical Branch at Galveston, Department of Biochemistry and Molecular Biology, Galveston, 77555, USA

[3]University of Texas Medical Branch at Galveston, Sealy Center for Vaccine Development, Galveston, 77555, USA

[4]University of Texas Medical Branch at Galveston, Sealy Center for Molecular Medicine, Galveston, 77555, USA

*ancasola@utmb.edu

RESULTS

ST1: Complete list of miRNAs whose expression in exosomes was changed following RSV infection 10
ST2: Predicted target genes of miRNAs upregulated by RSV from Table 2 10
ST3: Predicted target genes of all detected miRNAs from Table 3........ 10
ST4: Predicted target genes of miRNAs downregulated by RSV from Table 2 and 3..... 10
ST5: Complete list of piRNAs whose expression in exosomes was changed following RSV infection 11

Supplementary file legend for Gene Ontology data analysis files:
We have identified the most significantly enriched functional groups in the following categories: Gene Ontology Biological Process (GOTERM_BP_DIRECT), Gene_Ontology Molecular Function (GOTERM_MF_DIRECT), KEGG pathway (KEGG_PATHWAY). The results are shown in nine supplementary Excel spreadsheet files. We have imposed a p-value threshold of 0.01. Due to the large number of upregulated targets, we limited the set of targets of upregulated miRNAs to genes identified two or more times by TargetScan. The columns of the spreadsheets are as follows:
A: Type of functional category (same for all results in a file),
B: Functional category enriched,
C: Number of identified genes with the annotation from column B
D: P-Value for enrichment (uncorrected)
E: Enrichment fold
F, G, H: Significance with Bonferroni, Benjamini and FDR correction, respectively.

SD1. Next generation high-throughput sequencing read counts........ 9
SD2.GOTERM_BP_DIRECT analysis of targets of upregulated miRNA of Table 2 10
SD3.GOTERM_MF_DIRECT analysis of targets of upregulated miRNA of Table 2 10
SD4. KEGG_PATHWAY analysis of targets of upregulated miRNA of Table 2 10
SD5.GOTERM_BP_DIRECT analysis of targets of downregulated miRNA of Table 2 10
SD6.GOTERM_MF_DIRECT analysis of targets of downregulated miRNA of Table 2.10
SD7.KEGG_PATHWAY analysis of targets of downregulated miRNA of Table 2........ 10
SD8.GOTERM_BP_DIRECT analysis of targets of miRNA of Table 3 10
SD9.GOTERM_MF_DIRECT analysis of targets of miRNA of Table 3 10
SD10. KEGG_PATHWAY analysis of targets of miRNA of Table 3 10

		Read Count (Avg)		Log2 Fold
S.No.	miRNA	Mock Exo	RSV Exo	Change
1	hsa-mir-6087	45.59	39800.59	9.7699
2	hsa-let-7e	0.62	468.17	9.5657
3	hsa-miR-182-5p	0.25	43.34	7.4339
4	hsa-miR-181b-5p	0.62	77.21	6.9655
5	hsa-mir-128-1	0.25	14.68	5.8724
6	hsa-miR-19b-3p	0.25	13.24	5.7227
7	hsa-mir-23b	1.14	54.52	5.5854
8	hsa-let-7i	12.76	610.07	5.5789
9	hsa-mir-22	2	79.13	5.3033
10	hsa-miR-24-3p	5.98	229.59	5.263
11	hsa-mir-182	1.39	45.8	5.0462
12	hsa-mir-378a	0.87	28.12	5.0172
13	hsa-mir-99b	1.14	35.93	4.9836
14	hsa-miR-320b	2.62	74.63	4.8312
15	hsa-let-7f-1	22.5	590.86	4.7148
16	hsa-miR-4516	2.97	77.27	4.7001
17	hsa-mir-21	121.4	3155.14	4.6999
18	hsa-mir-30c-2	2.1	42.72	4.3435
19	hsa-miR-423-5p	2.24	44.06	4.2989
20	hsa-miR-320c	1.75	34.03	4.2788
21	hsa-mir-23a	3.41	65.92	4.2744
22	hsa-miR-25-3p	6.01	113.35	4.2366
23	hsa-mir-25	6.01	113.35	4.2366
24	hsa-mir-423	5.13	85.27	4.0558
25	hsa-mir-324	0.62	10.25	4.0522
26	hsa-miR-98-5p	2	33.18	4.0495
27	hsa-miR-224-5p	1.14	15.93	3.8104
28	hsa-mir-92b	2.25	29.38	3.704
29	hsa-mir-10a	1.64	19.97	3.6088
30	hsa-mir-221	36.28	433.51	3.5787
31	hsa-miR-320d	1.14	13.17	3.5361
32	hsa-mir-34a	1.75	20.13	3.5214
33	hsa-let-7a-5p	126.89	1387.4	3.4508
34	hsa-miR-16-5p	15.01	150.62	3.3266
35	hsa-mir-106b	3.41	32.84	3.2689
36	hsa-mir-4449	25.37	214.29	3.0785
37	hsa-mir-103a-1	17.27	143.31	3.0528

38	hsa-mir-320a	72.59	504.05	2.7957
39	hsa-mir-27b	59.5	382.68	2.6851
40	hsa-let-7d	9.27	56.12	2.5971
41	hsa-mir-15b	1.85	10.13	2.451
42	hsa-mir-140	2.52	12.94	2.3598
43	hsa-let-7g	41.88	195.77	2.2248
44	hsa-miR-107	8.38	35.46	2.0807
45	hsa-mir-92a-1	56.61	238.69	2.0761
46	hsa-mir-192	30.42	128.24	2.0757
47	hsa-miR-92a-3p	53.72	225.96	2.0725
48	hsa-mir-125a	5.51	22.94	2.0563
49	hsa-mir-27a	28.68	114.13	1.9928
50	hsa-miR-29a-3p	14.88	53.23	1.839
51	hsa-mir-29a	14.88	53.23	1.839
52	hsa-mir-17	4.63	12.56	1.4416
53	hsa-mir-30d	25.98	69.77	1.4251
54	hsa-miR-17-5p	4.63	11.19	1.275
55	hsa-let-7b	301.51	717.56	1.2509
56	hsa-mir-93	34.52	79.26	1.1992
57	hsa-mir-223	21	0.17	-6.9115
58	hsa-mir-2964a	62.14	0.57	-6.7673
59	hsa-mir-205	18.17	0.29	-5.9932
60	hsa-mir-143	65.39	1.41	-5.5322
61	hsa-miR-3168	222.71	4.82	-5.5304
62	hsa-miR-146a-5p	15.8	0.46	-5.1029
63	hsa-miR-10b-5p	56.07	4	-3.809
64	hsa-mir-328	21.77	1.83	-3.5758
65	hsa-miR-1246	237.74	23.03	-3.3677
66	hsa-miR-185-5p	17.3	6.91	-1.3244
		**Read Count (Avg)**		
**S.No.**	**miRNA**	**Mock Exo**	**RSV Exo**	
1	hsa-mir-31	0	529.15	N/A
2	hsa-mir-3687	0	114.57	N/A
3	hsa-mir-222	0	80.93	N/A
4	hsa-mir-151a	0	66.1	N/A
5	hsa-miR-4449	0	65.29	N/A
6	hsa-mir-663a	0	56.03	N/A
7	hsa-mir-196a-2	0	53.41	N/A
8	hsa-mir-4508	0	37.12	N/A
9	hsa-mir-1248	0	34.64	N/A

10	hsa-miR-4497	0	28.19	N/A
11	hsa-mir-1273f	0	28.08	N/A
12	hsa-mir-28	0	26.67	N/A
13	hsa-mir-191	0	20.44	N/A
14	hsa-miR-3648	0	17.54	N/A
15	hsa-miR-194-5p	0	17.14	N/A
16	hsa-miR-128	0	14.3	N/A
17	hsa-mir-1307	0	13.91	N/A
18	hsa-mir-744	0	13.07	N/A
19	hsa-miR-148b-3p	0	12.31	N/A
20	hsa-miR-1307-3p	0	12.02	N/A
21	hsa-mir-663b	0	11.33	N/A
22	hsa-miR-152	0	11.19	N/A
23	hsa-mir-421	0	10.99	N/A
24	hsa-mir-7-1	0	10.43	N/A
25	hsa-mir-486	0	10.26	N/A
26	hsa-miR-1321	50.5	0	N/A
27	hsa-mir-204	34.07	0	N/A
28	hsa-mir-637	22.18	0	N/A
29	hsa-mir-4689	19.39	0	N/A
30	hsa-mir-144	15.38	0	N/A
31	hsa-miR-4269	11.12	0	N/A
32	hsa-mir-885	10.5	0	N/A
33	hsa-mir-4795	10.5	0	N/A
34	hsa-mir-718	10.29	0	N/A

Supplementary Table 1. Complete list of miRNAs whose expression in exosomes was changed following RSV infection.

Supplementary Table 2. Predicted target genes of miRNAs upregulated by RSV from Table 2.

Target Gene	Number of miRNAs
TRIM71	20
FIGN	17
ONECUT2	16
HIC2	15
IGF2BP1	15
AGO3	14
CHIC1	14
HMGA2	14
AGO2	13
SESN3	13
CLCN5	12
ANKRD52	11
CCND2	11
FOXP2	11
LIN28B	11
NR6A1	11
ONECUT3	11
QKI	11
ACVR2B	10
ARID3B	10
CNNM2	10
FZD3	10
GABRB1	10
LPP	10
PBX3	10
POU2F1	10
TAOK1	10
ZNF286B	10
ZNF704	10
ABHD13	9
BNC2	9
CPD	9
DCUN1D3	9
FRS2	9
IGDCC3	9

INO80D	9
KDM5A	9
KIAA2022	9
MTDH	9
NUFIP2	9
S1PR1	9
SKIL	9
STOX2	9
ZBTB20	9
ZNF652	9
AAK1	8
ATXN1	8
CPEB4	8
DCUN1D1	8
FAM222B	8
FNDC3B	8
GXYLT1	8
IGF1R	8
KCNC3	8
KCTD16	8
KLF7	8
MBD2	8
MEX3A	8
PBX2	8
PLXNC1	8
PURB	8
USP44	8
YAF2	8
YOD1	8
ZBTB16	8
AGO4	7
AKT3	7
AL590483.1	7
ARID3A	7
ARPP19	7
ATXN3	7

BEND4	7
C14orf28	7
CBX5	7
CDC73	7
CLOCK	7
CREBRF	7
CSRNP3	7
CYLD	7
DYRK2	7
EGR3	7
EOGT	7
GAN	7
GIGYF1	7
IRF6	7
ITGB3	7
ITGB8	7
KCMF1	7
LCOR	7
LRIG2	7
MAPK1	7
MAPK1IP1L	7
MTMR12	7
NOVA2	7
ONECUT1	7
PAPPA	7
PBX1	7
PCDHA10	7
PCDHA11	7
PCDHA12	7
PCDHA13	7
PCDHA1	7
PCDHA2	7
PCDHA3	7
PCDHA4	7
PCDHA5	7
PCDHA6	7
PCDHA7	7
PCDHA8	7
PCDHA9	7
PCDHAC1	7
PCDHAC2	7
PGM2L1	7
PTPRD	7
RAB3GAP2	7
RIT1	7
RNF152	7
SALL4	7
SH2B3	7
SNX30	7
SOX6	7
SREK1	7
ST3GAL1	7
TET3	7
TGFBR1	7
THRA	7
TNPO1	7
TSC22D2	7
WDFY2	7
ZBTB10	7
ZBTB37	7
ZNF24	7
ZNF280B	7
ADCY9	6
ATXN1L	6
BMP2K	6
BRWD1	6
BRWD3	6
BZW1	6
C1GALT1	6
C20orf194	6
CELF1	6
CFL2	6
CPEB1	6
DCAF8	6
DDX3X	6
DICER1	6

DLC1	6
DLGAP4	6
DMD	6
DNAJA2	6
DNAL1	6
DR1	6
DTNA	6
ETNK1	6
EXOC5	6
FNDC3A	6
GNAQ	6
GPCPD1	6
GRIN2A	6
HIF1AN	6
HS2ST1	6
ITSN1	6
KIAA1244	6
KIAA1958	6
KLHL15	6
KPNA4	6
LGALSL	6
LPGAT1	6
LRRC40	6
MEF2C	6
MEIS2	6
MSI2	6
NFIA	6
NKAP	6
NLN	6
OSBPL3	6
PIK3CA	6
PLXDC2	6
PLXNA4	6
PPARGC1B	6
PRR14L	6
PTAFR	6
PTAR1	6
PTEN	6
PXDN	6
RAP2C	6
RASA1	6
RGS17	6
RIMKLA	6
RIMS4	6
RORA	6
SDK1	6
SFMBT1	6
SLC16A10	6
SOX12	6
SOX4	6
STARD13	6
SYNCRIP	6
TGFBR2	6
TMEM178B	6
TMEM64	6
TNIK	6
TNRC6B	6
TOR1AIP2	6
TSC1	6
UBN2	6
WWC2	6
XKR4	6
ZBTB4	6
ZNF512B	6
ZNF697	6
ABL2	5
ACAP2	5
ACVR2A	5
AGO1	5
ANKFY1	5
AP3M1	5
APBA1	5
ARL5A	5
ATP11A	5
ATXN7L3	5
B3GALT1	5

BEND3	5
BPTF	5
C19orf26	5
CACNA1E	5
CACNB4	5
CALN1	5
CBFA2T3	5
CCDC144A	5
CDK13	5
CDK6	5
CECR6	5
CNKSR3	5
CNOT6L	5
CPEB3	5
CTD-2140B24.4	5
CTDSPL2	5
CYB561D1	5
DCBLD2	5
DDI2	5
DDX3Y	5
DUSP16	5
ERO1L	5
EVX2	5
FOXP1	5
GALNT1	5
GATA6	5
GDF5OS	5
GMFB	5
GPBP1	5
GPR61	5
GRAMD1B	5
GREB1L	5
HAS2	5
HCFC2	5
HOXA1	5
IGF2BP2	5
IGF2BP3	5
KCNJ6	5
KIAA0247	5
KIAA1549	5
KLF13	5
KLF8	5
KLHL3	5
KLHL36	5
KMT2E	5
LIN28A	5
LMLN	5
LPHN3	5
MAP1B	5
MAP3K3	5
MGLL	5
MIB1	5
MIER1	5
MITF	5
MKL2	5
MTF2	5
MXI1	5
MYCN	5
N4BP1	5
NAA20	5
NABP1	5
NAP1L1	5
NEDD4L	5
NEK9	5
NLK	5
NRAS	5
NRK	5
NXT2	5
OTUD7B	5
PARM1	5
PARP8	5
PCGF3	5
PDK3	5
PDZD8	5
PHC1	5
PHIP	5

PLAG1	5
PPP1R15B	5
PRDM1	5
PRKAR2A	5
PRLR	5
PTCHD1	5
PYGO2	5
RAB30	5
RAB8B	5
RAD23B	5
REEP1	5
RGS9BP	5
RICTOR	5
RNF165	5
RNF38	5
RSF1	5
RUNX1T1	5
SAMD4A	5
SCN1A	5
SEMA4G	5
SENP2	5
SH3KBP1	5
SKIDA1	5
SLAIN2	5
SLC10A7	5
SLC30A7	5
SLC6A6	5
SLC7A14	5
SNN	5
SRSF7	5
STIM2	5
STK24	5
STRN	5
SYT11	5
TANC2	5
TFAP2B	5
TMEM151B	5
TMEM246	5

TRIM41	5
USP15	5
VANGL2	5
VCPIP1	5
VGLL3	5
WIPI2	5
WNT9B	5
XIAP	5
XYLT1	5
ZBTB7A	5
ZMYND11	5
ZNF200	5
ZNF268	5
ZNF292	5
ZNF516	5
ZNF644	5
ZNF710	5
ZNF772	5
ZNF791	5
AC140061.12	4
ACER2	4
ACER3	4
ACSL6	4
ADAMTS15	4
ADAMTS5	4
ADCY6	4
ADRB1	4
AEN	4
AFF1	4
AIFM1	4
AKAP2	4
AKAP5	4
AMMECR1L	4
APBB2	4
AQR	4
ARID5B	4
ARL5B	4
ASXL2	4

ATAD2B	4
ATF7IP2	4
ATG14	4
ATG16L1	4
ATP2A2	4
ATP2B2	4
ATXN7	4
AZIN1	4
B3GNT7	4
BACH1	4
BAHD1	4
BCAP29	4
BCAT1	4
BCL2L11	4
BDP1	4
BLCAP	4
BLOC1S6	4
BMPR1A	4
BMPR2	4
BSN	4
BTBD3	4
BVES	4
CACNA1C	4
CADM2	4
CALM1	4
CAMSAP2	4
CBL	4
CBX7	4
CCNJ	4
CCNT2	4
CCR7	4
CDC34	4
CDH20	4
CDV3	4
CECR2	4
CHD7	4
CHMP7	4
CHST3	4
CHSY3	4
CLASP1	4
CNKSR2	4
CNOT2	4
CNOT6	4
COL27A1	4
COPS2	4
CPEB2	4
CREBL2	4
CRK	4
CTB-54O9.9	4
CTPS1	4
CUL5	4
CYTH3	4
DAB1	4
DCP1A	4
DCX	4
DENND6A	4
DIAPH2	4
DLGAP2	4
DNAJB14	4
DPYSL3	4
DST	4
DTX2	4
DTX4	4
DVL3	4
DYNC1LI2	4
E2F2	4
EEA1	4
EFNB2	4
ELL2	4
ELMSAN1	4
EMB	4
EMC7	4
ENAH	4
EPC1	4
EPHA4	4
EPN2	4

ETV3	4
EVI5	4
EYA3	4
FAM117B	4
FAM118A	4
FAM160B1	4
FAM168A	4
FAM179B	4
FAM208A	4
FAM43A	4
FAM63B	4
FAXC	4
FMR1	4
FNIP2	4
FRMD4B	4
FSD2	4
FUT9	4
FXR1	4
GAS7	4
GATSL2	4
GBX2	4
GCNT4	4
GID4	4
GJC1	4
GK5	4
GNAL	4
GNS	4
GRIN2B	4
HECTD2	4
HELZ	4
HHIP	4
HIAT1	4
HIC1	4
HIF3A	4
HLF	4
HNRNPA1	4
HOXA9	4
HOXD1	4

IGF1	4
IKZF1	4
IL1RAPL1	4
IL6R	4
IMPG2	4
IPO5	4
ITGA1	4
JAZF1	4
JHDM1D	4
KCND3	4
KLF6	4
KMT2A	4
KPNA1	4
KRAS	4
LBR	4
LEPROTL1	4
LHX9	4
LIMCH1	4
LIMD2	4
LMTK2	4
LPHN1	4
LPHN2	4
MAP3K2	4
MAPK8	4
MARCH9	4
MBLAC2	4
MBNL2	4
MBNL3	4
MBTPS2	4
MDM4	4
MED12L	4
MED28	4
MEF2A	4
MEF2D	4
MFAP3L	4
MIER3	4
MLEC	4
MMP16	4

MOB3B	4
MRPS25	4
MSANTD4	4
MXD1	4
MYRIP	4
MZT1	4
NAA30	4
NACC2	4
NCKAP1	4
NFIB	4
NFIC	4
NIPAL4	4
NOVA1	4
NRP1	4
NSUN3	4
NUP37	4
OTUD3	4
PACS2	4
PAK1	4
PALM2	4
PALM2-AKAP2	4
PAN3	4
PAPD5	4
PAQR3	4
PARD6B	4
PCDH19	4
PCGF5	4
PDE12	4
PDGFRA	4
PGRMC1	4
PHF8	4
PI4K2A	4
PKN2	4
PLEKHA3	4
PLEKHA8	4
PLEKHO1	4
PPM1L	4
PPP1R12B	4

PPP1R16B	4
PRKAA2	4
PRKAB2	4
PRKACB	4
PROX1	4
PRSS22	4
PTPRG	4
RAB11FIP4	4
RAB27A	4
RAB2A	4
RALGPS1	4
RANBP2	4
RAP1A	4
RAP1B	4
RAPGEF2	4
RASL10B	4
RGP1	4
RIMS3	4
RNF150	4
RNF20	4
RNF217	4
RNF44	4
RP1-170O19.20	4
RRAGD	4
RRP1B	4
SAMD12	4
SAR1B	4
SCN8A	4
SCUBE3	4
SECISBP2L	4
SEMA4C	4
SENP5	4
SESTD1	4
SLC25A12	4
SLC26A9	4
SLC30A1	4
SLC5A3	4
SLC8A1	4

SMARCAD1	4
SMARCC1	4
SMC1A	4
SMCR8	4
SMURF2	4
SNTB2	4
SNX17	4
SOCS4	4
SOX5	4
SP1	4
SPATA13	4
SPATA2	4
SPATS2L	4
SPIRE1	4
SREK1IP1	4
SYT1	4
TBL1X	4
TBX18	4
TET2	4
TGFBR3	4
THBS1	4
TIA1	4
TMCC1	4
TMEM106B	4
TMEM2	4
TMEM255A	4
TMEM41A	4
TNKS	4
TNRC6C	4
TP53	4
TP53INP1	4
TPD52L2	4
TRABD2B	4
TRHDE	4
TRIM67	4
TRPM6	4
TTLL4	4
UGCG	4
UGGT1	4
UNKL	4
USP46	4
VEZT	4
VKORC1L1	4
VLDLR	4
VSTM5	4
WARS2	4
WASL	4
WDTC1	4
WNK3	4
YIPF6	4
YY1	4
ZADH2	4
ZC3H7B	4
ZFP36L1	4
ZFP36L2	4
ZIC3	4
ZNF207	4
ZNF256	4
ZNF275	4
ZNF281	4
ZNF788	4
ABCB9	3
ABHD17C	3
AC010441.1	3
ACVR1B	3
ACVR1C	3
ADAM10	3
ADAMTS1	3
ADAMTS14	3
ADAMTS18	3
ADARB1	3
ADD3	3
ADIPOR2	3
AGFG1	3
AGMAT	3
AGPAT3	3

AGPAT9	3
AGPS	3
AK3	3
ALDH6A1	3
ALG9	3
ANGPTL2	3
ANKRD13C	3
ANKRD33B	3
ANXA11	3
ARF3	3
ARFGEF2	3
ARFIP1	3
ARHGAP44	3
ARHGEF12	3
ARID2	3
ARMC1	3
ARMC8	3
ARRDC3	3
ARRDC4	3
ASAP1	3
ASCC1	3
ASXL3	3
ATF7IP	3
ATG7	3
ATL3	3
ATP2B1	3
ATRX	3
ATXN7L1	3
ATXN7L3B	3
AVL9	3
B3GALNT2	3
B3GNT1	3
BACE2	3
BCAS3	3
BCKDK	3
BCL2	3
BCL7A	3
BOD1L1	3

BSND	3
BTF3L4	3
BTG2	3
C11orf30	3
C11orf57	3
C16orf52	3
C17orf85	3
C1orf95	3
C20orf112	3
C21orf91	3
C2CD5	3
C2orf69	3
C6orf211	3
C7orf41	3
C8orf58	3
CADM1	3
CALD1	3
CAMSAP1	3
CAPRIN1	3
CAPRIN2	3
CBX2	3
CCDC117	3
CCDC6	3
CCND1	3
CCNY	3
CD164	3
CD2AP	3
CDC42BPA	3
CDC42SE1	3
CDYL	3
CELSR3	3
CEP135	3
CEP350	3
CEP41	3
CEP85L	3
CHD9	3
CHRM3	3
CHST1	3

CHST7	3
CLIP1	3
CNBP	3
CNOT7	3
COL1A2	3
CPOX	3
CREB1	3
CREB3L2	3
CREBZF	3
CRKL	3
CRY2	3
CSNK1G1	3
CSNK2A1	3
CTDNEP1	3
CTIF	3
DAG1	3
DAGLA	3
DAZAP1	3
DBN1	3
DBNL	3
DCAF12	3
DDN	3
DENND5B	3
DERL1	3
DESI2	3
DHX30	3
DHX40	3
DKK3	3
DLG2	3
DLX1	3
DMXL2	3
DNAH10OS	3
DNAH3	3
DNAJB9	3
DNAJC16	3
DNAJC3	3
DOCK3	3
DOCK4	3

DUSP1	3
DUSP22	3
DUSP7	3
DYRK1A	3
E2F5	3
EBF3	3
EDNRB	3
EFNA5	3
EIF3J	3
EIF4G2	3
EIF5	3
EIF5A2	3
ELL	3
ENTPD6	3
EPHA3	3
ERBB4	3
EREG	3
ESR1	3
ESRRG	3
ETNK2	3
ETV1	3
EVX1	3
EXO1	3
EXT1	3
EYA4	3
FAM104A	3
FAM126B	3
FAM134B	3
FAM135A	3
FAM155A	3
FAM160B2	3
FAM188A	3
FAM46C	3
FAM49B	3
FAM73A	3
FAM78A	3
FAM83G	3
FARP1	3

FAS	3
FASLG	3
FASTK	3
FAT3	3
FBXL19	3
FBXO28	3
FBXO32	3
FBXO33	3
FBXO42	3
FBXO45	3
FBXW11	3
FEM1C	3
FGF11	3
FKBP5	3
FLNB	3
FLNC	3
FMNL3	3
FOSL2	3
FOXQ1	3
FRAS1	3
FREM2	3
FRMD5	3
G3BP1	3
GAB2	3
GABBR2	3
GABRA1	3
GABRA4	3
GALNT2	3
GALNT4	3
GAPVD1	3
GAREM	3
GDF6	3
GDPD1	3
GFPT1	3
GHITM	3
GHR	3
GJA9	3
GLIS3	3

GLRA3	3
GNB4	3
GOLGA1	3
GOT2	3
GPC4	3
GPD1L	3
GPR137C	3
GRIK2	3
GRIK3	3
GRM5	3
GSKIP	3
GSPT1	3
HAND2	3
HAPLN1	3
HAS3	3
HBEGF	3
HDLBP	3
HECA	3
HECW2	3
HEY2	3
HIF1A	3
HIP1	3
HLTF	3
HNRNPF	3
HOMER1	3
HOXB4	3
HS6ST1	3
IDH2	3
IFFO2	3
IGSF3	3
ING5	3
INO80	3
INPP5A	3
INSIG1	3
IPMK	3
IPO9	3
IRS2	3
JMJD1C	3

KAT6B	3
KAT7	3
KATNAL1	3
KATNBL1	3
KCNA4	3
KCNC4	3
KCNJ15	3
KCNJ2	3
KCNK10	3
KCNK2	3
KCNK6	3
KCNQ5	3
KCNV1	3
KCTD10	3
KCTD21	3
KHNYN	3
KIAA0226	3
KIAA1211	3
KIAA1239	3
KIAA1324	3
KIAA1549L	3
KIAA1598	3
KIF21B	3
KIF2A	3
KIF3B	3
KITLG	3
KLHDC10	3
KLHL13	3
KLHL14	3
KLHL31	3
KLHL42	3
KPNA3	3
KPNB1	3
KREMEN1	3
LAMC1	3
LBH	3
LIMD1	3
LIMK2	3
LIPT2	3
LMO3	3
LONRF3	3
LPPR1	3
LRIG3	3
LRP12	3
LRP6	3
LRRC4	3
LRRC8B	3
LRRFIP1	3
LUZP1	3
LYVE1	3
LZIC	3
MAP1LC3B	3
MAP3K1	3
MAP3K9	3
MAP4K3	3
MAP4K4	3
MAPK6	3
MARK4	3
MASTL	3
MBD6	3
MCC	3
MED16	3
MED8	3
MEX3B	3
MGA	3
MGAT2	3
MGAT4A	3
MLLT10	3
MLXIP	3
MN1	3
MOB3C	3
MON2	3
MORC3	3
MTMR3	3
MTUS1	3
MYCBP	3

MYO1D	3
N4BP3	3
NAT8L	3
NAV3	3
NCALD	3
NCOA1	3
NDST1	3
NEFM	3
NEGR1	3
NEUROD1	3
NFAT5	3
NFATC2	3
NFIX	3
NGF	3
NHLRC3	3
NIPA1	3
NMT2	3
NPAS2	3
NPEPL1	3
NPTXR	3
NR2C2	3
NR3C1	3
NRN1	3
NSD1	3
NTN1	3
NUAK1	3
NUDT21	3
NUMBL	3
NUP155	3
NUS1	3
OGT	3
ORC2	3
PAK7	3
PAWR	3
PCDH10	3
PCDH1	3
PCSK1	3
PCYOX1	3
PDCD4	3
PDE10A	3
PDE3B	3
PDE4D	3
PDE5A	3
PDP2	3
PEG10	3
PFKM	3
PGR	3
PHACTR2	3
PHACTR4	3
PHF13	3
PHLPP2	3
PHOX2B	3
PHTF2	3
PI15	3
PI4K2B	3
PIK3CB	3
PIK3IP1	3
PIK3R1	3
PIK3R3	3
PKDCC	3
PKIA	3
PLCG1	3
PLCL2	3
PLCXD3	3
PLK3	3
PMEPA1	3
POC1B-GALNT4	3
POLR3D	3
PPM1A	3
PPP1CB	3
PPP1R11	3
PPP1R12A	3
PPP1R3E	3
PPP2R2C	3
PPP2R5E	3
PPP3CA	3

PPP4R2	3
PPTC7	3
PRICKLE2	3
PRKG1	3
PRRX1	3
PRUNE2	3
PTGER3	3
RAB11A	3
RAB15	3
RAB18	3
RAB22A	3
RAB3C	3
RAB5B	3
RAC1	3
RAD51	3
RAD51L3-RFFL	3
RAI2	3
RALB	3
RAP2B	3
RAPGEFL1	3
RARG	3
RASAL2	3
RASSF8	3
RAVER2	3
RBFOX2	3
RBMS1	3
RBMS3	3
RCN2	3
RDX	3
RFFL	3
RGS5	3
RHPN2	3
RLIM	3
RNF11	3
RNF145	3
RORB	3
RP11-766F14.2	3
RPRD2	3
RPS24	3
RPS6KA3	3
RPS6KA6	3
RPS6KB1	3
RRP8	3
RSPO2	3
RSU1	3
RTKN2	3
RUNX1	3
RXRA	3
SALL3	3
SAP30L	3
SATB2	3
SBK1	3
SBNO1	3
SCAI	3
SCD	3
SCML2	3
SCN3A	3
SCN4B	3
SCOC	3
SCRT2	3
SEC63	3
SEMA4F	3
SERINC3	3
SERTAD2	3
SF3B3	3
SGCB	3
SGMS2	3
SH3RF3	3
SHC3	3
SKAP2	3
SLC16A7	3
SLC16A9	3
SLC22A23	3
SLC22A3	3
SLC25A24	3
SLC25A4	3

SLC25A51	3
SLC35F1	3
SLC48A1	3
SLC5A6	3
SLC6A8	3
SLC7A6OS	3
SLITRK3	3
SMAD2	3
SMARCD2	3
SMARCE1	3
SMG1	3
SMG7	3
SMIM13	3
SMIM3	3
SNAP23	3
SNX1	3
SOCS1	3
SOCS7	3
SOST	3
SOWAHA	3
SPIN1	3
SPRED3	3
SPRY4	3
SPTLC2	3
SPTSSA	3
SRGAP1	3
SRGAP3	3
SRPK2	3
SRPX2	3
SS18L1	3
SSBP2	3
ST8SIA4	3
STAT3	3
STX17	3
STXBP5	3
SULF1	3
SV2C	3
SYNGR2	3

SYNM	3
SYT2	3
TAF4	3
TAF9B	3
TBR1	3
TCERG1	3
TECPR2	3
TFCP2L1	3
THSD7A	3
TIPARP	3
TLK2	3
TMEM110	3
TMEM135	3
TMEM136	3
TMEM167B	3
TMEM47	3
TMEM50B	3
TMOD2	3
TNFAIP3	3
TNFRSF1B	3
TNFSF9	3
TNKS2	3
TNS3	3
TPD52	3
TRANK1	3
TRIM13	3
TRIM8	3
TRIOBP	3
TRPC4AP	3
TRPM3	3
TSHZ3	3
TSPAN14	3
TSPAN18	3
TUSC3	3
TXLNA	3
TXLNG	3
UHRF2	3
ULK2	3

UNC5A	3
USP12	3
USP24	3
USP47	3
USP6	3
VAMP3	3
VANGL1	3
VAT1L	3
VPS26B	3
VPS37B	3
VSTM4	3
WAC	3
WAPAL	3
WDR1	3
WDR37	3
WHAMM	3
WIPF2	3
WNT1	3
WNT9A	3
WRNIP1	3
XPR1	3
XRN1	3
YBX2	3
YIPF5	3
YTHDF3	3
YWHAE	3
YWHAZ	3
ZBTB18	3
ZBTB39	3
ZBTB41	3
ZBTB8A	3
ZBTB8B	3
ZC3H10	3
ZCCHC11	3
ZCCHC14	3
ZCCHC3	3
ZDHHC21	3
ZDHHC3	3

ZFAND5	3
ZFP91	3
ZFYVE26	3
ZMAT3	3
ZMYM4	3
ZNF217	3
ZNF451	3
ZNF594	3
ZNF654	3
ZNF664	3
ZNF70	3
ZNF740	3
ZNRF2	3
ZPLD1	3
A1CF	2
AASS	2
ABCC10	2
ABCC5	2
ABCG1	2
ABI2	2
AC008443.1	2
AC012123.1	2
AC012215.1	2
AC023632.1	2
AC068987.1	2
ACADSB	2
ACAN	2
ACAP3	2
ACBD5	2
ACPP	2
ACSBG1	2
ACSS1	2
ADAM11	2
ADAM12	2
ADAM15	2
ADAM19	2
ADAMTS8	2
ADAT2	2

ADCYAP1	2
ADD2	2
ADK	2
ADO	2
ADRB2	2
ADRB3	2
ADRBK2	2
AEBP2	2
AFF2	2
AFTPH	2
AGAP1	2
AGPAT6	2
AHCTF1	2
AIF1L	2
AIM1	2
AJUBA	2
AK4	2
AKAP6	2
AKT2	2
AL020996.1	2
AL161915.1	2
ALKBH1	2
ALKBH5	2
ALPK3	2
AMER3	2
AMOT	2
AMOTL2	2
AMT	2
ANK3	2
ANKH	2
ANKIB1	2
ANKRD13A	2
ANKRD28	2
ANKRD44	2
ANKRD46	2
AP1S1	2
AP5M1	2
APBB3	2

APC2	2
APPBP2	2
APPL1	2
AREL1	2
ARF4	2
ARFGEF1	2
ARG2	2
ARHGAP20	2
ARHGAP28	2
ARHGEF15	2
ARHGEF3	2
ARHGEF38	2
ARHGEF39	2
ARHGEF7	2
ARL4D	2
ARL6IP6	2
ARL8B	2
ARMCX2	2
ARSJ	2
ASAH2	2
ASAH2B	2
ASCC2	2
ASH2L	2
ASS1	2
ASTN1	2
ATF2	2
ATG4B	2
ATP1B1	2
ATP2B3	2
ATP2B4	2
ATP6V1C1	2
ATP8A1	2
ATP8B2	2
ATP8B4	2
ATPAF1	2
B3GALT5	2
B3GAT1	2
B3GAT3	2

BACE1	2
BACH2	2
BAG4	2
BAI3	2
BAK1	2
BANP	2
BAZ2A	2
BBC3	2
BBX	2
BCL10	2
BCL2L1	2
BCL2L12	2
BCLAF1	2
BEGAIN	2
BHLHE22	2
BICD1	2
BIN3	2
BIRC6	2
BLOC1S5	2
BMF	2
BMI1	2
BMP2	2
BMP3	2
BMP5	2
BMP6	2
BOD1L2	2
BRD1	2
BRPF3	2
BTAF1	2
BTBD7	2
BTBD9	2
BTG1	2
BZW2	2
C11orf84	2
C12orf36	2
C15orf39	2
C15orf41	2
C15orf61	2

C16orf70	2
C19orf47	2
C1orf147	2
C2orf88	2
C3orf14	2
C3orf52	2
C5orf30	2
C5orf51	2
C6orf203	2
C7orf55-LUC7L2	2
C7orf60	2
C8orf44-SGK3	2
C9orf40	2
C9orf41	2
CA12	2
CAB39	2
CACFD1	2
CACNA1I	2
CACNA2D1	2
CACNA2D2	2
CACNG2	2
CACNG4	2
CACUL1	2
CADM3	2
CALCR	2
CALM3	2
CALU	2
CAMK2N1	2
CAND1	2
CANT1	2
CAPN3	2
CAPN7	2
CARD10	2
CASK	2
CASKIN1	2
CASP2	2
CASP3	2
CASP5	2

CASP9	2
CBFA2T2	2
CBLB	2
CBX6	2
CCDC64B	2
CCDC71L	2
CCDC80	2
CCDC88A	2
CCDC92	2
CCDC93	2
CCL3	2
CCL3L1	2
CCL3L3	2
CCL7	2
CCNF	2
CCNJL	2
CCSAP	2
CCSER1	2
CCSER2	2
CD200R1	2
CD274	2
CD86	2
CD97	2
CDC25A	2
CDC27	2
CDCA8	2
CDH2	2
CDH22	2
CDH7	2
CDK14	2
CDKN1A	2
CDS1	2
CDS2	2
CDX2	2
CEBPD	2
CELF2	2
CELSR1	2
CELSR2	2

CENPF	2
CENPP	2
CEP164	2
CERCAM	2
CGNL1	2
CHD2	2
CHD4	2
CHD5	2
CHEK1	2
CHGA	2
CHMP1B	2
CHRD	2
CHRFAM7A	2
CHRNA7	2
CHST11	2
CHST8	2
CHTF8	2
CHUK	2
CISD1	2
CISD2	2
CKAP5	2
CLASP2	2
CLCN6	2
CLDN12	2
CLIP4	2
CLP1	2
CLPB	2
CMPK1	2
CMTM4	2
CMTM6	2
CNGA3	2
CNNM3	2
CNPY4	2
CNR1	2
CNTFR	2
CNTRL	2
COIL	2
COL11A1	2

COL14A1	2
COL15A1	2
COL1A1	2
COL24A1	2
COL3A1	2
COL4A1	2
COL4A2	2
COL4A3BP	2
COL4A5	2
COL4A6	2
COL5A1	2
COL5A2	2
COL9A1	2
COL9A3	2
COLEC10	2
COMMD3-BMI1	2
COPS7B	2
CORO1C	2
CPA4	2
CPED1	2
CPM	2
CPSF4	2
CRB2	2
CRBN	2
CRCT1	2
CREB3L1	2
CRISPLD2	2
CRNKL1	2
CRTAP	2
CRTC1	2
CSE1L	2
CSF1R	2
CTC-432M15.3	2
CTDSPL	2
CTNS	2
CTTN	2
CTTNBP2NL	2
CUL3	2

CXorf36	2
CYBRD1	2
CYP19A1	2
CYP46A1	2
CYTH1	2
DAB2	2
DAP	2
DAPK1	2
DARS2	2
DAZAP2	2
DCAF15	2
DCAF16	2
DCC	2
DCLK3	2
DCP2	2
DCUN1D2	2
DDHD1	2
DDX19A	2
DDX19B	2
DDX26B	2
DDX42	2
DDX6	2
DENND2C	2
DEPTOR	2
DERL3	2
DGCR2	2
DHDDS	2
DHX15	2
DHX33	2
DHX57	2
DIEXF	2
DIP2A	2
DIXDC1	2
DKFZP761J1410	2
DLG5	2
DLK1	2
DLST	2
DMP1	2

DNA2	2
DNAJB1	2
DNAJB12	2
DNAJC10	2
DNER	2
DOCK10	2
DOCK11	2
DOK6	2
DPF2	2
DPH1	2
DPH3	2
DPP3	2
DPY30	2
DPYSL5	2
DSCAM	2
DSG3	2
DUSP4	2
DUSP9	2
DYNLL2	2
DZIP1	2
E2F1	2
E2F6	2
EBF1	2
EBF2	2
EDEM3	2
EDN1	2
EEF2K	2
EFHD2	2
EFNA3	2
EFS	2
EGLN2	2
EIF2B1	2
EIF2B2	2
EIF2S2	2
EIF4A2	2
EIF4B	2
EIF4E3	2
EIF5B	2

ELAVL2	2
ELAVL4	2
ELF4	2
ELK4	2
ELOVL4	2
ELOVL6	2
EMILIN2	2
EML5	2
EMX2	2
ENPEP	2
ENTPD4	2
ENTPD7	2
EOMES	2
EPC2	2
EPHA7	2
EPOR	2
EPT1	2
ERBB3	2
ERCC4	2
ERCC6	2
ERF	2
ERG	2
ERGIC1	2
ERGIC2	2
ERN1	2
ESPL1	2
ETFA	2
ETS2	2
ETV5	2
EVI5L	2
EZH1	2
FADS3	2
FAM102A	2
FAM103A1	2
FAM135B	2
FAM149B1	2
FAM155B	2
FAM160A1	2

FAM168B	2
FAM171A1	2
FAM189A1	2
FAM199X	2
FAM204A	2
FAM20B	2
FAM214B	2
FAM46A	2
FAM73B	2
FAM84B	2
FANCF	2
FBXO21	2
FBXO30	2
FBXO34	2
FBXO41	2
FBXO48	2
FBXW7	2
FGD4	2
FGD6	2
FGF5	2
FGFR3	2
FICD	2
FKBP15	2
FKRP	2
FLRT3	2
FLT1	2
FNBP1L	2
FNIP1	2
FOPNL	2
FOXF2	2
FOXM1	2
FOXO3	2
FRK	2
FRMPD4	2
FRYL	2
FSTL4	2
FTL	2
FURIN	2

FUS	2
FZD4	2
FZD8	2
G3BP2	2
GABPA	2
GABPB2	2
GABRA6	2
GALC	2
GALE	2
GALNT15	2
GATAD2B	2
GATM	2
GDAP1	2
GDAP2	2
GDF11	2
GEMIN7	2
GGA1	2
GGCX	2
GIPC1	2
GIT2	2
GJA1	2
GJD2	2
GLB1L	2
GLCCI1	2
GLRX	2
GMCL1	2
GMEB1	2
GMPPB	2
GMPS	2
GNAI1	2
GNE	2
GNG5	2
GNPTAB	2
GNPTG	2
GOLGA3	2
GOLGA4	2
GOLGA7	2
GOLT1B	2

GOPC	2
GPATCH3	2
GPR107	2
GPR132	2
GPR135	2
GPR137	2
GPR156	2
GPR157	2
GPR26	2
GPR63	2
GPR75	2
GPR88	2
GPRIN3	2
GRASP	2
GRB10	2
GREB1	2
GRHL2	2
GRID1	2
GRID2IP	2
GRIN3A	2
GRM1	2
GRM4	2
GRPEL2	2
GSC	2
GSG1L	2
GTF2A1	2
GTF2I	2
GTPBP1	2
GTPBP2	2
GTPBP8	2
GUCY1A2	2
GYG2	2
H2AFX	2
H3F3B	2
HABP4	2
HAND1	2
HBP1	2
HDAC7	2
HDAC9	2
HDHD1	2
HDX	2
HECTD4	2
HEG1	2
HEPHL1	2
HERPUD1	2
HEY1	2
HGF	2
HID1	2
HIPK1	2
HIPK3	2
HIVEP2	2
HK2	2
HLCS	2
HMGA1	2
HNRNPC	2
HNRNPUL1	2
HOOK1	2
HOXA10	2
HOXB1	2
HOXC11	2
HOXC8	2
HRK	2
HSPA4	2
HSPB3	2
HSPBAP1	2
HSPE1-MOB4	2
HSPH1	2
HTR1E	2
HUNK	2
ICK	2
ICMT	2
ICOS	2
IDE	2
IFT57	2
IGDCC4	2
IGLON5	2

IGSF10	2
IGSF1	2
IKBKAP	2
IKBKE	2
IKZF2	2
IKZF3	2
IL10	2
IL13	2
IL1A	2
IL1R1	2
IL6ST	2
ILDR2	2
IMPDH1	2
INHBA	2
INSIG2	2
INSM2	2
INSR	2
INTS2	2
INTU	2
INVS	2
IPO11	2
IPO7	2
IPPK	2
IQCB1	2
IQSEC2	2
IRAK2	2
IREB2	2
IRGQ	2
ISL2	2
ISLR	2
ITGA6	2
ITPA	2
IVNS1ABP	2
JAKMIP3	2
JARID2	2
JOSD1	2
KCNA6	2
KCNAB1	2

KCNB1	2
KCNC1	2
KCNC2	2
KCNH1	2
KCNH5	2
KCNJ11	2
KCNJ3	2
KCNK1	2
KCNN3	2
KCNQ4	2
KCNS2	2
KCNS3	2
KCTD15	2
KCTD17	2
KCTD2	2
KCTD9	2
KDM2A	2
KDM2B	2
KDM3A	2
KDM6A	2
KDSR	2
KIAA0087	2
KIAA0141	2
KIAA0195	2
KIAA0355	2
KIAA0368	2
KIAA0391	2
KIAA0895L	2
KIAA0930	2
KIAA1147	2
KIAA1161	2
KIAA1217	2
KIAA1324L	2
KIAA1462	2
KIAA1467	2
KIAA1644	2
KIAA1919	2
KIAA2018	2

KIF14	2
KIF3A	2
KIN	2
KLC2	2
KLF15	2
KLF5	2
KLF9	2
KLHDC8B	2
KLHDC9	2
KLHL18	2
KLHL2	2
KLHL23	2
KLHL28	2
KLHL5	2
KLHL6	2
KLK10	2
KLLN	2
KMT2C	2
KMT2D	2
KNSTRN	2
KPNA5	2
KPNA6	2
KRBOX4	2
KRI1	2
KRIT1	2
L1CAM	2
L2HGDH	2
LAGE3	2
LARP1	2
LAS1L	2
LCLAT1	2
LCORL	2
LDB1	2
LDLR	2
LDLRAD4	2
LEMD3	2
LGR4	2
LHFPL3	2
LHX1	2
LIF	2
LINGO1	2
LIPH	2
LLPH	2
LMX1A	2
LONRF1	2
LOR	2
LOX	2
LOXL3	2
LOXL4	2
LPIN3	2
LPPR4	2
LRCH2	2
LRFN4	2
LRRC10B	2
LRRC20	2
LRRC34	2
LRRC59	2
LRRC7	2
LRRC8D	2
LSM11	2
LTB4R	2
LTN1	2
LUC7L2	2
LUC7L3	2
LYRM1	2
MAGI1	2
MAGIX	2
MAK16	2
MANEAL	2
MAP3K13	2
MAP7	2
MAPK14	2
MAPK8IP3	2
MAPRE2	2
MARC1	2
MARK2	2

MARS2	2
MAT2A	2
MB21D2	2
MBD1	2
MBNL1	2
MBOAT1	2
MBOAT2	2
MBTD1	2
MCMBP	2
MCTP2	2
MCTS1	2
MDFI	2
MDK	2
MECOM	2
MECP2	2
MED1	2
MED13	2
MED21	2
MED22	2
MED26	2
MED6	2
MEGF11	2
MEOX1	2
MESDC1	2
METTL10	2
METTL20	2
MFN1	2
MFSD4	2
MFSD6	2
MGAT5	2
MGRN1	2
MICALCL	2
MIEF1	2
MINK1	2
MIOS	2
MLLT3	2
MLLT6	2
MLTK	2
MMP11	2
MMP14	2
MMP19	2
MOB1B	2
MOB4	2
MORC4	2
MPP5	2
MPPED2	2
MPRIP	2
MPV17L	2
MRFAP1	2
MRPL11	2
MRPL22	2
MRS2	2
MSANTD3	2
MSL1	2
MSL2	2
MSN	2
MTMR10	2
MTSS1	2
MUT	2
MVB12B	2
MYBL1	2
MYEF2	2
MYL12A	2
MYO10	2
MYO1C	2
MYO1E	2
MYO1F	2
MYO5B	2
MYO9A	2
MYPN	2
MYRF	2
MYT1L	2
NAA40	2
NAALADL2	2
NACC1	2
NAGPA	2

NAP1L5	2
NAPEPLD	2
NBEA	2
NBEAL2	2
NCAPD3	2
NCDN	2
NCOR1	2
NDNF	2
NDRG3	2
NDRG4	2
NDST2	2
NDUFS1	2
NECAP2	2
NEK3	2
NEURL1B	2
NF1	2
NFASC	2
NFATC2IP	2
NHLRC2	2
NHS	2
NID1	2
NID2	2
NIPAL1	2
NIPBL	2
NKD1	2
NKIRAS2	2
NKRF	2
NKX2-4	2
NLGN2	2
NME4	2
NME6	2
NMNAT2	2
NMT1	2
NNT	2
NOL4	2
NONO	2
NOS1	2
NPFFR1	2

NPTN	2
NPTX1	2
NR1D2	2
NR4A2	2
NR4A3	2
NREP	2
NRL	2
NSG1	2
NSMCE2	2
NT5DC3	2
NUDT3	2
NUP50	2
NUP88	2
NXPH4	2
NYNRIN	2
OCRL	2
OGFRL1	2
OLR1	2
OSMR	2
OSTF1	2
OTOF	2
OTUB2	2
P2RX1	2
P2RX7	2
P4HA2	2
PACS1	2
PAFAH1B1	2
PAFAH1B2	2
PAG1	2
PAIP2B	2
PAK4	2
PAK6	2
PALD1	2
PALM3	2
PAN2	2
PANK3	2
PANX2	2
PAPOLA	2

PARD6G	2
PATL1	2
PAX2	2
PAX3	2
PAX5	2
PAX6	2
PCDH20	2
PCDH8	2
PCNP	2
PCNX	2
PCSK6	2
PCSK7	2
PCTP	2
PCYT1B	2
PDAP1	2
PDE3A	2
PDE7B	2
PDGFB	2
PDHB	2
PDIK1L	2
PDK4	2
PDPR	2
PDS5A	2
PDXDC1	2
PEA15	2
PEAK1	2
PER1	2
PER2	2
PEX11B	2
PFN1	2
PGRMC2	2
PHF1	2
PHF12	2
PHF15	2
PHLDA1	2
PIANP	2
PIAS1	2
PIAS4	2

PIGA	2
PIGU	2
PIK3C2A	2
PITPNB	2
PITPNM2	2
PITPNM3	2
PKNOX1	2
PKNOX2	2
PLA2G15	2
PLA2G3	2
PLAGL2	2
PLCB1	2
PLCL1	2
PLD3	2
PLD5	2
PLEKHA5	2
PLEKHG7	2
PLEKHH1	2
PLOD2	2
PLXNA2	2
PLXND1	2
PMAIP1	2
PMPCB	2
PNISR	2
PNRC1	2
PODXL	2
POGLUT1	2
POGZ	2
POLE4	2
POLR1C	2
POLR2D	2
POMGNT1	2
POTEG	2
POTEM	2
POU4F1	2
POU6F2	2
PP13439	2
PPAPDC2	2

PPARA	2
PPARGC1A	2
PPCS	2
PPIL2	2
PPIL6	2
PPIP5K2	2
PPM1H	2
PPP1R13L	2
PPP1R2	2
PPP1R3F	2
PPP2CA	2
PPP2R2A	2
PPP3R1	2
PQLC2	2
PRC1	2
PRDM5	2
PREPL	2
PRKACA	2
PRKAG2	2
PRKCE	2
PRMT10	2
PROX2	2
PRPF38B	2
PRPF4	2
PRPS1	2
PRR18	2
PRRT2	2
PRRT3	2
PSAP	2
PSMF1	2
PSORS1C2	2
PTBP3	2
PTCHD2	2
PTGFRN	2
PTP4A1	2
PTPLAD1	2
PTPN4	2
PTPN9	2
PTPRB	2
PTPRU	2
PURA	2
PURG	2
PUS7	2
PXMP4	2
PXT1	2
QARS	2
QSER1	2
QSOX2	2
RAB10	2
RAB21	2
RAB23	2
RAB39B	2
RAB3IL1	2
RAB40C	2
RABIF	2
RAD18	2
RAD9A	2
RADIL	2
RAG1	2
RALA	2
RALGAPB	2
RALGPS2	2
RALY	2
RAPGEF4	2
RASA2	2
RASGRP1	2
RASL10A	2
RASSF2	2
RB1	2
RBBP5	2
RBBP8	2
RBFOX1	2
RBM23	2
RBM24	2
RBM27	2
RBM33	2

RBM38	2
RBM7	2
RBMS2	2
RBPJ	2
RCAN3	2
RCN1	2
RCOR1	2
RCSD1	2
RD3	2
RDH10	2
RECK	2
REEP2	2
REL	2
RELT	2
REPS2	2
RETSAT	2
RFX3	2
RFX6	2
RGAG1	2
RGS16	2
RGS6	2
RHOBTB3	2
RIMS2	2
RIN2	2
RIOK3	2
RLF	2
RMDN3	2
RMND5A	2
RNF130	2
RNF144B	2
RNF169	2
RNF170	2
RNF185	2
RNF19B	2
RNF2	2
RNF24	2
RNF34	2
RNF43	2

RNF7	2
RNF8	2
RNFT1	2
ROBO1	2
ROBO2	2
ROR1	2
RORC	2
RP11-122A3.2	2
RP11-480I12.4	2
RP11-796G6.2	2
RPA3-AS1	2
RPL15	2
RPUSD2	2
RRM2	2
RSBN1L	2
RTCA	2
RUFY3	2
RUNDC1	2
S100A8	2
S100PBP	2
S1PR2	2
SAMD8	2
SASH1	2
SBF2	2
SCARA3	2
SCN2A	2
SCN5A	2
SCO1	2
SCRT1	2
SCYL3	2
SDC1	2
SDHC	2
SDHD	2
SEC14L1	2
SEC14L5	2
SEC16B	2
SEC31B	2
SECISBP2	2

SEMA3A	2
SEMA5A	2
SEMA6D	2
SENP1	2
SEPHS1	2
SEPT7	2
SERBP1	2
SERPING1	2
SERTAD4	2
SETD6	2
SETD7	2
SFT2D3	2
SFTA3	2
SFXN5	2
SGK3	2
SH2D4B	2
SH3BP4	2
SH3PXD2A	2
SHB	2
SHCBP1	2
SHISA7	2
SHMT1	2
SHOC2	2
SHPK	2
SHROOM3	2
SIAH2	2
SIK3	2
SIN3A	2
SIN3B	2
SIPA1L1	2
SIPA1L2	2
SLC10A3	2
SLC10A6	2
SLC12A9	2
SLC16A14	2
SLC19A2	2
SLC1A1	2
SLC1A4	2
SLC20A1	2
SLC24A3	2
SLC24A4	2
SLC25A10	2
SLC25A18	2
SLC25A27	2
SLC25A32	2
SLC26A4	2
SLC28A3	2
SLC2A12	2
SLC2A14	2
SLC2A3	2
SLC30A4	2
SLC30A6	2
SLC31A1	2
SLC35D2	2
SLC35E1	2
SLC35G1	2
SLC36A4	2
SLC38A9	2
SLC39A1	2
SLC45A4	2
SLC4A1	2
SLC4A4	2
SLC52A3	2
SLC5A9	2
SLC6A1	2
SLC6A15	2
SLC6A5	2
SLC7A11	2
SLC8A2	2
SLC9A2	2
SLC9A6	2
SLC9A9	2
SLCO1A2	2
SLCO5A1	2
SLIT1	2
SLIT3	2

SLITRK1	2
SLITRK4	2
SLK	2
SLMAP	2
SMAD5	2
SMAD7	2
SMAP2	2
SMARCD1	2
SMG6	2
SMIM15	2
SMTNL2	2
SMU1	2
SMUG1	2
SND1	2
SNPH	2
SNX16	2
SNX27	2
SNX6	2
SOBP	2
SOCS3	2
SOCS5	2
SOCS6	2
SOGA2	2
SOGA3	2
SON	2
SORCS1	2
SORT1	2
SOS1	2
SOWAHB	2
SOX13	2
SOX7	2
SP8	2
SPCS2	2
SPEG	2
SPEN	2
SPOCD1	2
SPOCK1	2
SPOPL	2

SPRY3	2
SPRYD4	2
SPTBN4	2
SPTSSB	2
SPTY2D1	2
SRCAP	2
SRD5A3	2
SREBF2	2
SRP19	2
SRRD	2
SRSF6	2
SS18	2
SSPN	2
SSX2IP	2
ST6GAL2	2
ST7-OT4	2
STAB2	2
STAG2	2
STAM2	2
STARD3NL	2
STARD4	2
STARD9	2
STAT4	2
STEAP3	2
STK10	2
STK17A	2
STK35	2
STK38	2
STK40	2
STK4	2
STRBP	2
STRIP2	2
STX3	2
STX5	2
STYX	2
SUB1	2
SUCLG2	2
SULF2	2

SUPT16H	2
SURF4	2
SUV39H2	2
SUV420H1	2
SVIP	2
SWT1	2
SYBU	2
SYNJ2BP	2
SYNPO	2
SYT6	2
SYT7	2
TAB2	2
TAB3	2
TACC1	2
TANC1	2
TAPT1	2
TARBP2	2
TAX1BP1	2
TBC1D12	2
TBC1D15	2
TBCEL	2
TBKBP1	2
TBX2	2
TBX4	2
TBX5	2
TCEB1	2
TCF7L2	2
TEAD1	2
TEAD3	2
TENM1	2
TENM2	2
TENM4	2
TERF2	2
TEX261	2
TFAP2C	2
TFCP2	2
TFR2	2
TFRC	2

TGDS	2
TGIF2	2
TGM2	2
THAP1	2
THAP9	2
THBS2	2
THOC2	2
THRB	2
THRSP	2
THUMPD3	2
TIFAB	2
TIMM17B	2
TIMM8B	2
TIMP3	2
TLL2	2
TM7SF2	2
TM9SF3	2
TMC7	2
TMED5	2
TMEM108	2
TMEM115	2
TMEM123	2
TMEM127	2
TMEM132B	2
TMEM132D	2
TMEM143	2
TMEM165	2
TMEM167A	2
TMEM192	2
TMEM194A	2
TMEM200C	2
TMEM233	2
TMEM234	2
TMEM236	2
TMEM245	2
TMEM251	2
TMEM26	2
TMEM43	2

TMEM65	2
TMEM87B	2
TMPPE	2
TMPRSS2	2
TMTC3	2
TMX4	2
TNRC6A	2
TNS1	2
TOB2	2
TOMM70A	2
TOPBP1	2
TOX	2
TPCN1	2
TPM3	2
TPP1	2
TPST2	2
TRABD	2
TRAF7	2
TRAK2	2
TRAPPC8	2
TRERF1	2
TRIAP1	2
TRIB1	2
TRIB2	2
TRIM17	2
TRIM2	2
TRNP1	2
TSC22D3	2
TSC22D4	2
TSKU	2
TSPAN2	2
TSPEAR	2
TSPYL5	2
TTC31	2
TTC39A	2
TTC39C	2
TTL	2
TTYH3	2

TUB	2
TUBD1	2
TUSC2	2
TWF1	2
TWIST1	2
TXK	2
TYRO3	2
UBE2D2	2
UBE2D3	2
UBE2G2	2
UBE2K	2
UBE2R2	2
UBE2T	2
UBE2W	2
UBE2Z	2
UBE3C	2
UBXN4	2
UCK2	2
UFM1	2
UHMK1	2
ULK1	2
UNC13A	2
UNC5D	2
UNC80	2
UQCC1	2
USP13	2
USP14	2
USP20	2
USP21	2
USP32	2
USP33	2
USP38	2
USP49	2
USP6NL	2
USP8	2
UTP15	2
UTRN	2
VAPA	2

VASH2	2
VAV2	2
VAV3	2
VDAC1	2
VGLL4	2
VIM	2
VMP1	2
VPS36	2
VPS39	2
VPS53	2
VSNL1	2
VTI1A	2
WDFY3	2
WDR20	2
WDR82	2
WHSC1	2
WLS	2
WNK1	2
WNK2	2
WNT2B	2
XK	2
XKR7	2
XKR8	2
XPO1	2
XPOT	2
XRCC5	2
YES1	2
YIPF4	2
YPEL2	2
YTHDC1	2
YTHDF2	2
YWHAG	2
YWHAH	2
YWHAQ	2
ZBED6	2
ZBTB26	2
ZBTB5	2
ZBTB7B	2
ZC3H12C	2
ZC3H3	2
ZC3H6	2
ZC3HAV1L	2
ZCCHC8	2
ZDHHC5	2
ZDHHC7	2
ZER1	2
ZFAND6	2
ZFP30	2
ZFP62	2
ZFPM2	2
ZHX3	2
ZIC2	2
ZIK1	2
ZKSCAN5	2
ZMIZ1	2
ZNF10	2
ZNF140	2
ZNF202	2
ZNF280C	2
ZNF282	2
ZNF302	2
ZNF322	2
ZNF341	2
ZNF354A	2
ZNF354B	2
ZNF362	2
ZNF391	2
ZNF426	2
ZNF449	2
ZNF462	2
ZNF473	2
ZNF487	2
ZNF514	2
ZNF521	2
ZNF532	2
ZNF566	2

ZNF583	2
ZNF609	2
ZNF618	2
ZNF620	2
ZNF641	2
ZNF667	2
ZNF689	2
ZNF74	2
ZNF774	2
ZNF781	2
ZNF783	2
ZNF784	2
ZNF800	2
ZNF804B	2
ZNF827	2
ZNF831	2
ZNF879	2
ZSWIM4	2
ZSWIM5	2
ZSWIM6	2
ZXDC	2
AARS2	1
AATK	1
ABCA1	1
ABCA2	1
ABCA9	1
ABCB7	1
ABCD2	1
ABCD3	1
ABCE1	1
ABHD17B	1
ABHD2	1
ABHD5	1
ABI1	1
ABI3BP	1
ABLIM1	1
ABR	1
ABTB2	1
AC003102.1	1
AC005003.1	1
AC006486.1	1
AC007375.1	1
AC079210.1	1
ACAA2	1
ACBD4	1
ACHE	1
ACP1	1
ACSL4	1
ACTA2	1
ACTN1	1
ACTR2	1
ACTRT3	1
ACVR1	1
ADAM22	1
ADAMTS17	1
ADAMTSL1	1
ADAMTSL2	1
ADAMTSL3	1
ADARB2	1
ADCY2	1
ADCYAP1R1	1
ADM	1
ADNP	1
ADRA1A	1
ADRA2C	1
ADRBK1	1
ADSS	1
AF196779.12	1
AFAP1	1
AFG3L2	1
AGAP2	1
AGAP9	1
AGPAT5	1
AHCYL1	1
AHNAK	1
AHR	1

AIDA	1
AK1	1
AK5	1
AKAP1	1
AKIRIN1	1
AL021546.6	1
AL590822.1	1
ALG14	1
ALG2	1
ALPK2	1
AMBRA1	1
AMER2	1
AMN1	1
ANAPC16	1
ANGEL1	1
ANGPT4	1
ANGPTL1	1
ANK1	1
ANKHD1-EIF4EBP3	1
ANKRD12	1
ANKRD27	1
ANKRD29	1
ANKRD34A	1
ANKRD50	1
ANKRD65	1
ANLN	1
ANO1	1
ANO3	1
ANP32A	1
ANP32B	1
ANTXR2	1
ANXA7	1
AP1AR	1
AP1G1	1
AP1M2	1
AP1S3	1
AP2B1	1
AP2M1	1

APBA2	1
APLN	1
APLP2	1
APOB	1
APOLD1	1
APOO	1
APOOL	1
APPL2	1
AQPEP	1
AR	1
ARAP2	1
ARC	1
ARCN1	1
ARF6	1
ARFGAP1	1
ARHGAP1	1
ARHGAP11A	1
ARHGAP12	1
ARHGAP19	1
ARHGAP19-SLIT1	1
ARHGAP26	1
ARHGAP29	1
ARHGAP31	1
ARHGAP5	1
ARHGAP6	1
ARHGDIA	1
ARHGEF26	1
ARHGEF37	1
ARHGEF5	1
ARID1A	1
ARID4A	1
ARID4B	1
ARIH1	1
ARIH2	1
ARL1	1
ARL3	1
ARL4C	1
ARL8A	1

ARNT2	1
ARPC4-TTLL3	1
ARSA	1
ARSB	1
AS3MT	1
ASAH2C	1
ASAP2	1
ASB14	1
ASB6	1
ASB7	1
ASIC1	1
ASIC4	1
ASNA1	1
ATG13	1
ATG2B	1
ATG4A	1
ATG4D	1
ATL2	1
ATM	1
ATMIN	1
ATOH8	1
ATP10A	1
ATP11C	1
ATP1A2	1
ATP1B2	1
ATP1B3	1
ATP2C1	1
ATP6V0A2	1
ATP6V1B2	1
ATP8A2	1
ATP8B1	1
ATP9A	1
ATRNL1	1
AVPI1	1
AXL	1
B3GNT4	1
B4GALNT1	1
B4GALT1	1
B4GALT2	1
B4GALT5	1
BAAT	1
BAG5	1
BAMBI	1
BAP1	1
BARHL2	1
BAZ1A	1
BAZ2B	1
BCAT2	1
BCL11B	1
BCL2L13	1
BCL2L2	1
BCL3	1
BCL6	1
BCL9	1
BCORL1	1
BCR	1
BDNF	1
BEST4	1
BET1L	1
BHLHE40	1
BHLHE41	1
BICC1	1
BICD2	1
BLOC1S3	1
BMP8A	1
BMP8B	1
BMPER	1
BMPR1B	1
BNIP2	1
BNIP3	1
BNIP3L	1
BRAP	1
BRE	1
BRMS1L	1
BTBD11	1
BZRAP1	1

C10orf131	1
C10orf32	1
C10orf54	1
C11orf96	1
C12orf43	1
C12orf66	1
C12orf73	1
C12orf79	1
C15orf52	1
C16orf87	1
C17orf100	1
C19orf40	1
C1orf106	1
C1orf131	1
C1orf168	1
C1orf198	1
C1orf50	1
C20orf196	1
C20orf27	1
C22orf15	1
C22orf31	1
C2CD2	1
C2orf42	1
C2orf68	1
C2orf71	1
C2orf72	1
C3orf65	1
C3orf70	1
C5orf24	1
C6orf106	1
C6orf222	1
C6orf223	1
C6orf47	1
C6orf62	1
C6orf89	1
C8orf37	1
C8orf4	1
C8orf46	1

C9orf69	1
C9orf72	1
CA10	1
CAB39L	1
CACHD1	1
CACNA1B	1
CADM4	1
CALR	1
CAMK2B	1
CAMK2D	1
CAMK2G	1
CAMKK1	1
CAMKK2	1
CAMTA2	1
CAPN5	1
CARD11	1
CARM1	1
CASD1	1
CASP10	1
CAST	1
CASZ1	1
CBFB	1
CBLN2	1
CBLN4	1
CBR4	1
CBX4	1
CBX8	1
CC2D1A	1
CCAR1	1
CCDC126	1
CCDC134	1
CCDC149	1
CCDC152	1
CCDC160	1
CCDC171	1
CCDC177	1
CCDC41	1
CCDC50	1

CCDC58	1
CCDC64	1
CCDC85A	1
CCDC85C	1
CCER1	1
CCL4	1
CCL4L1	1
CCL8	1
CCM2	1
CCNA2	1
CCNB1	1
CCND3	1
CCNDBP1	1
CCNG1	1
CCNI2	1
CCNK	1
CCNL1	1
CCP110	1
CCRN4L	1
CCT3	1
CD163	1
CD164L2	1
CD28	1
CD4	1
CD47	1
CD69	1
CD84	1
CD96	1
CDC14A	1
CDC37	1
CDC42BPG	1
CDC5L	1
CDH11	1
CDH8	1
CDK1	1
CDK17	1
CDK18	1
CDK19	1

CDK8	1
CDKL2	1
CDKN1B	1
CDKN1C	1
CDON	1
CEBPA	1
CELF6	1
CENPO	1
CEP128	1
CEP170	1
CEP170B	1
CEP250	1
CEP55	1
CEP76	1
CEP97	1
CERS2	1
CERS6	1
CFL1	1
CGGBP1	1
CGN	1
CHAMP1	1
CHCHD4	1
CHD1	1
CHI3L1	1
CHL1	1
CHMP1A	1
CHMP2B	1
CHMP3	1
CHMP4B	1
CHRNB2	1
CHST10	1
CHURC1	1
CIAPIN1	1
CINP	1
CISD3	1
CITED2	1
CITED4	1
CKAP4	1

CLCC1	1
CLCN2	1
CLCN3	1
CLEC2D	1
CLIC5	1
CLLU1	1
CLMN	1
CLMP	1
CLPTM1L	1
CLSPN	1
CLSTN2	1
CLTC	1
CLTCL1	1
CLUH	1
CLVS1	1
CMPK2	1
CMTM7	1
CNIH4	1
CNN2	1
CNN3	1
CNOT1	1
CNTN1	1
CNTN2	1
CNTN4	1
CNTNAP2	1
CNTNAP5	1
COG3	1
COL13A1	1
COL16A1	1
COL19A1	1
COL23A1	1
COL25A1	1
COL26A1	1
COL4A4	1
COLQ	1
COMMD10	1
COMTD1	1
COPS5	1

COQ10B	1
COQ6	1
COX15	1
COX8C	1
CPNE2	1
CPPED1	1
CPSF6	1
CPT1A	1
CR2	1
CRCP	1
CREG2	1
CRIM1	1
CRISP3	1
CRISPLD1	1
CRMP1	1
CRTC2	1
CRYBG3	1
CS	1
CSF2RB	1
CSK	1
CSMD1	1
CSNK1A1	1
CSNK1G3	1
CSPP1	1
CSRNP1	1
CSRNP2	1
CSTF3	1
CTD-2207O23.12	1
CTD-2228K2.5	1
CTDP1	1
CTDSP1	1
CTDSP2	1
CTGF	1
CTNND1	1
CUL4A	1
CXCR5	1
CXXC5	1
CYB561	1

CYB5B	1
CYCS	1
CYP1B1	1
CYP26B1	1
CYP4F22	1
CYR61	1
CYTH2	1
DAAM1	1
DAAM2	1
DAB2IP	1
DALRD3	1
DBP	1
DCAF11	1
DCAF7	1
DCBLD1	1
DCHS1	1
DCLK1	1
DCUN1D5	1
DDA1	1
DDAH1	1
DDIT4	1
DDTL	1
DDX5	1
DDX52	1
DEDD	1
DEK	1
DENND1A	1
DENND4B	1
DENND4C	1
DENR	1
DEPDC1	1
DERL2	1
DGKE	1
DHCR24	1
DHRS3	1
DHX36	1
DIAPH1	1
DIO3	1

DIP2B	1
DIP2C	1
DIRAS2	1
DIRC2	1
DKFZP779J2370	1
DKK2	1
DLEU1	1
DLGAP3	1
DLL1	1
DLX3	1
DMRT3	1
DMRTA2	1
DMTN	1
DMXL1	1
DNAJA4	1
DNAJC13	1
DNAJC21	1
DNAJC5	1
DNAL4	1
DND1	1
DNMT3A	1
DOCK5	1
DOCK7	1
DOCK9	1
DOHH	1
DOK4	1
DOLPP1	1
DONSON	1
DPP6	1
DPY19L3	1
DPYSL2	1
DRAM1	1
DRD1	1
DRGX	1
DSC1	1
DSEL	1
DSG2	1
DTD2	1

DTNBP1	1
DUSP10	1
DUSP5	1
DUSP6	1
DUSP8	1
DUT	1
DYNC1I1	1
DYRK1B	1
E2F7	1
E2F8	1
ECT2L	1
EDA2R	1
EDAR	1
EDARADD	1
EED	1
EFR3A	1
EGF	1
EGFLAM	1
EGLN1	1
EGR1	1
EHBP1	1
EI24	1
EIF2AK2	1
EIF2S1	1
EIF3A	1
EIF3H	1
EIF4A1	1
EIF4E	1
EIF4EBP2	1
EIF4EBP3	1
ELAVL3	1
ELF3	1
ELK3	1
ELMO1	1
ELMOD2	1
ELN	1
ELOVL3	1
ELOVL5	1
ELP4	1
EMC1	1
EML1	1
EML4	1
EML6	1
EN2	1
ENC1	1
ENGASE	1
ENOX2	1
ENPP1	1
ENPP5	1
ENY2	1
EP300	1
EPAS1	1
EPB41	1
EPB41L1	1
EPB41L3	1
EPB41L4B	1
EPB41L5	1
EPG5	1
EPGN	1
EPHA8	1
EPHB1	1
EPHB3	1
EPHX3	1
EPHX4	1
EPS15	1
ERBB2	1
ERC1	1
ERC2	1
ERI1	1
ERLIN1	1
ERLIN2	1
ERMN	1
ESCO1	1
ESD	1
ESM1	1
ESYT1	1

ETF1	1
ETS1	1
ETV6	1
EVI2A	1
EXOC6B	1
EXOG	1
EXOSC2	1
EXOSC3	1
EYA1	1
F13A1	1
F2R	1
F3	1
FADS6	1
FAF1	1
FAM105B	1
FAM107A	1
FAM107B	1
FAM110B	1
FAM114A1	1
FAM122A	1
FAM127A	1
FAM127B	1
FAM127C	1
FAM133A	1
FAM13A	1
FAM13B	1
FAM151B	1
FAM160A2	1
FAM167A	1
FAM172A	1
FAM175B	1
FAM177B	1
FAM179A	1
FAM19A2	1
FAM216A	1
FAM216B	1
FAM3C	1
FAM45A	1
FAM46B	1
FAM49A	1
FAM53C	1
FAM58A	1
FAM60A	1
FAM69A	1
FAM76B	1
FAM78B	1
FAM83D	1
FAM83F	1
FAM89A	1
FAM8A1	1
FAM91A1	1
FANCC	1
FANCE	1
FBN1	1
FBRS	1
FBXL16	1
FBXL17	1
FBXL3	1
FBXL4	1
FBXO10	1
FBXO11	1
FBXO27	1
FBXO36	1
FBXO40	1
FBXO8	1
FBXW4	1
FCGRT	1
FCRLB	1
FDXR	1
FEM1B	1
FERMT1	1
FGF10	1
FGF14	1
FGF6	1
FGF9	1
FGFBP3	1

FHAD1	1
FHDC1	1
FHL1	1
FHL5	1
FIBIN	1
FKBP1A	1
FKBP4	1
FLAD1	1
FLJ00104	1
FLJ00388	1
FLJ27365	1
FLOT1	1
FMNL2	1
FN1	1
FNBP1	1
FNBP4	1
FNDC5	1
FOS	1
FOXB1	1
FOXD4	1
FOXD4L1	1
FOXD4L2	1
FOXK1	1
FOXK2	1
FOXN1	1
FOXN2	1
FOXN3	1
FOXO1	1
FOXP4	1
FOXRED2	1
FRMD4A	1
FRMD6	1
FRMPD2	1
FSCN1	1
FSD1L	1
FSTL1	1
FTO	1
FTSJ2	1

FUT4	1
FYCO1	1
FYTTD1	1
FZD5	1
FZD6	1
G2E3	1
G6PC3	1
G6PD	1
GABARAPL1	1
GABBR1	1
GABRB2	1
GABRQ	1
GAD1	1
GAL3ST3	1
GAL3ST4	1
GALNT10	1
GALNT16	1
GATC	1
GATSL3	1
GBA	1
GBA2	1
GCC1	1
GCC2	1
GCNT1	1
GCNT2	1
GDA	1
GDI1	1
GDNF	1
GIT1	1
GJA3	1
GK	1
GLIPR2	1
GLIS2	1
GLP1R	1
GLRX2	1
GLTP	1
GLYR1	1
GNA13	1

GNAI3	1
GNAO1	1
GNB1L	1
GNB2L1	1
GNG2	1
GOLGA6A	1
GOLGA6B	1
GOLGA6C	1
GOLGA6D	1
GOLGA8A	1
GOLGA8B	1
GOLGA8F	1
GOLGA8G	1
GPATCH2	1
GPC1	1
GPD2	1
GPHN	1
GPR124	1
GPR137B	1
GPR153	1
GPR155	1
GPR158	1
GPR173	1
GPR180	1
GPR22	1
GPR6	1
GPR85	1
GPRASP2	1
GPRC5A	1
GPSM1	1
GRB2	1
GRIA1	1
GRIA2	1
GRID2	1
GRIK4	1
GRINA	1
GRIP1	1
GRK6	1

GRM7	1
GRSF1	1
GRWD1	1
GSE1	1
GSK3B	1
GTDC1	1
GTF2H1	1
GUCD1	1
GULP1	1
H6PD	1
HAP1	1
HAPLN4	1
HAUS3	1
HAVCR1	1
HCN1	1
HCN2	1
HDAC2	1
HDAC4	1
HECTD3	1
HES1	1
HEXIM1	1
HINT3	1
HIST1H2BH	1
HIST1H3I	1
HM13	1
HMBS	1
HMCN1	1
HMGB1	1
HMGB2	1
HMGCLL1	1
HMGCS1	1
HMX1	1
HNF1B	1
HNF4A	1
HNRNPA0	1
HNRNPD	1
HNRNPH1	1
HNRNPK	1

HNRNPR	1
HNRNPU	1
HNRNPUL2	1
HOOK3	1
HOXA11	1
HOXA5	1
HOXB5	1
HOXB8	1
HOXC12	1
HOXD4	1
HPRT1	1
HRH1	1
HS3ST3A1	1
HS3ST5	1
HSBP1	1
HSDL1	1
HSP90B1	1
HSPA13	1
HSPA2	1
HSPA4L	1
HSPA5	1
HTR2C	1
HTRA3	1
HTT	1
HYOU1	1
IARS2	1
ID2	1
ID4	1
IER5L	1
IFNG	1
IFRG15	1
IGF2R	1
IGFBP3	1
IGFBP5	1
IGFN1	1
IGSF11	1
IL1RAP	1
IL21R	1
IL2RB	1
IL5RA	1
ILDR1	1
ILF3	1
IMPAD1	1
INHBB	1
INIP	1
INPP4A	1
INPP5E	1
INSL5	1
INTS6	1
IPO8	1
IQCJ-SCHIP1	1
IQGAP1	1
IQGAP2	1
IRAK1BP1	1
IRF1	1
IRF2BPL	1
IRS4	1
IRX4	1
ISL1	1
ISM2	1
ISPD	1
ITCH	1
ITFG3	1
ITGA11	1
ITGA2	1
ITGA3	1
ITPKB	1
ITPR1	1
ITPRIPL2	1
JAKMIP1	1
JAKMIP2	1
KALRN	1
KANK1	1
KARS	1
KAT2B	1
KAT6A	1

KAZN	1
KBTBD8	1
KCNA2	1
KCNIP1	1
KCNJ10	1
KCNJ13	1
KCNJ14	1
KCNJ5	1
KCNK12	1
KCNK5	1
KCNK9	1
KCNMB2	1
KCTD3	1
KCTD7	1
KDELR1	1
KIAA0101	1
KIAA0513	1
KIAA1024	1
KIAA1468	1
KIAA1551	1
KIAA1614	1
KIAA1671	1
KIAA2013	1
KIAA2026	1
KIF13A	1
KIF13B	1
KIF19	1
KIF1B	1
KIF26A	1
KIF5A	1
KIT	1
KLF10	1
KLF12	1
KLF2	1
KLF3	1
KLHL1	1
KLHL11	1
KLHL17	1
KLHL20	1
KLHL24	1
KLHL26	1
KLHL29	1
KLHL38	1
KLHL7	1
KLHL8	1
KLHL9	1
KNTC1	1
KRT222	1
KSR1	1
KTN1	1
L3MBTL3	1
LAMB3	1
LASP1	1
LCA5	1
LDLRAP1	1
LDOC1L	1
LGI1	1
LGI2	1
LHFPL2	1
LHX3	1
LHX6	1
LIG3	1
LIMS1	1
LIN7C	1
LIN9	1
LIPG	1
LLGL2	1
LMBR1	1
LMBR1L	1
LMBRD2	1
LMCD1	1
LMNB2	1
LMO1	1
LMOD1	1
LNPEP	1
LNX2	1

LPAR3	1
LPCAT1	1
LPCAT2	1
LRBA	1
LRCH1	1
LRCH3	1
LRFN2	1
LRIG1	1
LRP10	1
LRP1B	1
LRP4	1
LRRC16A	1
LRRC32	1
LRRC58	1
LRRC8C	1
LRRK1	1
LRRN1	1
LRRTM2	1
LSAMP	1
LSM10	1
LSM12	1
LSM14A	1
LUC7L	1
MAB21L2	1
MACF1	1
MADD	1
MAEA	1
MAF	1
MAFG	1
MAGEL2	1
MAGI2	1
MAGOHB	1
MAK	1
MAMDC2	1
MAML1	1
MAMSTR	1
MAN1A2	1
MAN1C1	1

MAN2A1	1
MAP1A	1
MAP2K1	1
MAP2K3	1
MAP2K6	1
MAP3K10	1
MAP3K12	1
MAP3K8	1
MAP4K2	1
MAP7D1	1
MAPK7	1
MAPKBP1	1
MAPRE1	1
MAPT	1
MARCH3	1
MARCH6	1
MARCKS	1
MARCKSL1	1
MARK1	1
MATN3	1
MATR3	1
MBD5	1
MCFD2	1
MCL1	1
MCTP1	1
MCUR1	1
MDFIC	1
MDGA1	1
MDN1	1
ME2	1
MED13L	1
MED14	1
MEGF9	1
MEIS1	1
MELK	1
MEMO1	1
MET	1
METAP1	1

METAP2	1
METRN	1
METTL8	1
MFAP2	1
MFAP3	1
MFSD9	1
MIA3	1
MICAL2	1
MICAL3	1
MID1	1
MID1IP1	1
MID2	1
MIDN	1
MIP	1
MIPOL1	1
MISP	1
MKNK2	1
MKRN1	1
MKX	1
MLF1	1
MLLT1	1
MLX	1
MMAB	1
MMD	1
MME	1
MMGT1	1
MMS19	1
MNT	1
MNX1	1
MOCS1	1
MORF4L1	1
MOSPD1	1
MPP1	1
MPP7	1
MRAS	1
MRC2	1
MROH8	1
MRPL17	1

MRPL4	1
MRPL45	1
MSANTD3-TMEFF1	1
MSI1	1
MSMO1	1
MST4	1
MTCH2	1
MTERFD1	1
MTF1	1
MTHFR	1
MTMR14	1
MTMR6	1
MTMR9	1
MTPN	1
MTX3	1
MYADM	1
MYB	1
MYBL2	1
MYCBP2	1
MYLIP	1
MYLK	1
MYO18A	1
MYO19	1
MYO9B	1
NAA15	1
NAA50	1
NAALADL1	1
NAB1	1
NAGA	1
NAIF1	1
NAMPT	1
NANP	1
NAPB	1
NAPRT1	1
NAV1	1
NAV2	1
NBEAL1	1
NCF1	1

NCKAP5	1
NCKAP5L	1
NCKIPSD	1
NCOA2	1
NCOA4	1
NDFIP2	1
NDRG1	1
NDRG2	1
NDUFA7	1
NDUFAF4	1
NEK10	1
NEK4	1
NEK6	1
NEK7	1
NET1	1
NEUROD2	1
NEUROD4	1
NEUROG1	1
NFAM1	1
NFATC4	1
NFXL1	1
NGDN	1
NGFR	1
NHSL2	1
NIPSNAP1	1
NKAIN2	1
NKAPL	1
NKX2-2	1
NKX3-2	1
NKX6-1	1
NME1	1
NME7	1
NNAT	1
NNMT	1
NOG	1
NOP9	1
NOTCH2	1
NPAS3	1

NPAT	1
NPEPPS	1
NPTX2	1
NPY2R	1
NR1H3	1
NR3C2	1
NR5A1	1
NRARP	1
NRBF2	1
NRBP1	1
NRCAM	1
NRD1	1
NRF1	1
NRP2	1
NRSN1	1
NRXN1	1
NRXN2	1
NSG2	1
NSMAF	1
NTN4	1
NTNG1	1
NUCB1	1
NUCKS1	1
NUDCD2	1
NUDCD3	1
NUDT13	1
NUMB	1
NUP54	1
OAS2	1
OCIAD2	1
ODC1	1
OGDH	1
OPHN1	1
ORC5	1
OSBP	1
OSBPL10	1
OSBPL2	1
OSBPL6	1

OSBPL8	1
OTC	1
OTOGL	1
OTUD1	1
OTUD4	1
OTUD5	1
OTUD6B	1
OXSR1	1
PA2G4	1
PABPC1	1
PABPC1L2A	1
PABPC1L2B	1
PAIP1	1
PAIP2	1
PAK3	1
PALLD	1
PAM	1
PAOX	1
PAPD4	1
PAPOLG	1
PAQR5	1
PARK2	1
PARL	1
PARP2	1
PARPBP	1
PARVB	1
PAX9	1
PC	1
PCBD2	1
PCBP2	1
PCDH12	1
PCDH17	1
PCDH18	1
PCMT1	1
PCMTD1	1
PCSK5	1
PDCD6IP	1
PDE11A	1

PDE1B	1
PDE4A	1
PDE7A	1
PDGFRB	1
PDHX	1
PDIA6	1
PDLIM2	1
PDPK1	1
PDPN	1
PDZD2	1
PDZD4	1
PELI3	1
PET117	1
PEX16	1
PEX19	1
PEX5L	1
PFKFB3	1
PFN2	1
PGK1	1
PGM3	1
PHAX	1
PHB2	1
PHC3	1
PHF14	1
PHF17	1
PHF20	1
PHF20L1	1
PHF2	1
PHF21A	1
PHF21B	1
PHF3	1
PHF7	1
PHLDA3	1
PHLDB1	1
PHYHIPL	1
PI4KA	1
PIAS2	1
PICALM	1

PIGG	1
PIGS	1
PIGV	1
PIK3AP1	1
PIKFYVE	1
PIM1	1
PIM2	1
PIP4K2B	1
PIP5K1B	1
PIRT	1
PITX1	1
PJA2	1
PKP2	1
PLA2G10	1
PLA2G2C	1
PLA2G4A	1
PLAA	1
PLAU	1
PLCB4	1
PLCE1	1
PLD1	1
PLEK	1
PLEKHA6	1
PLEKHA7	1
PLEKHG3	1
PLEKHH2	1
PLEKHM1	1
PLEKHM3	1
PLEKHO2	1
PLXNA3	1
PNPLA4	1
PNPO	1
PNRC2	1
POLA1	1
POLH	1
POLQ	1
POLR3G	1
POLR3H	1

POM121C	1
PON2	1
POSTN	1
POU3F2	1
POU3F3	1
PPAP2B	1
PPFIA1	1
PPFIA2	1
PPFIBP2	1
PPIG	1
PPIL1	1
PPL	1
PPM1D	1
PPM1F	1
PPP1R13B	1
PPP1R1C	1
PPP1R3B	1
PPP1R3D	1
PPP1R9A	1
PPP1R9B	1
PPP2R4	1
PPP2R5A	1
PPP3R2	1
PPP4R4	1
PPP6C	1
PPP6R1	1
PPP6R2	1
PPP6R3	1
PRAF2	1
PRDM16	1
PRDM4	1
PRIMA1	1
PRKAA1	1
PRKAR1A	1
PRKCA	1
PRKCD	1
PRKCH	1
PRKD1	1

PRKRIP1	1
PRKX	1
PROSER1	1
PRPF40A	1
PRPF4B	1
PRPS2	1
PRR11	1
PRR3	1
PRR5L	1
PRRC2C	1
PRRG3	1
PRSS8	1
PSD	1
PSD3	1
PSD4	1
PSKH1	1
PSME4	1
PSPC1	1
PTBP2	1
PTCHD4	1
PTGER4	1
PTGES3	1
PTGR2	1
PTK2B	1
PTPN14	1
PTPN21	1
PTPRE	1
PTPRF	1
PTPRN2	1
PUM1	1
PXK	1
QDPR	1
R3HDM2	1
RAB11FIP1	1
RAB11FIP2	1
RAB11FIP5	1
RAB13	1
RAB14	1
RAB19	1
RAB1A	1
RAB27B	1
RAB2B	1
RAB33B	1
RAB34	1
RAB35	1
RAB37	1
RAB3B	1
RAB3D	1
RAB3IP	1
RAB4A	1
RAB4B	1
RAB5C	1
RAB6B	1
RAB8A	1
RAB9B	1
RABGAP1	1
RABGEF1	1
RACGAP1	1
RAD21	1
RAD23A	1
RAD51D	1
RAD54L2	1
RAF1	1
RALBP1	1
RALGAPA2	1
RALGDS	1
RAN	1
RANBP10	1
RANBP3	1
RAP2A	1
RAPGEF3	1
RAPGEF5	1
RAPGEF6	1
RAPH1	1
RASD2	1
RASGEF1A	1

RASSF1	1
RASSF6	1
RBAK	1
RBBP4	1
RBBP7	1
RBM20	1
RBM22	1
RBM25	1
RBM26	1
RBM47	1
RBM48	1
RBM5	1
RBP4	1
RC3H1	1
RCC2	1
RCN3	1
RCOR3	1
RDH12	1
REEP3	1
RER1	1
REST	1
REV1	1
RFTN1	1
RFTN2	1
RFX1	1
RFX2	1
RFX4	1
RGL1	1
RGMA	1
RGMB	1
RGS21	1
RGS7BP	1
RHBDL1	1
RHD	1
RHEBL1	1
RHOB	1
RHOBTB1	1
RHOJ	1

RHOQ	1
RIC3	1
RIMBP2	1
RIMKLB	1
RMI2	1
RND3	1
RNF103-CHMP3	1
RNF111	1
RNF125	1
RNF138	1
RNF141	1
RNF146	1
RNF167	1
RNF182	1
RNF183	1
RNF19A	1
RNF208	1
RNF216	1
RNF222	1
RNF32	1
RNF40	1
RNF4	1
RNF5	1
RNPEPL1	1
ROCK1	1
RP11-181C3.1	1
RP11-195F19.5	1
RP11-644F5.10	1
RP11-676J12.7	1
RP11-793H13.10	1
RPAP3	1
RPH3A	1
RPL36A-HNRNPH2	1
RPN2	1
RPRD1B	1
RPRM	1
RPS6KA2	1
RPS6KA5	1

RRAGC	1
RRP12	1
RSPH10B	1
RTN1	1
RTN4	1
RTN4RL1	1
RUNDC3B	1
RUNX3	1
RWDD4	1
SACM1L	1
SAMD10	1
SAMHD1	1
SAR1A	1
SATB1	1
SBDS	1
SC5D	1
SCAMP5	1
SCARB1	1
SCARB2	1
SCARF1	1
SCEL	1
SCHIP1	1
SCML1	1
SCML4	1
SCN1B	1
SCN9A	1
SDC2	1
SEC11A	1
SEC22A	1
SEC24C	1
SEC24D	1
SEC61A1	1
SEC61A2	1
SEL1L	1
SEL1L3	1
SELK	1
SEMA3C	1
SEMA4A	1

SEMA4B	1
SEMA6A	1
SEMA6C	1
SEMA7A	1
SEPN1	1
SEPT11	1
SEPT3	1
SEPT8	1
SEPT9	1
SERPINE1	1
SERPINH1	1
SERTAD1	1
SESN1	1
SESN2	1
SETD9	1
SEZ6L2	1
SF1	1
SFRP4	1
SGCD	1
SGK1	1
SGMS1	1
SGOL1	1
SGTB	1
SH2D4A	1
SH3BGRL	1
SH3BGRL2	1
SH3BGRL3	1
SH3BP5	1
SH3D19	1
SH3GLB2	1
SH3PXD2B	1
SH3RF2	1
SHANK2	1
SHANK3	1
SHC4	1
SHE	1
SHFM1	1
SHOX	1

SHROOM2	1
SHROOM4	1
SIAE	1
SIAH3	1
SIK1	1
SIK2	1
SIM1	1
SIRPA	1
SIRT1	1
SIRT5	1
SIVA1	1
SIX1	1
SIX2	1
SIX4	1
SLA	1
SLC12A5	1
SLC12A6	1
SLC12A7	1
SLC16A2	1
SLC16A6	1
SLC17A6	1
SLC18A2	1
SLC1A2	1
SLC20A2	1
SLC23A2	1
SLC23A3	1
SLC24A2	1
SLC25A16	1
SLC25A25	1
SLC25A34	1
SLC25A36	1
SLC25A37	1
SLC25A44	1
SLC25A53	1
SLC25A6	1
SLC26A7	1
SLC27A1	1
SLC2A1	1

SLC2A4RG	1
SLC2A8	1
SLC30A10	1
SLC30A9	1
SLC31A2	1
SLC33A1	1
SLC35A1	1
SLC35A2	1
SLC35A3	1
SLC35B4	1
SLC35C1	1
SLC35C2	1
SLC35D1	1
SLC35E2B	1
SLC35F3	1
SLC37A1	1
SLC38A2	1
SLC39A13	1
SLC39A9	1
SLC41A1	1
SLC43A2	1
SLC44A1	1
SLC44A2	1
SLC4A10	1
SLC4A7	1
SLC6A11	1
SLC6A9	1
SLC7A6	1
SLC7A8	1
SLC9A1	1
SLC9A3R1	1
SLC9A8	1
SLCO2A1	1
SLCO3A1	1
SLFN12	1
SLX4	1
SMAD1	1
SMAGP	1

SMAP1	1
SMARCA2	1
SMARCA5	1
SMARCC2	1
SMCO1	1
SMG5	1
SMOC1	1
SMOC2	1
SMURF1	1
SMYD5	1
SNAI2	1
SNAP25	1
SNCAIP	1
SNIP1	1
SNTB1	1
SNTG1	1
SNX13	1
SNX18	1
SNX22	1
SNX4	1
SOCS2	1
SORL1	1
SOS2	1
SOX11	1
SOX2	1
SOX21	1
SP3	1
SP6	1
SPAG1	1
SPAG9	1
SPAST	1
SPATA12	1
SPCS1	1
SPECC1	1
SPECC1L	1
SPG20	1
SPHK2	1
SPIN3	1

SPP1	1
SPPL2A	1
SPRED2	1
SPRTN	1
SPRYD3	1
SPSB4	1
SPTBN2	1
SRCIN1	1
SRGAP2	1
SRL	1
SRM	1
SRRM4	1
SRSF10	1
SRSF11	1
SRSF5	1
SSBP3-AS1	1
SSH3	1
SSR1	1
SSTR1	1
ST3GAL2	1
ST3GAL5	1
ST6GALNAC3	1
ST8SIA3	1
STAG1	1
STAMBPL1	1
STAU1	1
STC1	1
STC2	1
STEAP2	1
STK17B	1
STK19	1
STK32B	1
STK33	1
STK39	1
STMND1	1
STRADB	1
STRN3	1
STX12	1

STX18	1
STX6	1
STXBP6	1
SUDS3	1
SUGT1	1
SUN1	1
SUPT6H	1
SUV420H2	1
SUZ12	1
SV2A	1
SWAP70	1
SYCN	1
SYN1	1
SYNE1	1
SYNE2	1
SYNPO2	1
SYNPR	1
SYP	1
SYPL1	1
SYPL2	1
SYS1	1
SYT3	1
SZRD1	1
SZT2	1
TADA2B	1
TAF13	1
TAF15	1
TAF4B	1
TAF7L	1
TARBP1	1
TARDBP	1
TARS	1
TAT	1
TBC1D1	1
TBC1D14	1
TBC1D16	1
TBC1D22B	1
TBC1D24	1

TBC1D25	1
TBC1D2B	1
TBC1D30	1
TBC1D4	1
TBC1D8	1
TBC1D9	1
TBK1	1
TBL1XR1	1
TBPL1	1
TBX1	1
TBXA2R	1
TCEB3	1
TCF19	1
TCF23	1
TCF7	1
TCP11L2	1
TDRD6	1
TECTB	1
TEF	1
TESK2	1
TEX2	1
TFAM	1
TFDP2	1
TGFBRAP1	1
TGIF1	1
TGM3	1
TGM5	1
TGOLN2	1
THBS3	1
THBS4	1
THTPA	1
TIAF1	1
TIAL1	1
TIAM1	1
TLCD2	1
TLL1	1
TLN1	1
TLN2	1

TM9SF4	1
TMBIM6	1
TMED2	1
TMED4	1
TMED8	1
TMEFF1	1
TMEM106A	1
TMEM120B	1
TMEM131	1
TMEM134	1
TMEM150C	1
TMEM159	1
TMEM161B	1
TMEM170B	1
TMEM181	1
TMEM194B	1
TMEM196	1
TMEM198	1
TMEM216	1
TMEM218	1
TMEM231	1
TMEM242	1
TMEM25	1
TMEM59	1
TMEM63B	1
TMEM67	1
TMEM68	1
TMEM71	1
TMEM75	1
TMEM86A	1
TMEM9	1
TMEM92	1
TMF1	1
TMOD1	1
TMUB2	1
TMX3	1
TNF	1
TNFAIP1	1

TNFAIP8	1
TNFRSF11B	1
TNFRSF12A	1
TNFRSF19	1
TNFSF11	1
TNFSF8	1
TNIP1	1
TNPO2	1
TOB1	1
TOM1L2	1
TOMM40	1
TOP1	1
TOR1B	1
TOX2	1
TOX3	1
TP53INP2	1
TP73	1
TPRG1	1
TPRG1L	1
TRIB3	1
TRIM23	1
TRIM3	1
TRIM33	1
TRIM37	1
TRIM52	1
TRIM66	1
TRIO	1
TRIP4	1
TRIQK	1
TRMT10B	1
TRMT1L	1
TRPC3	1
TRPM7	1
TRPV3	1
TRRAP	1
TSFM	1
TSHZ1	1
TSHZ2	1

TSNAX	1
TSPAN3	1
TSPAN33	1
TSPAN4	1
TSPAN9	1
TSPYL2	1
TTBK1	1
TTC16	1
TTC17	1
TTC39B	1
TTC9	1
TTC9C	1
TTLL7	1
TUBB	1
TULP4	1
TWISTNB	1
TXNDC12	1
TXNL1	1
TXNRD1	1
TYSND1	1
U2AF2	1
U2SURP	1
UBA6	1
UBALD1	1
UBAP2L	1
UBASH3B	1
UBE2A	1
UBE2B	1
UBE2D4	1
UBE2F	1
UBE2J1	1
UBE2L3	1
UBE2N	1
UBE2Q2	1
UBL3	1
UBN1	1
UBP1	1
UBR2	1

UBXN10	1
UBXN2B	1
UBXN7	1
UCK1	1
UCP2	1
UCP3	1
UHRF1BP1L	1
UNC13C	1
UNC93B1	1
URM1	1
USF2	1
USP2	1
USP36	1
USP37	1
USP42	1
USP5	1
USP9X	1
USP9Y	1
VASH1	1
VAT1	1
VCAM1	1
VCAN	1
VCL	1
VEZF1	1
VGF	1
VHL	1
VPS28	1
VPS35	1
VPS37A	1
VPS37D	1
VPS4B	1
VSIG10	1
VSIG10L	1
VSTM2B	1
WASF1	1
WASF2	1
WBP1L	1
WBP2	1

WBP4	1
WBSCR27	1
WDR26	1
WDR44	1
WDR45	1
WDR45B	1
WDR47	1
WDR5	1
WDR55	1
WDR76	1
WDR91	1
WEE1	1
WIF1	1
WIPF1	1
WIPF3	1
WISP1	1
WIZ	1
WNT10A	1
WNT3	1
WNT4	1
WNT7B	1
WNT8B	1
WSB1	1
WSB2	1
WSCD1	1
WSCD2	1
WT1	1
WTIP	1
WWC1	1
WWP1	1
XBP1	1
XPO4	1
XPO7	1
YIF1A	1
YLPM1	1
YTHDC2	1
YWHAB	1
ZBED3	1
ZBTB11	1
ZBTB14	1
ZBTB34	1
ZBTB40	1
ZBTB43	1
ZBTB44	1
ZBTB46	1
ZBTB47	1
ZBTB6	1
ZC2HC1C	1
ZC3H15	1
ZC3H8	1
ZCCHC24	1
ZDHHC14	1
ZDHHC18	1
ZDHHC2	1
ZDHHC23	1
ZEB1	1
ZFAND4	1
ZFC3H1	1
ZFHX3	1
ZFHX4	1
ZFP1	1
ZFP14	1
ZFP3	1
ZFP69B	1
ZFP82	1
ZFX	1
ZFYVE9	1
ZHX1	1
ZHX2	1
ZIC5	1
ZKSCAN1	1
ZMPSTE24	1
ZNF136	1
ZNF189	1
ZNF23	1
ZNF236	1

ZNF266	1
ZNF283	1
ZNF33B	1
ZNF367	1
ZNF394	1
ZNF395	1
ZNF398	1
ZNF445	1
ZNF469	1
ZNF470	1
ZNF496	1
ZNF527	1
ZNF536	1
ZNF562	1
ZNF572	1
ZNF597	1
ZNF608	1
ZNF613	1
ZNF699	1
ZNF706	1
ZNF711	1
ZNF780B	1
ZNF787	1
ZNF804A	1
ZNRF1	1
ZNRF3	1
ZRANB2	1
ZSCAN12	1
ZSWIM1	1
ZXDA	1
ZXDB	1
ZZEF1	1
ZZZ3	1

Supplementary Table 3. Predicted target genes of all detected miRNAs from Table 3.

Target Gene	Number of miRNAs
ZBTB20	12
QKI	11
DCP2	10
SZRD1	10
CADM1	7
KMT2A	7
NFAT5	7
RPS6KA5	7
ADAMTS5	6
AGO2	6
CSF1	6
MTF1	6
MTMR9	6
NSD1	6
NUDT3	6
SLC5A3	6
TXNIP	6
UBN2	6
ZNF618	6
C16orf52	5
C18orf25	5
CBX5	5
CFL2	5
CPEB4	5
DCUN1D4	5
ITGA5	5
ITGA9	5
KAT7	5
KCND3	5
KIAA0232	5
MGAT4A	5
MITF	5
MSI2	5
MYO9A	5
ONECUT2	5
PDE4D	5
PNRC2	5
ROBO1	5
RSBN1L	5
RYBP	5
SERBP1	5
SFMBT1	5
TEAD1	5
TGIF2	5
TMED7	5
TNPO1	5
UBE2D3	5
ZFHX3	5
ABCA1	4
ACVR2B	4
AGO1	4
AGO3	4
ARHGAP21	4
ARRDC3	4
ATP11A	4
ATP6AP2	4
ATP8A1	4
B4GALT5	4
BAZ2A	4
BAZ2B	4
BCL2L11	4
BHLHE41	4
C5orf30	4
C7orf60	4
CBLB	4

CDK19	4
CDK5R1	4
CLCN3	4
CNNM2	4
DYNLL2	4
DYRK1A	4
E2F3	4
EFNB2	4
ELAVL4	4
FBXO28	4
FIGN	4
FRYL	4
GDF6	4
GRID2	4
GRIN2B	4
GRIN2D	4
HLA-DQB2	4
HOMER1	4
HSPA4L	4
INO80	4
JHDM1D	4
JMJD1C	4
KCNJ6	4
KCNN3	4
KCNQ4	4
KLF6	4
LCOR	4
LEPROTL1	4
LIN28B	4
MAP3K2	4
MAPK1	4
MARCH3	4
MBOAT2	4
MDGA2	4
MDM4	4

MED12L	4
MEOX2	4
MIER1	4
MOB1B	4
MXD1	4
NAA15	4
NCAM1	4
NOVA1	4
NPTN	4
OSBPL11	4
PITPNM2	4
PPARGC1A	4
PPP1R10	4
PTEN	4
RAB14	4
RBM24	4
RC3H1	4
RICTOR	4
RMND5A	4
RNF38	4
ROCK1	4
S1PR1	4
SIRT7	4
SLC24A4	4
SLC7A11	4
SMAD2	4
SMURF2	4
SNN	4
SOS2	4
SOX11	4
SPTY2D1	4
SS18	4
SYT1	4
TAF4	4
TCF4	4

TNRC6A	4
TNRC6C	4
UBE2W	4
UHMK1	4
WDR47	4
WNT1	4
ZC3H12C	4
ZDHHC7	4
ABL2	3
AC140061.12	3
ACHE	3
ACVR1	3
ADAM10	3
ADAM22	3
ADCY2	3
AFF3	3
AGFG1	3
AKIRIN1	3
ANK2	3
ANKFY1	3
ARF4	3
ARFGEF1	3
ARID3A	3
ARL8B	3
ATXN3	3
BBX	3
BICC1	3
BMP3	3
BNIP2	3
BRWD3	3
C15orf27	3
C16orf70	3
CACNG2	3
CAND1	3
CCDC6	3

CCDC71	3
CCNK	3
CHD1	3
CHD9	3
CHST1	3
CLCN5	3
CLCN6	3
CNOT6	3
CNOT7	3
CNR1	3
CPD	3
CSNK2A1	3
DCP1A	3
DDAH1	3
DDX6	3
DLG2	3
DLL4	3
DPYSL5	3
DSTYK	3
DTX4	3
DUSP1	3
E2F7	3
EGR3	3
EIF4E3	3
ELAVL2	3
ELMO1	3
EPB41L1	3
F3	3
FAM184A	3
FBXO33	3
FBXW7	3
FCHSD1	3
FMR1	3
FOSB	3
FOXF1	3

FUBP1	3		MPP2	3
FUT9	3		MPPED2	3
FXR1	3		MTSS1L	3
GAB1	3		MYBL1	3
GGA2	3		NCOA1	3
GMFB	3		NCS1	3
GRIN2A	3		NEURL1B	3
H2AFY	3		NEURL4	3
HBEGF	3		NHS	3
HIPK3	3		NOL4	3
HMGB3	3		NPTX1	3
IGF1	3		NRARP	3
IL6ST	3		NRP1	3
ITSN2	3		OTUD4	3
KDM2B	3		OTX2	3
KDM5A	3		PAIP2	3
KIAA1045	3		PAN3	3
KIAA1468	3		PHF20	3
KIAA1549	3		PHF6	3
KLF4	3		PIK3C2A	3
KLHL18	3		PIK3CA	3
KMT2C	3		PLAA	3
KPNA1	3		PLK2	3
KSR1	3		PPAP2B	3
LDLR	3		PPFIA2	3
LIN28A	3		PPP1R9A	3
LMTK2	3		PSEN1	3
LYSMD3	3		PTPRD	3
MAFG	3		RAB24	3
MAP3K4	3		RAB39B	3
MED1	3		RAI14	3
MET	3		RARA	3
MLLT10	3		RLIM	3
MMD	3		RNPEPL1	3
MNT	3		RORA	3

RORB	3
RXRA	3
SATB1	3
SBNO1	3
SGMS1	3
SH3PXD2A	3
SIK1	3
SLC35F1	3
SLC6A1	3
SLC7A5	3
SLITRK3	3
SMAD5	3
SMIM17	3
SNAP91	3
SOBP	3
SOCS5	3
SORL1	3
SOS1	3
SOX5	3
SPRY3	3
SRPK2	3
STK38L	3
STOX2	3
SYT2	3
TBPL1	3
TERF2	3
TFDP2	3
TFRC	3
TMEM170A	3
TMSB10	3
TNRC6B	3
TRIP12	3
TRPS1	3
TRPV3	3
TXLNG	3

UBE2D1	3
UGCG	3
UGT8	3
VANGL1	3
VASH2	3
WAPAL	3
YPEL3	3
YWHAB	3
ZADH2	3
ZBTB37	3
ZBTB7A	3
ZCCHC2	3
ZNF704	3
ZNF784	3
ABCB10	2
ABCB7	2
ABCD3	2
AC012215.1	2
AC068987.1	2
AC135178.1	2
ACVR2A	2
ADAM17	2
ADAMTS15	2
ADAMTS18	2
ADAMTS19	2
ADCY6	2
AFF4	2
AGO4	2
AHDC1	2
AJUBA	2
AKAP1	2
ALCAM	2
ALG9	2
AMER1	2
AMER2	2

AMMECR1	2
ANKRD13A	2
ANKRD52	2
AP1G1	2
AP4E1	2
APPBP2	2
AQP11	2
ARAP2	2
ARF3	2
ARFIP1	2
ARHGAP12	2
ARHGAP20	2
ARHGEF12	2
ARHGEF17	2
ARID2	2
ARL6IP1	2
ARPP19	2
ARRDC4	2
ASAP1	2
ASPH	2
ATG14	2
ATG4D	2
ATP2A2	2
ATP2B4	2
ATP7A	2
ATXN1	2
ATXN7L1	2
B4GALT2	2
B4GALT6	2
BACH2	2
BAI3	2
BARHL2	2
BCL2L13	2
BCL2L2	2
BCL7A	2

BCL7B	2
BEND4	2
BEST1	2
BMP2K	2
BRPF1	2
BTAF1	2
BTBD3	2
BTBD7	2
BTG2	2
BZRAP1	2
C11orf87	2
C12orf79	2
C17orf85	2
C1GALT1	2
C1orf21	2
C1orf35	2
C1orf50	2
C20orf112	2
C2CD2	2
C2orf91	2
C3orf58	2
C5orf24	2
C6orf222	2
C6orf62	2
C7orf71	2
CA10	2
CABLES2	2
CABP7	2
CALR	2
CAMK2A	2
CAMKK1	2
CAMSAP2	2
CANX	2
CAPRIN1	2
CAST	2

CASZ1	2
CAV2	2
CCDC144A	2
CCDC85A	2
CCDC85C	2
CCNA2	2
CCT6A	2
CDC14A	2
CDC25B	2
CDH20	2
CDH7	2
CDK13	2
CDK14	2
CDK6	2
CDK8	2
CDKN1B	2
CDS1	2
CEBPG	2
CECR6	2
CEP55	2
CEP63	2
CEP72	2
CERS5	2
CERS6	2
CHD7	2
CHMP4B	2
CHRNB2	2
CLOCK	2
CLSTN2	2
CLVS2	2
CNIH4	2
CNTN4	2
COG5	2
COL2A1	2
COL4A1	2
COL6A3	2
CPEB3	2
CPT1A	2
CS	2
CTD-2140B24.4	2
CTDSP2	2
CTDSPL	2
CTDSPL2	2
CTSA	2
CTTNBP2NL	2
CUL5	2
CXorf23	2
CYB561D1	2
CYB5R4	2
CYTH3	2
DCAF7	2
DCBLD2	2
DCUN1D3	2
DEDD	2
DENND4C	2
DENND5B	2
DEPDC1B	2
DGCR8	2
DHRS13	2
DICER1	2
DLL1	2
DMPK	2
DMRTA2	2
DMXL1	2
DNAJB12	2
DNAJB4	2
DNAJC16	2
DNAJC18	2
DNMT1	2
DNMT3A	2

DOCK11	2
DOCK6	2
DOT1L	2
DPP9	2
DRGX	2
DSC2	2
DUSP16	2
DUSP8	2
DYRK1B	2
DYRK2	2
EDNRA	2
EFR3A	2
EGFR	2
EHF	2
EML6	2
EMX2	2
ENAH	2
ENTPD5	2
EOGT	2
EPAS1	2
EPC2	2
EPHA8	2
EPN2	2
EPS15	2
ERBB3	2
ERLIN1	2
ERRFI1	2
ESCO1	2
ESR1	2
ESRRG	2
ETNK1	2
ETV1	2
ETV3	2
ETV5	2
EXTL3	2

EYA3	2
EYA4	2
FAM104A	2
FAM104B	2
FAM105B	2
FAM133A	2
FAM133B	2
FAM134C	2
FAM161A	2
FAM168B	2
FAM21A	2
FAM21B	2
FAM21C	2
FAM21D	2
FAM43A	2
FAM46A	2
FAM78A	2
FAM81A	2
FAM84B	2
FANCA	2
FBN1	2
FBN3	2
FBXL19	2
FBXO11	2
FEZ2	2
FGF2	2
FLJ00418	2
FLOT2	2
FMNL3	2
FNBP1L	2
FOXK2	2
FOXP2	2
FRMPD3	2
GABRA1	2
GABRB1	2

GADD45A	2
GAP43	2
GAPVD1	2
GAS1	2
GATAD2A	2
GATAD2B	2
GJD2	2
GLRX5	2
GNA13	2
GPAM	2
GPATCH2	2
GPATCH8	2
GPCPD1	2
GPM6A	2
GPR116	2
GPR137C	2
GPR180	2
GPRIN2	2
GRAMD1B	2
GREM1	2
GRIK3	2
GSPT1	2
GSR	2
GTF2H1	2
HDGF	2
HECW2	2
HIF1AN	2
HLA-DQB1	2
HLF	2
HMGA2	2
HOXA5	2
HOXC8	2
HSP90B1	2
IL15	2
IL18BP	2

ING2	2
INHBB	2
INSIG2	2
IPO5	2
ISM1	2
ITFG3	2
ITGA11	2
ITGB8	2
ITPK1	2
JARID2	2
JMY	2
JPH3	2
KANSL1	2
KCMF1	2
KCNH1	2
KCNK10	2
KCNK2	2
KDM6B	2
KIAA0226	2
KIAA1217	2
KIAA1239	2
KIAA1324L	2
KIT	2
KITLG	2
KLC2	2
KLF5	2
KLHL5	2
KPNA4	2
LAMA4	2
LBR	2
LGALS8	2
LHX1	2
LIMD2	2
LIPA	2
LMAN1	2

LNPEP	2
LONRF3	2
LRCH1	2
LRFN1	2
LRP4	2
LRP8	2
LRRC41	2
LSAMP	2
LTBP1	2
LUC7L3	2
LYRM2	2
LYSMD2	2
MAF1	2
MAF	2
MAFB	2
MAP1B	2
MAP2K1	2
MAP3K9	2
MAP4K2	2
MARCH2	2
MAX	2
MBD5	2
MCM3AP	2
MDFIC	2
MECP2	2
MEIS2	2
MGAT5	2
MGLL	2
MIER3	2
MIPOL1	2
MLEC	2
MLLT6	2
MMP15	2
MMP19	2
MMS19	2

MOCOS	2
MOGS	2
MOSPD1	2
MRAS	2
MRPS10	2
MRPS25	2
MTA2	2
MTMR10	2
MTMR1	2
MTMR12	2
MTMR14	2
MUM1L1	2
MVB12B	2
MYRF	2
NAA50	2
NACC2	2
NAP1L1	2
NAT14	2
NAT8L	2
NCEH1	2
NCKAP1	2
NCKIPSD	2
NCOR1	2
NDP	2
NDST1	2
NETO1	2
NEUROD1	2
NFE2L1	2
NFIA	2
NFIB	2
NFIX	2
NFYA	2
NLK	2
NOG	2
NOVA2	2

NPEPL1	2
NR2C2AP	2
NR2E1	2
NR2F2	2
NRAS	2
NRD1	2
NRK	2
NSL1	2
NT5C1A	2
NUFIP2	2
NUP43	2
ORMDL1	2
OSBPL10	2
OSTF1	2
OXSR1	2
PAG1	2
PAPD4	2
PAQR9	2
PATL1	2
PBXIP1	2
PCYT1B	2
PCYT2	2
PDE4B	2
PDE5A	2
PDE7A	2
PDIA3	2
PDK4	2
PDPK1	2
PELI3	2
PGM2L1	2
PHACTR2	2
PHAX	2
PHF3	2
PHIP	2
PI4KA	2

PICALM	2
PIGA	2
PIGG	2
PIK3R3	2
PLXNC1	2
PNPLA2	2
PNPLA6	2
PODXL	2
POU3F2	2
POU4F2	2
PPARD	2
PPARGC1B	2
PPM1E	2
PPP1CB	2
PPP1R12A	2
PPP1R3B	2
PPP1R9B	2
PPP2R5E	2
PPP6R1	2
PRDM15	2
PRDM16	2
PREX1	2
PRICKLE2	2
PRKAA1	2
PRKAG2	2
PRKAR1A	2
PRKD1	2
PRNP	2
PRR15	2
PRR5L	2
PRRG1	2
PTCD3	2
PTGES3	2
PTPN14	2
PTPN5	2

PTPRA	2
PTPRM	2
PTPRT	2
PVRL1	2
PVRL4	2
RAB10	2
RAB11FIP1	2
RAB12	2
RAB34	2
RAB35	2
RALBP1	2
RALGAPA2	2
RALY	2
RAP1B	2
RAP2B	2
RASAL1	2
RASSF8	2
RBFOX2	2
RCAN2	2
RCC2	2
RFX3	2
RFX7	2
RGMA	2
RNF219	2
RNF44	2
ROBO2	2
RP11-1055B8.7	2
RP1-170O19.20	2
RPS6KB1	2
RPUSD1	2
RSBN1	2
RTN4	2
RUNX1T1	2
SALL4	2
SBF2	2

SCML2	2
SCN8A	2
SEC22A	2
SECISBP2L	2
SEMA6A	2
SERPINE1	2
SERTAD2	2
SESN2	2
SESN3	2
SESTD1	2
SET	2
SFXN2	2
SGCB	2
SH2B3	2
SH3BGRL2	2
SH3KBP1	2
SHH	2
SIX4	2
SKIDA1	2
SKP1	2
SLC10A7	2
SLC13A2	2
SLC16A6	2
SLC24A2	2
SLC25A44	2
SLC2A1	2
SLC2A14	2
SLC2A3	2
SLC30A9	2
SLC43A2	2
SLC45A3	2
SLC8A1	2
SLK	2
SMAD9	2
SMARCD1	2

SMIM12	2
SMPD3	2
SMS	2
SNAP25	2
SNPH	2
SNRK	2
SNX17	2
SNX2	2
SNX27	2
SNX3	2
SOCS3	2
SOGA2	2
SOX7	2
SP1	2
SPIN1	2
SPIRE1	2
SPRYD7	2
SREK1	2
SRGAP2	2
SRSF11	2
SSBP3	2
SSR1	2
ST18	2
ST6GALNAC3	2
ST8SIA3	2
STARD13	2
STAT1	2
STRADB	2
STT3A	2
STX11	2
STX3	2
STX6	2
STXBP5	2
SUGT1	2
SULF1	2

SUZ12	2
SYNCRIP	2
SYNJ1	2
TAF1D	2
TAF4B	2
TANC1	2
TANC2	2
TAOK1	2
TBC1D5	2
TBC1D8	2
TBC1D9B	2
TBL1XR1	2
TEK	2
TET2	2
TGFA	2
TGFB2	2
TGFBRAP1	2
TIGD5	2
TIMM8A	2
TMED5	2
TMEM11	2
TMEM127	2
TMEM164	2
TMEM52B	2
TMEM54	2
TMEM63B	2
TMEM9B	2
TMSB4X	2
TMX1	2
TNFRSF1B	2
TOMM70A	2
TRAK2	2
TRAPPC8	2
TRIM23	2
TSPAN18	2

TSPAN31	2
TSPYL1	2
TTC38	2
TUBB6	2
UBA6	2
UBAP2L	2
UBE2E2	2
UBE2K	2
UBE3B	2
UBE4B	2
UBR1	2
UBR5	2
UBXN1	2
UCP3	2
ULK3	2
UNC5D	2
UNC79	2
USP32	2
USP33	2
USP37	2
USP38	2
USP4	2
USP42	2
USP47	2
USP48	2
USP49	2
USP6	2
VAV2	2
VMP1	2
VSIG10	2
VSNL1	2
WASL	2
WDFY2	2
WDR20	2
WEE1	2
WHSC1	2
WNT10B	2
XPO4	2
YTHDC2	2
ZBED4	2
ZBTB16	2
ZBTB18	2
ZBTB34	2
ZBTB39	2
ZBTB8A	2
ZC3H12B	2
ZDHHC17	2
ZDHHC2	2
ZDHHC23	2
ZEB1	2
ZFHX4	2
ZFPM2	2
ZFYVE26	2
ZNF217	2
ZNF268	2
ZNF274	2
ZNF385A	2
ZNF395	2
ZNF652	2
ZNF804A	2
ZNF821	2
ZNRF1	2
ABCA12	1
ABCB9	1
ABHD12B	1
ABHD17B	1
ABHD5	1
ACBD3	1
ACOT11	1
ACTA2	1

ACVR1C	1
ADAM19	1
ADAMTS10	1
ADCY3	1
ADD2	1
ADORA2B	1
AFAP1	1
AFF1	1
AGPAT3	1
AGRN	1
AK2	1
AK4	1
AKT2	1
AL626787.1	1
ALDH1L2	1
ALDH4A1	1
AMD1	1
AMMECR1L	1
AP3D1	1
AP5M1	1
APBA2	1
APEX1	1
APLNR	1
ARHGAP19-SLIT1	1
ARHGAP24	1
ARHGAP32	1
ARHGAP5	1
ARHGEF11	1
ARHGEF37	1
ARID1B	1
ARID4A	1
ARID4B	1
ARMC8	1
ARX	1
ATAD2B	1

ATL3	1
ATP10B	1
ATP11B	1
ATP2B1	1
ATP5G3	1
ATP6V1F	1
ATP6V1H	1
ATP9A	1
ATXN10	1
AXIN1	1
B3GNT7	1
BACE1	1
BAG2	1
BAG4	1
BAHD1	1
BAK1	1
BCKDHA	1
BCL3	1
BCLAF1	1
BCORL1	1
BEND3	1
BIRC6	1
BLOC1S5	1
BMI1	1
BMPR2	1
BRMS1L	1
BRPF3	1
BRSK1	1
BRSK2	1
BTF3L4	1
BZW1	1
C10orf137	1
C10orf53	1
C11orf84	1
C12orf23	1

C15orf61	1
C17orf70	1
C19orf40	1
C19orf81	1
C1orf52	1
C20orf194	1
C21orf91	1
C3orf14	1
C6orf211	1
C6orf223	1
C7orf41	1
C7orf76	1
C8orf4	1
CA12	1
CA5B	1
CA7	1
CABLES1	1
CABP1	1
CACNA1A	1
CACNA2D3	1
CACYBP	1
CALN1	1
CAMK2G	1
CAPN7	1
CAPZA1	1
CASC10	1
CASC3	1
CASK	1
CASP8	1
CBFA2T3	1
CBFB	1
CCDC120	1
CCDC149	1
CCDC28B	1
CCDC50	1

CCDC88A	1
CCDC88C	1
CCDC92	1
CCM2	1
CCNG1	1
CCNJ	1
CCNT2	1
CCNY	1
CCR10	1
CCT3	1
CD2AP	1
CDC37L1	1
CDC6	1
CDH11	1
CDH2	1
CDH24	1
CDH5	1
CDIP1	1
CDK12	1
CDK18	1
CDON	1
CDR2L	1
CDS2	1
CDYL2	1
CECR2	1
CELSR1	1
CENPP	1
CEP135	1
CEP170	1
CEP350	1
CEP57	1
CHD4	1
CHD6	1
CHD8	1
CHIC1	1

CHRM2	1
CHTF8	1
CISD2	1
CITED2	1
CKAP4	1
CLASP1	1
CLLU1	1
CLP1	1
CLPP	1
CNKSR3	1
CNN3	1
CNOT1	1
CNTN2	1
CNTNAP2	1
COL11A2	1
COL21A1	1
COL26A1	1
COL27A1	1
COL3A1	1
COL4A3BP	1
COLCA1	1
COLGALT2	1
COLQ	1
COMMD3-BMI1	1
CORO1C	1
COX7B	1
CPNE2	1
CREB1	1
CREBRF	1
CRK	1
CRKL	1
CRP	1
CRTC1	1
CSDC2	1
CSE1L	1

CSNK1D	1
CSRNP1	1
CSRP2	1
CTAGE5	1
CTC-435M10.3	1
CTH	1
CUL4B	1
CYP39A1	1
DAAM1	1
DAB1	1
DAB2IP	1
DACH1	1
DAZAP2	1
DCAF17	1
DCC	1
DCUN1D1	1
DCUN1D5	1
DCX	1
DDB1	1
DDHD1	1
DDI2	1
DGKE	1
DGKH	1
DGKI	1
DHX15	1
DIDO1	1
DIRAS1	1
DIRAS2	1
DKK2	1
DLGAP3	1
DLK1	1
DMBX1	1
DMD	1
DNAJB1	1
DNAJC13	1

DNAJC3	1
DONSON	1
DPY19L3	1
DR1	1
DROSHA	1
DRP2	1
DST	1
DTNA	1
DTX1	1
DUSP10	1
DUSP13	1
DUSP18	1
DUSP5	1
DUSP9	1
DVL2	1
E2F6	1
EBF3	1
EBF4	1
ECE1	1
EDAR	1
EDEM3	1
EDIL3	1
EEPD1	1
EFNA2	1
EHD3	1
EIF2B3	1
EIF2S2	1
EIF4G2	1
EIF5	1
ELF2	1
ELFN2	1
ELL	1
ELL2	1
ELMOD1	1
ELMSAN1	1

ELOVL3	1
ELOVL4	1
ELOVL6	1
ELP6	1
EML1	1
EN1	1
EN2	1
ENOX1	1
EPB41	1
EPB41L2	1
EPB41L4A	1
EPHA5	1
EPHB2	1
ERBB4	1
ERC2	1
ERG	1
ERGIC2	1
ERMP1	1
ERP44	1
EVX2	1
EXOC5	1
EYA1	1
FADS1	1
FAM102A	1
FAM110B	1
FAM126A	1
FAM126B	1
FAM129A	1
FAM13A	1
FAM155A	1
FAM155B	1
FAM160B1	1
FAM167A	1
FAM172A	1
FAM175A	1

FAM177A1	1
FAM192A	1
FAM193B	1
FAM222A	1
FAM222B	1
FAM53C	1
FAM63B	1
FAM65B	1
FAM76A	1
FAM83F	1
FAXC	1
FBLN2	1
FBLN5	1
FBRS	1
FBRSL1	1
FBXL20	1
FBXL4	1
FBXO21	1
FBXO30	1
FEM1B	1
FERMT2	1
FGD6	1
FGF14	1
FKBP14	1
FKBP6	1
FLJ20373	1
FLRT2	1
FLRT3	1
FNBP1	1
FNDC4	1
FOSL2	1
FOXA1	1
FOXB1	1
FOXD3	1
FOXN3	1

FOXN4	1
FOXO4	1
FOXP4	1
FOXQ1	1
FRMD4A	1
FUBP3	1
FURIN	1
FXR2	1
FYTTD1	1
FZD3	1
FZD4	1
FZD5	1
G6PC3	1
GABBR2	1
GAD1	1
GALNT3	1
GALNT5	1
GALNT7	1
GAREM	1
GATA2	1
GATA6	1
GBX2	1
GCC1	1
GCC2	1
GCNT2	1
GDF11	1
GFI1	1
GFPT1	1
GFPT2	1
GHR	1
GIGYF1	1
GIPC1	1
GJA3	1
GJB1	1
GLIS3	1

GLRA2	1		GREB1L	1
GLTP	1		GRHL2	1
GLUD2	1		GRHL3	1
GMEB2	1		GRIA1	1
GMPS	1		GRM5	1
GNB5	1		GSK3B	1
GNG12	1		GSPT2	1
GNG2	1		GTDC1	1
GNS	1		GTF2A2	1
GOLGA6A	1		GXYLT1	1
GOLGA6B	1		GYG1	1
GOLGA6C	1		H3F3B	1
GOLGA6D	1		H3F3C	1
GOLGA6L10	1		HAND2	1
GOLGA6L4	1		HAO1	1
GOLGA8A	1		HAPLN1	1
GOLM1	1		HCN4	1
GORASP1	1		HDAC4	1
GOT2	1		HECTD1	1
GPATCH2L	1		HEG1	1
GPBP1	1		HGF	1
GPC4	1		HHIP	1
GPD2	1		HIAT1	1
GPR125	1		HIC1	1
GPR126	1		HIF1A	1
GPR133	1		HIP1	1
GPR135	1		HIST1H2BD	1
GPR174	1		HIVEP3	1
GPR183	1		HLX	1
GPR61	1		HMG20A	1
GPR63	1		HMX3	1
GPR75	1		HNF1B	1
GPRIN3	1		HNF4G	1
GRAMD2	1		HNRNPA0	1
GRB2	1		HNRNPF	1

HOMEZ	1
HOOK3	1
HOXA10	1
HOXA13	1
HOXA9	1
HOXB8	1
HOXC6	1
HS3ST2	1
HSD17B8	1
HSPD1	1
HTT	1
HUNK	1
ID3	1
ID4	1
IFFO2	1
IFT122	1
IGF1R	1
IGJ	1
IGLON5	1
IGSF3	1
IKZF1	1
IKZF2	1
IKZF5	1
ILDR2	1
ING5	1
INO80D	1
INSM1	1
INSR	1
IPMK	1
IPO7	1
IRS1	1
IRS4	1
ISL1	1
ITGA2	1
ITGA8	1
ITGB6	1
ITPKB	1
ITPKC	1
ITSN1	1
JAG1	1
KAT6A	1
KBTBD11	1
KBTBD8	1
KCNA1	1
KCNA4	1
KCNA6	1
KCNAB1	1
KCND2	1
KCNJ11	1
KCNJ13	1
KCNJ3	1
KCNK12	1
KCNMB4	1
KCTD16	1
KDM3A	1
KDM5B	1
KHDRBS2	1
KIAA0087	1
KIAA1033	1
KIAA1109	1
KIAA1147	1
KIAA1199	1
KIAA1549L	1
KIAA1551	1
KIF3A	1
KIRREL	1
KLF3	1
KLF7	1
KLHDC7A	1
KLHDC8A	1

KLHL31	1
KPNA3	1
KPNA5	1
KPNA6	1
KPNB1	1
KRR1	1
LANCL3	1
LAPTM4B	1
LASP1	1
LBH	1
LCMT2	1
LDLRAD3	1
LEP	1
LHX6	1
LIF	1
LIFR	1
LIMCH1	1
LIMK1	1
LIMK2	1
LIN52	1
LIN7A	1
LIN7C	1
LMBR1L	1
LMNB1	1
LMX1B	1
LONRF1	1
LOX	1
LPCAT1	1
LPHN2	1
LPIN2	1
LPPR1	1
LPPR5	1
LRAT	1
LRP6	1
LRRC4C	1

LRRFIP1	1
LSM12	1
LYPD3	1
LZTS3	1
MAGI3	1
MAGT1	1
MAN2A1	1
MAN2A2	1
MANEAL	1
MAP2	1
MAP2K7	1
MAP3K3	1
MAP4K5	1
MAP7D3	1
MAPK10	1
MAPK14	1
MAPK6	1
MAPK8IP3	1
MAPKAPK3	1
MAPRE2	1
MARCH5	1
MARCH8	1
MARCKS	1
MATN3	1
MATR3	1
MBLAC2	1
MBNL2	1
MBTD1	1
MCF2L	1
MCM10	1
MCTS1	1
MDFI	1
MDGA1	1
MDN1	1
MED13	1

MED13L	1
MED14	1
MEF2C	1
MEGF11	1
MEPCE	1
MESDC1	1
METTL21A	1
MEX3A	1
MFAP2	1
MFHAS1	1
MGAT1	1
MGAT4B	1
MIA3	1
MIB1	1
MICALCL	1
MICU3	1
MID1IP1	1
MIEF1	1
MIER2	1
MINPP1	1
MKNK2	1
MME	1
MN1	1
MOSPD3	1
MPP6	1
MPPED1	1
MSANTD3-TMEFF1	1
MSI1	1
MSL1	1
MTDH	1
MTMR4	1
MUC19	1
MXI1	1
MYH10	1
MYO5A	1
MYOCD	1
MYRIP	1
MYT1	1
N4BP1	1
NABP1	1
NAIF1	1
NAPEPLD	1
NAV2	1
NAV3	1
NBEAL1	1
NCAN	1
NCL	1
NCOA7	1
NDRG3	1
NDUFB9	1
NDUFS4	1
NEBL	1
NEDD4	1
NEK2	1
NEK6	1
NEO1	1
NETO2	1
NEUROD4	1
NEUROD6	1
NF1	1
NFASC	1
NFE2L2	1
NGFR	1
NGFRAP1	1
NIPAL4	1
NIPBL	1
NKX3-2	1
NKX6-1	1
NLN	1
NMRK1	1

NOP9	1
NPAS3	1
NPAT	1
NPEPPS	1
NPTX2	1
NPTXR	1
NR1D2	1
NR2F6	1
NR5A2	1
NR6A1	1
NRBF2	1
NREP	1
NRIP1	1
NRIP2	1
NRP2	1
NRXN1	1
NSUN3	1
NTN1	1
NUCKS1	1
NUDC	1
NUDT4	1
NUP210	1
NUS1	1
NXF1	1
NXPH1	1
NXT2	1
OAF	1
ONECUT3	1
OPA1	1
OPCML	1
ORC5	1
OSER1	1
OTP	1
OTUD7A	1
P2RY2	1

PACS1	1
PAFAH1B1	1
PAIP2B	1
PAK2	1
PALM2	1
PANK1	1
PAPD7	1
PAPOLG	1
PAPPA	1
PAPSS2	1
PARD6B	1
PAX2	1
PAX5	1
PAX6	1
PAX9	1
PCBD1	1
PCDH1	1
PCDH9	1
PCGF3	1
PCM1	1
PCNX	1
PDE10A	1
PDE1B	1
PDE3A	1
PDE3B	1
PDE7B	1
PDE8B	1
PDGFA	1
PDGFRA	1
PDHB	1
PDHX	1
PDIA5	1
PDK1	1
PDK3	1
PDLIM2	1

PDLIM4	1
PDP2	1
PDPN	1
PDS5B	1
PEAK1	1
PEG10	1
PELI1	1
PELI2	1
PER2	1
PFKM	1
PFN2	1
PGAP1	1
PGP	1
PHB	1
PHC2	1
PHF15	1
PHF21A	1
PHGDH	1
PHKA1	1
PHLDA1	1
PHLPP2	1
PIK3R1	1
PIP4K2B	1
PITHD1	1
PKIA	1
PKIB	1
PLAG1	1
PLAGL1	1
PLAGL2	1
PLCE1	1
PLCL2	1
PLCXD3	1
PLD5	1
PLEKHH1	1
PLEKHH2	1

PLEKHM1	1
PLXND1	1
PMEPA1	1
PMF1-BGLAP	1
PNISR	1
POFUT1	1
POGLUT1	1
POGZ	1
POM121C	1
POTEG	1
POTEM	1
POU2F1	1
POU2F3	1
POU3F3	1
PPARG	1
PPAT	1
PPFIBP1	1
PPIF	1
PPIG	1
PPME1	1
PPP1CC	1
PPP2R2C	1
PPP3R1	1
PPP4C	1
PPP4R1L	1
PPP4R2	1
PPTC7	1
PRCP	1
PRELID2	1
PRICKLE1	1
PRKX	1
PRMT8	1
PRPF19	1
PRR12	1
PRSS53	1

PSMC6	1
PSME3	1
PTBP2	1
PTCHD1	1
PTCHD3	1
PTCHD4	1
PTGDR	1
PTGER3	1
PTGFRN	1
PTGIS	1
PTPN12	1
PTPN4	1
PTPN9	1
PTPRB	1
PTRH2	1
PURA	1
PXN	1
RAB11FIP3	1
RAB13	1
RAB15	1
RAB20	1
RAB27A	1
RAB33B	1
RAB38	1
RAB3B	1
RAB4A	1
RAB6B	1
RAD51D	1
RAD51L3-RFFL	1
RAD54L2	1
RALGAPB	1
RALGPS1	1
RAPGEF2	1
RASA2	1
RASD2	1

RASGEF1B	1
RBBP4	1
RBM15B	1
RBP2	1
RBPMS2	1
RBX1	1
RCOR3	1
REEP2	1
RELN	1
REPS1	1
RERE	1
RET	1
REV3L	1
RFFL	1
RGL2	1
RGPD4	1
RGPD6	1
RGS17	1
RGS6	1
RHEB	1
RIMS3	1
RND3	1
RNF11	1
RNF141	1
RNF144A	1
RNF146	1
RNF182	1
RNGTT	1
ROCK2	1
ROR1	1
RP11-122A3.2	1
RP11-192H23.4	1
RP11-407N17.3	1
RP13-996F3.4	1
RP13-996F3.5	1

RPGRIP1L	1
RPL36A-HNRNPH2	1
RPN1	1
RPRD1B	1
RRAS	1
RREB1	1
RSPO3	1
RUFY3	1
RUNX1	1
RUNX3	1
SALL1	1
SAMD10	1
SAMD12	1
SAMD4A	1
SAMD9L	1
SAR1A	1
SASH1	1
SATB2	1
SAYSD1	1
SCAI	1
SCAMP1	1
SCAMP3	1
SCD	1
SCN1A	1
SCN3B	1
SCYL3	1
SDAD1	1
SDC4	1
SEC24A	1
SEC61A1	1
SEMA6D	1
SEMA7A	1
SEPHS1	1
SEPT11	1
SETD5	1

SETD7	1
SETD8	1
SFMBT2	1
SFPQ	1
SFRP1	1
SGCE	1
SGPP1	1
SH2D3C	1
SH3RF1	1
SHC3	1
SHE	1
SHISA6	1
SHOC2	1
SIK2	1
SIPA1L3	1
SIRT1	1
SIX1	1
SIX5	1
SKOR1	1
SLC12A6	1
SLC16A10	1
SLC16A1	1
SLC1A3	1
SLC22A23	1
SLC25A16	1
SLC25A25	1
SLC26A11	1
SLC29A4	1
SLC30A10	1
SLC30A1	1
SLC30A7	1
SLC30A8	1
SLC35F3	1
SLC38A4	1
SLC39A10	1

SLC39A11	1
SLC39A13	1
SLC40A1	1
SLC44A1	1
SLC6A6	1
SLC7A14	1
SLC9A7	1
SLCO3A1	1
SLIT1	1
SLIT2	1
SLTM	1
SMAP1	1
SMARCA2	1
SMG1	1
SMIM14	1
SMURF1	1
SNAI1	1
SNX1	1
SNX12	1
SNX18	1
SNX22	1
SNX29	1
SOCS2	1
SOCS6	1
SOWAHA	1
SOX17	1
SOX6	1
SP2	1
SP3	1
SP4	1
SPATA2	1
SPCS2	1
SPG21	1
SPOPL	1
SPPL3	1
SPRY2	1
SPTBN1	1
SPTLC2	1
SRCIN1	1
SRGAP1	1
SRI	1
SRL	1
SRM	1
SRP72	1
SRRD	1
SRSF1	1
SRSF9	1
SS18L1	1
ST8SIA4	1
STAB2	1
STAC	1
STAG1	1
STAT5B	1
STEAP3	1
STIM2	1
STK24	1
STK32B	1
STK35	1
STK39	1
STK40	1
STMN1	1
STRN4	1
STX16	1
SUCO	1
SUFU	1
SUMO2	1
SURF4	1
SV2A	1
SYDE1	1
SYNDIG1	1

SYNRG	1
SYTL3	1
SYVN1	1
TACC1	1
TAF3	1
TAL1	1
TAS2R5	1
TBC1D13	1
TBC1D22B	1
TBC1D25	1
TBC1D30	1
TBC1D4	1
TCEANC2	1
TCF7L2	1
TEFM	1
TET1	1
TEX2	1
TFEB	1
TGFBR1	1
TGFBR2	1
TGFBR3	1
TGIF1	1
TGOLN2	1
THAP1	1
THAP11	1
THAP2	1
THBS1	1
THOC1	1
THRB	1
TIAL1	1
TJAP1	1
TLK2	1
TLL1	1
TLN2	1
TLX1	1

TMBIM6	1
TMCC1	1
TMED2	1
TMED8	1
TMED9	1
TMEFF1	1
TMEM110	1
TMEM120B	1
TMEM123	1
TMEM132E	1
TMEM151B	1
TMEM167A	1
TMEM170B	1
TMEM178B	1
TMEM189	1
TMEM189-UBE2V1	1
TMEM194B	1
TMEM200C	1
TMEM229A	1
TMEM248	1
TMEM25	1
TMEM260	1
TMEM26	1
TMEM43	1
TMEM64	1
TMEM65	1
TMEM87A	1
TMEM91	1
TMTC2	1
TNIK	1
TNR	1
TNRC18	1
TOX4	1
TPD52L2	1
TRABD	1

TRAF3	1
TRAF3IP2	1
TRAF6	1
TRAPPC6B	1
TRERF1	1
TRHDE	1
TRIM44	1
TRIM67	1
TRIP4	1
TRMT2A	1
TROVE2	1
TSC1	1
TSC22D2	1
TSPAN14	1
TSPAN7	1
TSTD2	1
TTC28	1
TTC39A	1
TTC7B	1
TTC9	1
TTF2	1
TTLL7	1
TUB	1
TXNDC5	1
TYW1	1
UAP1	1
UBA2	1
UBE2E3	1
UBE2F	1
UBE2N	1
UBE2NL	1
UBE2V1	1
UBE2V2	1
UBXN7	1
ULK1	1

UNC13B	1
UNC13C	1
UNC80	1
UNKL	1
UPF1	1
URGCP	1
USP46	1
UST	1
VANGL2	1
VAPA	1
VAT1L	1
VAV3	1
VCAN	1
VCL	1
VEGFC	1
VGLL3	1
VPS4B	1
VWC2L	1
VWDE	1
WBP1L	1
WDR37	1
WDR7	1
WDTC1	1
WIPF2	1
WISP1	1
WNK1	1
WNK3	1
WNT3A	1
WSB1	1
XCR1	1
XPR1	1
YAP1	1
YPEL2	1
ZBTB10	1
ZBTB14	1

ZBTB3	1
ZBTB42	1
ZC3H12D	1
ZCCHC24	1
ZFAND5	1
ZFP36L1	1
ZFP36L2	1
ZFYVE9	1
ZHX1	1
ZIC1	1
ZKSCAN2	1
ZMAT3	1
ZMYM4	1
ZNF185	1
ZNF202	1
ZNF24	1
ZNF280C	1
ZNF385D	1
ZNF462	1
ZNF488	1
ZNF516	1
ZNF532	1
ZNF608	1
ZNF638	1
ZNF800	1
ZNF827	1
ZZZ3	1

Supplementary Table 4. Predicted target genes of miRNAs downregulated by RSV from Table 2 and 3.

Target Gene	miRNAs
PAX2	5
DYNLL2	4
KLF7	4
AAK1	3
BSN	3
CADM1	3
CALCOCO1	3
DPF2	3
FIGN	3
FOSL2	3
GPR61	3
LCOR	3
LPHN1	3
LRRC15	3
MED1	3
NFIX	3
NOX5	3
PDE7A	3
PRX	3
ST5	3
THRA	3
TRAF6	3
WWC2	3
ACKR2	2
AGO1	2
ANKFY1	2
ATG7	2
ATP1A3	2
BCL2L2	2
BEND3	2
CADM2	2
CBX5	2
CDKN2AIP	2
CECR6	2
CREB1	2
CSMD1	2
CSRNP3	2
DLGAP2	2
DNAH10OS	2
DNAJB2	2
ELAVL3	2
EPHA8	2
FAM76A	2
FBXO28	2
GATA6	2
GPR75	2
HIC2	2
HTT	2
IGF2R	2
IGSF1	2
IKZF3	2
IKZF4	2
IRAK1	2
KCND3	2
KIAA0141	2
KIAA0247	2
KLHDC10	2
KLHL29	2
KPNA5	2
LRRC8B	2
MDGA2	2

MDM4	2
MEX3A	2
MIEF1	2
MSI1	2
MTSS1L	2
MYBL1	2
MYO5A	2
NMNAT2	2
NOVA1	2
NR6A1	2
PBX2	2
PCDH10	2
PCDH1	2
POLR3H	2
PRKAA2	2
PTPRA	2
RIMS2	2
RORA	2
RORB	2
SETD9	2
SHANK3	2
SMTNL2	2
SNX22	2
SOBP	2
STRN	2
TANC2	2
TBC1D20	2
USP3	2
VPS52	2
VWC2L	2
XYLT1	2
ZBTB16	2
ZNF280C	2
ZNF367	2
ZNRF3	2
ABCA9	1
ABCC1	1
ABCG1	1
ABCG4	1
ABHD15	1
ABL2	1
AC012215.1	1
AC138655.1	1
ACER3	1
ACTG1	1
ACTN4	1
ADAM19	1
AFAP1L2	1
AFF4	1
AGO3	1
AGPAT3	1
AGPAT6	1
AHSA2	1
AKAP2	1
ALPL	1
AMIGO2	1
AMN	1
ANK1	1
ANK3	1
ANKRD12	1
ANKRD34A	1
ANXA7	1
APP	1
APPL1	1
AQPEP	1
ARHGAP19-SLIT1	1

ARHGEF12	1
ARIH2	1
ARMC8	1
ARNT	1
ARRDC3	1
ARSJ	1
ASB16	1
ASIC1	1
ATCAY	1
ATF2	1
ATP5J2-PTCD1	1
ATP6V1F	1
ATP8B1	1
ATXN2	1
ATXN7	1
BACH2	1
BAG1	1
BAI1	1
BAZ1B	1
BAZ2A	1
BAZ2B	1
BBX	1
BCL2L11	1
BCL2L14	1
BCL6	1
BCORL1	1
BCR	1
BDNF	1
BEND4	1
BICD1	1
BICD2	1
BLZF1	1
BMF	1

BMP2K	1
BMPR1A	1
BNC1	1
BRD4	1
BTG2	1
BTRC	1
C10orf76	1
C14orf28	1
C16orf52	1
C16orf72	1
C16orf89	1
C17orf75	1
C17orf78	1
C21orf33	1
C2CD2L	1
C3orf14	1
C4orf3	1
C5AR2	1
CA10	1
CABP4	1
CADM4	1
CALCR	1
CALN1	1
CAMK1G	1
CAMK2B	1
CAMK2D	1
CAMK2G	1
CAMSAP1	1
CAPZA1	1
CAPZB	1
CARD10	1
CASD1	1
CASK	1

CCDC6	1
CCDC71L	1
CCDC88A	1
CCDC9	1
CCND2	1
CCNJ	1
CCNK	1
CD3D	1
CD80	1
CDC14A	1
CDC42	1
CDC42EP3	1
CDC6	1
CDH24	1
CDH4	1
CDK6	1
CDON	1
CDS1	1
CELF1	1
CELF3	1
CELF6	1
CEP350	1
CEP85L	1
CHD6	1
CHL1	1
CHST11	1
CLASP2	1
CLCN5	1
CLCN6	1
CLDN18	1
CNKSR3	1
CNNM3	1
CNNM4	1

CNOT6	1
CNTD1	1
CNTFR	1
CNTN2	1
CNTNAP5	1
CORO2B	1
CPNE2	1
CRB3	1
CREBRF	1
CRK	1
CRLF3	1
CRNKL1	1
CSMD2	1
CSNK1D	1
CTA-299D3.8	1
CTD-2267D19.3	1
CTDSPL	1
CTDSPL2	1
CTNNBIP1	1
CTSE	1
CTSK	1
CXorf36	1
CYB561	1
CYP20A1	1
CYP4V2	1
CYTH1	1
DAB2IP	1
DAZAP1	1
DCAF12	1
DCLRE1C	1
DCTN3	1
DDHD1	1
DGCR14	1

DGKB	1
DGKG	1
DGKH	1
DGKI	1
DHDDS	1
DLEC1	1
DLG2	1
DLG5	1
DLGAP3	1
DNAH17	1
DNAH17-AS1	1
DNAJB14	1
DNAJC14	1
DNAL1	1
DNM1	1
DNPEP	1
DOCK11	1
DRP2	1
DTX3L	1
DUSP16	1
DVL3	1
DYRK1B	1
E2F3	1
E2F6	1
E2F7	1
EBF2	1
EDNRB	1
EHF	1
EIF4B	1
EIF4G2	1
EIF5A	1
EIF5A2	1
ELAVL2	1

ELK1	1
ELOVL2	1
ELOVL4	1
ELOVL6	1
ELP4	1
EMC10	1
ENAH	1
EPHA2	1
EPHA4	1
EPHA5	1
EPHB2	1
ERBB2	1
ERBB4	1
ERGIC2	1
ERI2	1
ERI3	1
ERLEC1	1
ESRRG	1
ESYT2	1
EVI5	1
F9	1
FADS3	1
FAIM2	1
FAM101A	1
FAM114A1	1
FAM134C	1
FAM168A	1
FAM169A	1
FAM196A	1
FAM208A	1
FAM210A	1
FAM222A	1
FAM26E	1

FAM53B	1
FAM53C	1
FAM65B	1
FAM83F	1
FAXC	1
FBXL20	1
FBXO30	1
FBXW2	1
FKBP15	1
FLJ20373	1
FLOT2	1
FLRT2	1
FLT1	1
FMO2	1
FNBP1L	1
FNDC5	1
FRK	1
FRS2	1
FRYL	1
FSTL4	1
FXR2	1
FZD1	1
FZD2	1
GABPA	1
GABRB1	1
GABRB2	1
GALM	1
GALNT10	1
GALNT1	1
GALNT8	1
GATA3	1
GATAD2A	1
GATAD2B	1

GATSL2	1
GCLM	1
GDNF	1
GGA2	1
GINM1	1
GINS2	1
GJA9	1
GLYATL3	1
GMPS	1
GNA13	1
GNL1	1
GOLGA3	1
GOSR1	1
GPBP1	1
GPCPD1	1
GPR107	1
GPR12	1
GPR156	1
GPR26	1
GPR37L1	1
GPRIN3	1
GPX8	1
GRID1	1
GRK5	1
GRSF1	1
GTF2H1	1
GTPBP1	1
H3F3B	1
H3F3C	1
HAS3	1
HAUS3	1
HCN1	1
HDAC4	1

HEYL	1
HIF3A	1
HIPK3	1
HNRNPA0	1
HNRNPD	1
HNRNPK	1
HOMEZ	1
HOOK3	1
HOXA1	1
HOXA13	1
HOXA3	1
HOXB3	1
HOXB6	1
HOXD10	1
HP1BP3	1
HSD17B8	1
HSPA4L	1
HSPH1	1
IFFO2	1
IFRG15	1
IGDCC4	1
IGJ	1
IL1RAPL1	1
IMPA2	1
INHBB	1
INO80D	1
INPP5K	1
IRS1	1
IST1	1
ITGB8	1
ITSN1	1
JARID2	1
JAZF1	1

JOSD1	1
KAT6B	1
KAT7	1
KBTBD4	1
KCMF1	1
KCNA6	1
KCNAB3	1
KCNC3	1
KCNH5	1
KCNJ6	1
KCNK10	1
KCNN3	1
KCTD15	1
KCTD16	1
KCTD17	1
KDM2A	1
KDM2B	1
KIAA1024	1
KIAA1199	1
KIAA1429	1
KIAA1462	1
KIAA1549	1
KIF1B	1
KIF24	1
KIF26B	1
KIF3C	1
KLC1	1
KLC2	1
KLF11	1
KLHL3	1
KLK5	1
KMT2D	1
KPNA6	1

KPNB1	1
L3MBTL3	1
LANCL1	1
LAYN	1
LCP2	1
LEPRE1	1
LFNG	1
LGALSL	1
LHFPL4	1
LIF	1
LIN28A	1
LIX1L	1
LPCAT3	1
LRCH3	1
LRRC16B	1
LRRC8E	1
LRRFIP1	1
LSAMP	1
LSM11	1
LTB	1
LYVE1	1
LZIC	1
MAN1C1	1
MANEAL	1
MAP3K13	1
MAP3K2	1
MAP3K7	1
MAPK14	1
MAPKBP1	1
MAPRE1	1
MAPRE2	1
MAPT	1
MARCH6	1

MARK1	1
MAT2A	1
MBD5	1
MCMBP	1
MESDC1	1
MFHAS1	1
MID2	1
MLLT3	1
MLLT6	1
MMP16	1
MORN4	1
MRRF	1
MTDH	1
MTF1	1
MTF2	1
MTMR3	1
MTMR9	1
MYCBP	1
MYLK	1
MYLK3	1
MYO18A	1
MYO6	1
MYPN	1
MYT1	1
MYT1L	1
NACC1	1
NAGS	1
NAIF1	1
NAV1	1
NCOA2	1
NCOA6	1
NCOR2	1
NDST1	1

NEDD4	1
NF2	1
NFASC	1
NFAT5	1
NFATC2	1
NFATC3	1
NFE2L1	1
NLRP1	1
NONO	1
NPAS3	1
NPAS4	1
NR1D1	1
NR2C2	1
NR4A3	1
NR5A2	1
NRAS	1
NRP2	1
NRSN1	1
NSD1	1
NT5C1A	1
NT5DC1	1
NTPCR	1
NUCKS1	1
NUDT17	1
NUFIP2	1
NUMB	1
NUP37	1
NUP50	1
ONECUT1	1
ONECUT2	1
OSBP2	1
OSTN	1
OTOP3	1

OTUD7A	1
PADI2	1
PAK6	1
PAK7	1
PALM2	1
PALM2-AKAP2	1
PAPD5	1
PAPOLA	1
PARPBP	1
PARS2	1
PAX5	1
PAX7	1
PAX8	1
PBX1	1
PCDHA10	1
PCDHA1	1
PCDHA11	1
PCDHA12	1
PCDHA13	1
PCDHA2	1
PCDHA3	1
PCDHA4	1
PCDHA5	1
PCDHA6	1
PCDHA7	1
PCDHA8	1
PCDHA9	1
PCDHAC1	1
PCDHAC2	1
PCP4L1	1
PDE12	1
PDE4A	1
PDF	1

PDHB	1
PDZD8	1
PEA15	1
PER1	1
PEX5L	1
PGK1	1
PHF7	1
PHLDB1	1
PHLPP2	1
PHOX2B	1
PHYHIP	1
PIK3CA	1
PIK3CB	1
PILRA	1
PIP5K1B	1
PITPNA	1
PKIA	1
PLA2G2F	1
PLAC8	1
PLEKHA1	1
PLEKHG3	1
PM20D2	1
PNKP	1
POC1A	1
POLH	1
POU3F2	1
PPAP2B	1
PPARGC1B	1
PPIP5K2	1
PPM1L	1
PPP1R11	1
PPP1R3B	1
PPP1R3E	1

PQLC1	1
PRCP	1
PRELID2	1
PRIM2	1
PRKAR2A	1
PRR15L	1
PRR26	1
PRRT2	1
PRRT3	1
PSMA4	1
PSMD11	1
PTCD1	1
PTCHD1	1
PTEN	1
PTGFRN	1
PTPN4	1
PTPRG	1
PURB	1
PURG	1
QKI	1
RAB14	1
RAB35	1
RABGAP1	1
RALGPS2	1
RAP2A	1
RARB	1
RASAL2	1
RASL10B	1
RB1CC1	1
RBM27	1
RBMS3	1
RCAN1	1
RCSD1	1

REL	1
RERE	1
RFTN2	1
RFX7	1
RHOBTB2	1
RIC8B	1
RIMS4	1
RNASEL	1
RNF165	1
RNF180	1
RNF186	1
RNF20	1
RNF26	1
RNF32	1
RNF4	1
ROBO1	1
ROCK2	1
RP11-156E8.1	1
RP11-343C2.12	1
RP11-73M18.2	1
RP11-766F14.2	1
RP6-24A23.6	1
RPA3	1
RPAP2	1
RPRD1A	1
RRAGD	1
RSBN1	1
RUNX1T1	1
RYBP	1
S1PR1	1
SAMD12	1
SAMD14	1
SAMD8	1

SAP30L	1
SAR1A	1
SBSPON	1
SCARB1	1
SCARB2	1
SCN1A	1
SCN3A	1
SCN3B	1
SDC1	1
SDHC	1
SEC22A	1
SEC23IP	1
SEL1L3	1
SEMA3G	1
SERPINE1	1
SERTAD4	1
SESN3	1
SETD5	1
SF1	1
SGIP1	1
SGMS1	1
SH3D19	1
SH3GL2	1
SHISA7	1
SHOC2	1
SIAH2	1
SIX3	1
SLA2	1
SLC10A3	1
SLC16A2	1
SLC19A3	1
SLC22A23	1
SLC24A2	1

SLC24A4	1
SLC25A1	1
SLC25A15	1
SLC25A53	1
SLC2A14	1
SLC2A3	1
SLC35C2	1
SLC35G1	1
SLC38A1	1
SLC38A2	1
SLC39A1	1
SLC39A14	1
SLC6A19	1
SLC6A5	1
SLC8A1	1
SLC9A7	1
SLCO3A1	1
SLITRK3	1
SMAD2	1
SMAD4	1
SMAP1	1
SMARCA5	1
SMG5	1
SMG7	1
SMUG1	1
SMURF1	1
SNX12	1
SNX18	1
SNX30	1
SON	1
SORCS1	1
SORL1	1
SORT1	1

SOX13	1
SOX5	1
SP8	1
SPAG9	1
SPATA2	1
SPATS2	1
SPECC1L	1
SPIN3	1
SPRY3	1
SPTY2D1	1
SRM	1
SRRD	1
SRRM4	1
SRSF1	1
SRSF12	1
SSBP2	1
SSX2IP	1
ST6GALNAC5	1
ST6GALNAC6	1
ST7L	1
ST8SIA2	1
STAC2	1
STARD13	1
STAU2	1
STC1	1
STK35	1
STK40	1
STRA13	1
STRBP	1
STX4	1
STXBP4	1
STXBP6	1
SUFU	1

SUPT6H	1
SVOP	1
SYNGAP1	1
SYNM	1
SYT1	1
SYT13	1
TAF3	1
TAF9B	1
TAPBP	1
TBC1D22B	1
TBL1X	1
TBL2	1
TBX19	1
TBX5	1
TCF12	1
TCF21	1
TDRKH	1
TEAD1	1
TENM2	1
TET2	1
TFAP2A	1
TFAP2C	1
TFRC	1
TGOLN2	1
THBS2	1
THSD7A	1
TIAF1	1
TIAM1	1
TIFAB	1
TJP1	1
TLCD2	1
TM9SF2	1
TMC5	1

TMEM120B	1
TMEM132B	1
TMEM136	1
TMEM167B	1
TMEM168	1
TMEM170B	1
TMEM183A	1
TMEM194A	1
TMEM200C	1
TMEM216	1
TMEM233	1
TMEM260	1
TMEM33	1
TMOD1	1
TMOD2	1
TNIK	1
TNPO3	1
TNRC6B	1
TNS4	1
TOM1L2	1
TOX3	1
TPD52	1
TPM3	1
TPP2	1
TPR	1
TRIM2	1
TRIM44	1
TRIM5	1
TRIM66	1
TRIM67	1
TRPC1	1
TSN	1
TSPAN9	1

TTC5	1
TTL	1
TTYH3	1
UBA6	1
UBE2B	1
UBE2I	1
UBE2W	1
UBXN7	1
UHMK1	1
UNC5B	1
UNC5D	1
UPF3B	1
URM1	1
USB1	1
USF2	1
USH1C	1
USP25	1
USP44	1
USP46	1
USP47	1
VASH1	1
VASN	1
VAT1	1
VIPAS39	1
VSTM5	1
VTI1B	1
WAPAL	1
WASF2	1
WASF3	1
WBP11	1
WDR12	1
WDR26	1
WDR52	1
WDR55	1
WISP2	1
WNK3	1
WNT9B	1
XIAP	1
XKR4	1
XRN1	1
YARS	1
YWHAZ	1
ZBTB20	1
ZBTB2	1
ZBTB26	1
ZBTB43	1
ZBTB8B	1
ZC2HC1C	1
ZC3H11A	1
ZC3H6	1
ZCCHC3	1
ZDHHC13	1
ZDHHC18	1
ZDHHC21	1
ZFAND5	1
ZFYVE1	1
ZMYND11	1
ZNF148	1
ZNF202	1
ZNF226	1
ZNF37A	1
ZNF385A	1
ZNF423	1
ZNF512B	1
ZNF532	1
ZNF549	1

ZNF608	1
ZNF652	1
ZNF704	1
ZNF81	1
ZNF827	1
ZNRF2	1

		Read Count (Avg)		Log2 Fold
**S.No.**	**piRNA**	**Mock Exo**	**RSV Exo**	**Change**
1	piR-32678	127.41	27150.88	7.7353
2	piR-31623	0.62	48.19	6.2854
3	piR-34736	1.74	128.93	6.214
4	piR-36169	6.52	231.03	5.1477
5	piR-36170	6.52	231.03	5.1477
6	piR-49143	22.5	590.86	4.7148
7	piR-33748	7.13	108.2	3.9227
8	piR-45371	7.13	108.2	3.9227
9	piR-49144	126.89	1387.4	3.4508
10	piR-33043	33.7	291.08	3.1105
11	piR-33044	33.7	291.08	3.1105
12	piR-34531	5.88	24.21	2.0418
13	piR-34532	5.88	24.21	2.0418
14	piR-34533	5.88	24.21	2.0418
15	piR-34534	5.88	24.21	2.0418
16	piR-34535	5.88	24.21	2.0418
17	piR-34536	5.88	24.21	2.0418
18	piR-51809	4.51	17.28	1.9379
19	piR-51810	4.51	17.28	1.9379
20	piR-51811	4.51	17.28	1.9379
21	piR-58469	8.52	27.25	1.6768
22	piR-61298	10.6	29.04	1.4536
23	piR-34871	26.03	64.15	1.301
24	piR-57125	46.18	110.77	1.2622
25	piR-35549	13.21	30.88	1.2251
26	piR-35550	13.21	30.88	1.2251
27	piR-35551	13.21	30.88	1.2251
28	piR-54265	18.53	39.57	1.0942
29	piR-59169	1364.92	6.02	-7.8241
30	piR-50653	22.7	0.13	-7.4733
31	piR-50654	22.7	0.13	-7.4733
32	piR-50655	22.7	0.13	-7.4733
33	piR-55865	22.7	0.13	-7.4733
34	piR-56226	22.7	0.13	-7.4733
35	piR-34756	10411.18	82.62	-6.9775
36	piR-34370	28.39	0.29	-6.6371
37	piR-45065	21.57	0.29	-6.2412
38	piR-40249	50.07	0.74	-6.0707
39	piR-40514	59.65	1.03	-5.8557

40	piR-36037	85.04	1.78	-5.5788
41	piR-34916	29.19	0.63	-5.5244
42	piR-56647	40.04	1.03	-5.2807
43	piR-59089	10.75	0.29	-5.2366
44	piR-47700	62.72	3.49	-4.1692
45	piR-30528	69.24	3.99	-4.116
46	piR-58651	69.24	3.99	-4.116
47	piR-30606	116.43	6.73	-4.1118
48	piR-36001	116.43	6.73	-4.1118
49	piR-34983	32.87	2.12	-3.9518
50	piR-45201	32.87	2.12	-3.9518
51	piR-33497	39.01	2.58	-3.9163
52	piR-61644	71.79	10.79	-2.7334
		**Read Count (Avg)**		
**S.No.**	**piRNA**	**Mock Exo**	**RSV Exo**	
1	piR-31703	0	254.19	N/A
2	piR-33650	0	209.91	N/A
3	piR-40982	0	209.39	N/A
4	piR-32079	0	209.21	N/A
5	piR-31961	0	208.91	N/A
6	piR-33527	0	153.92	N/A
7	piR-33526	0	153.02	N/A
8	piR-36716	0	127.96	N/A
9	piR-36715	0	127.67	N/A
10	piR-36714	0	127.39	N/A
11	piR-36494	0	127.01	N/A
12	piR-36713	0	125.77	N/A
13	piR-35059	0	124.97	N/A
14	piR-34608	0	118.37	N/A
15	piR-48517	0	118.37	N/A
16	piR-60668	0	58.07	N/A
17	piR-61919	0	46.01	N/A
18	piR-31701	0	36.7	N/A
19	piR-34221	0	21.87	N/A
20	piR-61950	19.15	0	N/A
21	piR-59554	13.59	0	N/A
22	piR-61974	11.12	0	N/A

Supplementary Table 5. Complete list of piRNAs whose expression in exosomes was changed following RSV infection.

Fig 2(b) left panel

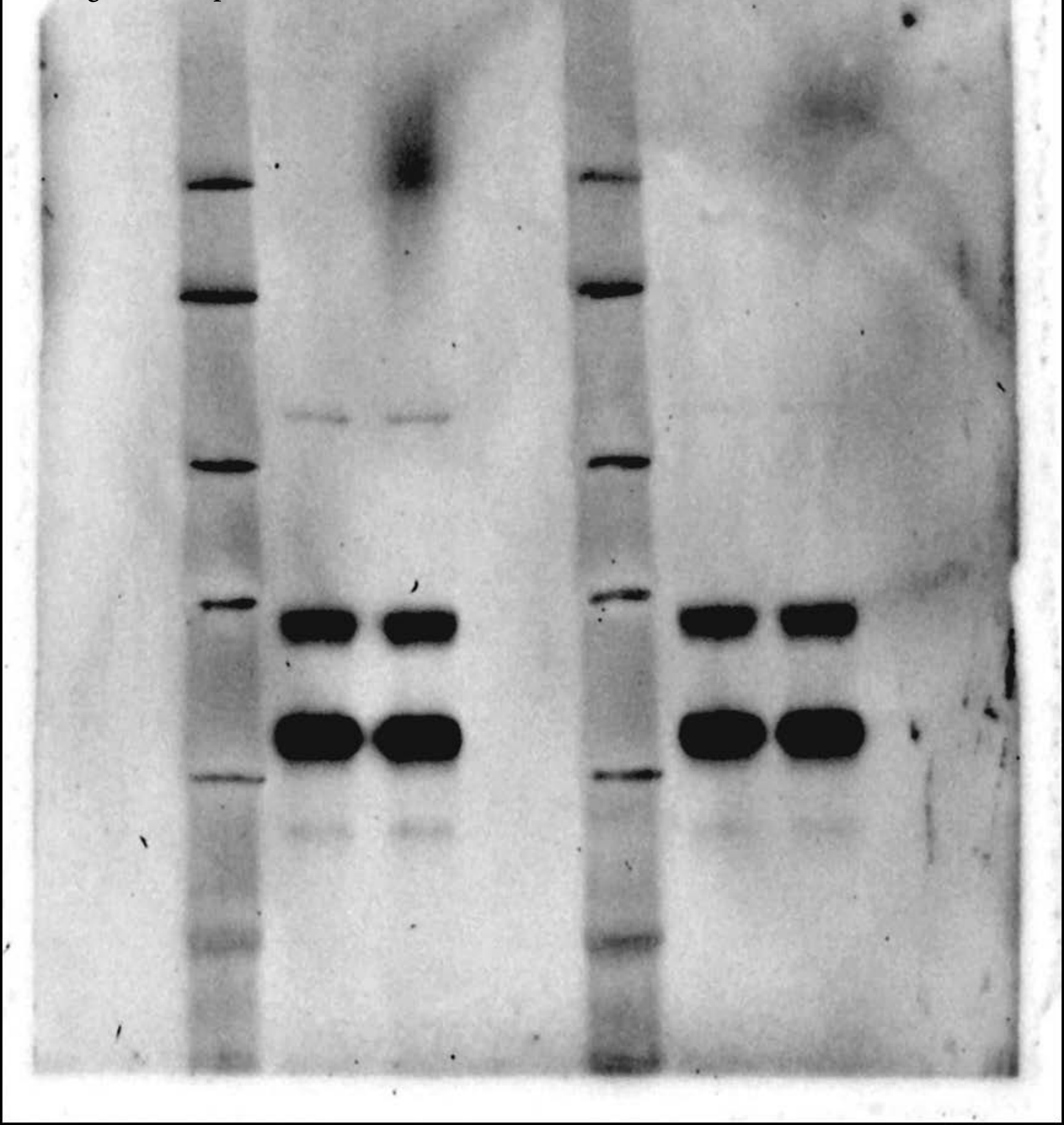

Fig 2(b) right panel

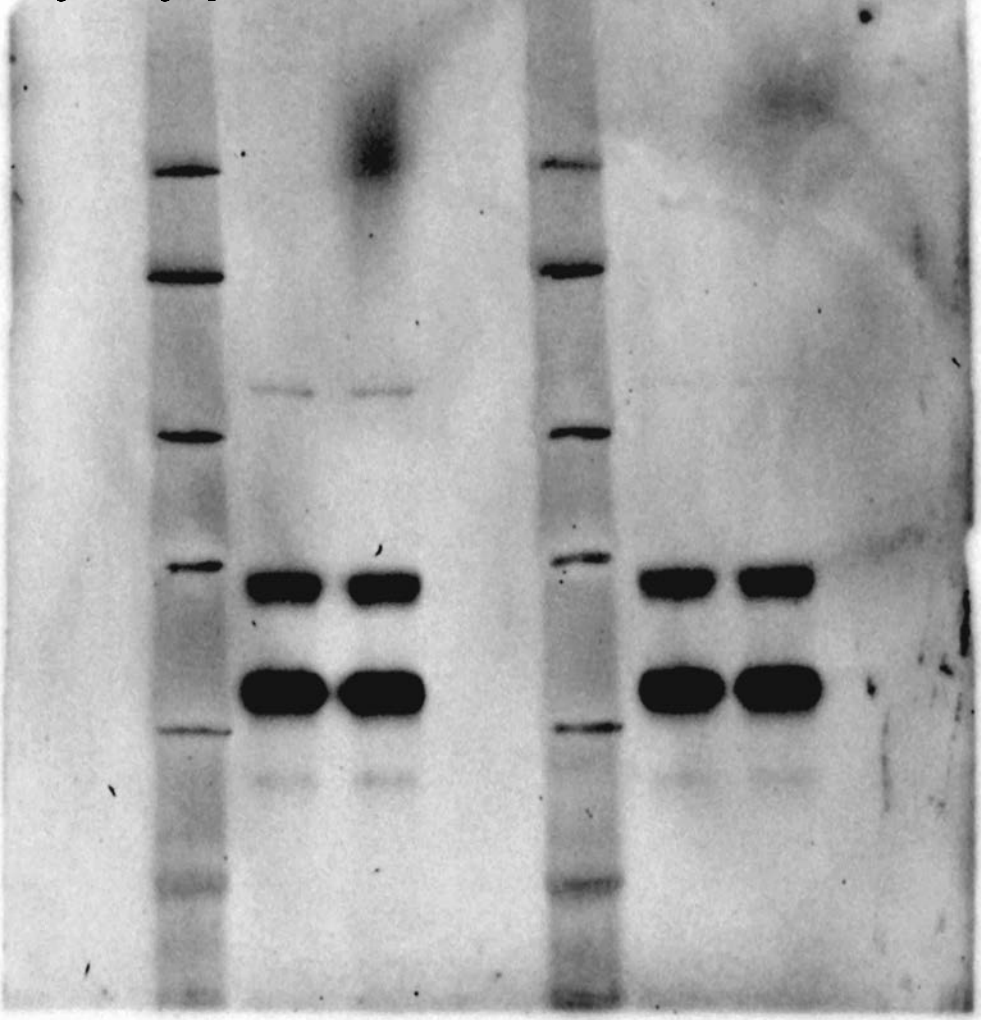

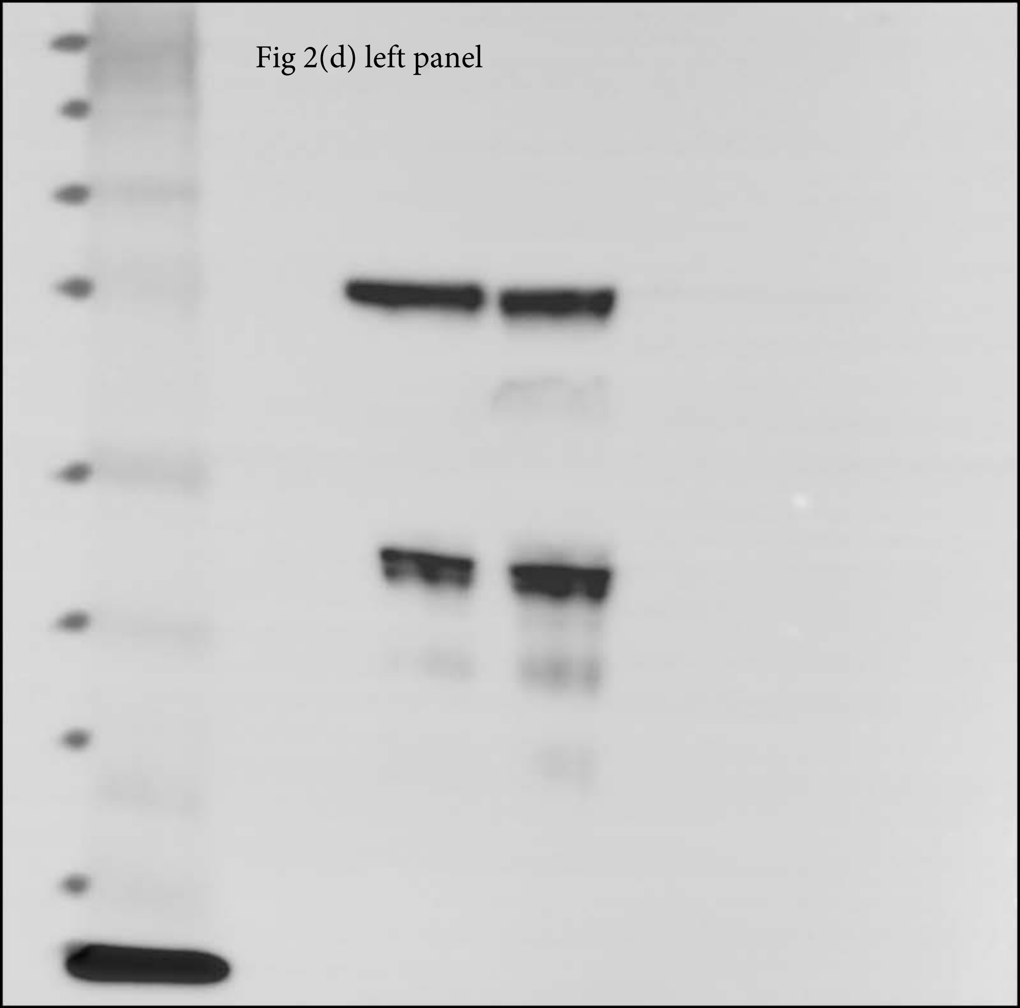
Fig 2(d) left panel

Fig 3 top

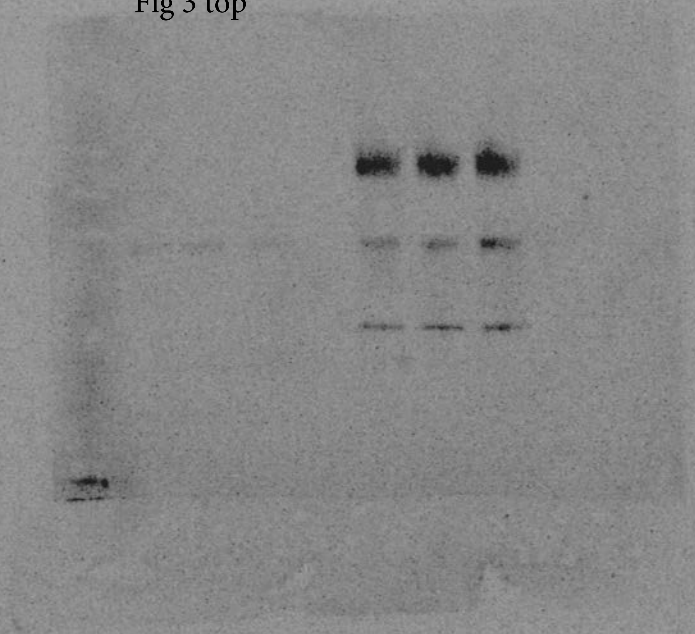

Fig 3 bottom

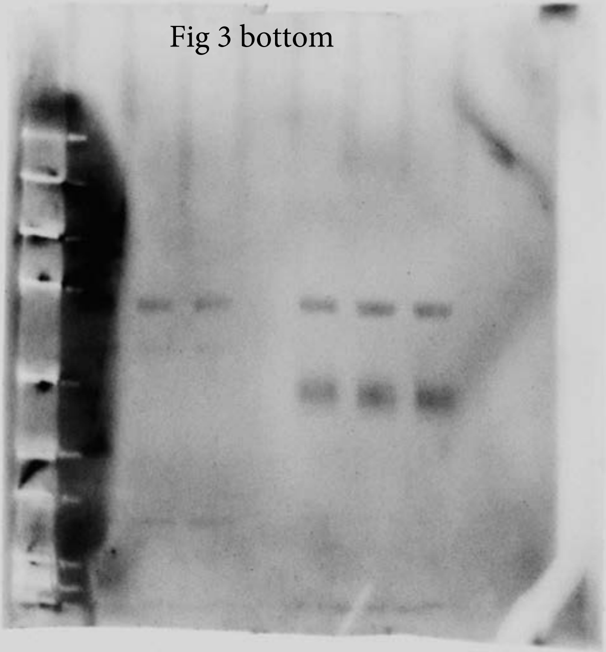

Supplement: Supplementary file 1 — Supplementary Information [file 41598_2017_18672_MOESM1_ESM.pdf]
